# Genome-Wide Association Studies for Body Conformation Traits in Korean Holstein Population

**DOI:** 10.3390/ani13182964

**Published:** 2023-09-19

**Authors:** Md Azizul Haque, Mohammad Zahangir Alam, Asif Iqbal, Yun-Mi Lee, Chang-Gwon Dang, Jong-Joo Kim

**Affiliations:** 1 Department of Biotechnology, Yeungnam University, Gyeongsan 38541, Gyeongbuk, Republic of Korea; azizul@ynu.ac.kr (M.A.H.); mzalam-geb@sust.edu (M.Z.A.); asif-gen@sust.edu (A.I.); ymlee@yu.ac.kr (Y.-M.L.); 2 Animal Breeding and Genetics Division, National Institute of Animal Science, Cheonan 31000, Chungcheongnam-do, Republic of Korea

**Keywords:** body conformation traits, candidate gene, genome-wide association study, Korean Holstein

## Abstract

**Simple Summary:**

Holstein has been the most widely used dairy cattle breed in the Korean Peninsula since its introduction in 1885. Since the formal dairy herd improvement program was initiated in 1979, Holsteins have been extensively selected for Korean environments. While body conformation traits in dairy cattle are not typically considered direct economic traits for animal breeders, they are associated with various production traits, including health status, milking ability, and overall cow longevity. Consequently, these traits are recognized as significant factors in dairy cattle breeding programs worldwide. The dairy industry relies on consistent milk and calf production from cows, which presents challenges in today’s context. Conducting a genome-wide association study (GWAS) for body conformation traits in Korean Holstein cattle, using a large population, may enhance the power of detecting and mapping novel genes associated with these traits. To the best of our knowledge, this study represents the first-ever attempt to conduct a GWAS for body conformation traits in the Korean Holstein population.

**Abstract:**

The objective of this study was to identify quantitative trait loci (QTL) and nearby candidate genes that influence body conformation traits. Phenotypic data for 24 body conformation traits were collected from a population of 2329 Korean Holstein cattle, and all animals were genotyped using the 50 K Illumina bovine SNP chip. A total of 24 genome-wide significant SNPs associated with 24 body conformation traits were identified by genome-wide association analysis. The selection of the most promising candidate genes was based on gene ontology (GO) terms and the previously identified functions that influence various body conformation traits as determined in our study. These genes include *KCNA1*, *RYBP*, *PTH1R*, *TMIE*, and *GNAI3* for body traits; *ANGPT1* for rump traits; *MALRD1*, *INHBA*, and *HOXA13* for feet and leg traits; and *CDK1*, *RHOBTB1*, and *SLC17A1* for udder traits, respectively. These findings contribute to our understanding of the genetic basis of body conformation traits in this population and pave the way for future breeding strategies aimed at enhancing desirable traits in dairy cattle.

## 1. Introduction

Cattle represent a unique and singular species on earth, holding a remarkable position due to their ability to fulfill a wide range of essential human needs. These needs encompass food, clothing, draught power, medicine, soil improvement fertilizer, fuel energy, and more. Additionally, cows play important roles in religious and cultural practices, particularly in India, where the largest cattle population is found. In fact, according to the US Department of Agriculture and the Food and Agriculture Organization (FAO), the global cattle population reached approximately 1.5 billion heads in 2022. The genetic improvement of high-yielding animals reduces the strain on natural resources and contributes to mitigating global warming. These areas of study have captured the interest of geneticists and other scientific communities. 

In 2009, the sequencing of the bovine genome coupled with advancements in information technology facilitated the development of modern, scientifically designed breeding schemes for enhancing economically important traits. These efforts led to a remarkable increase in both the quality and quantity of milk and meat production per animal [1]. It was hypothesized that the application of genomic selection (GS) would significantly accelerate breeding progress, representing a paradigm shift in breeding practices. The dairy industry, in particular, fulfilled the necessary conditions to yield positive results [2]. The application of genomic selection has become a prevalent tool in the dairy sector, resulting in substantial genetic gains [3]. Understanding essential genes, haplotypes, and their regulatory mechanisms as markers for quantitative traits could improve strategies for selecting dairy cattle both presently and in the future [4]. In this regard, genome-wide association studies (GWAS) play a key role by analyzing diverse dairy traits to identify significant quantitative trait loci (QTL) and single-nucleotide polymorphism (SNP) markers [5]. To date, the animal QTLdb has systematically cataloged 983 publications and 130,407 QTLs for cattle, making it the largest database for livestock species [6]. Most SNPs associated with specific traits are believed to be in linkage disequilibrium with as-yet-unknown causative mutations. Identifying functionally relevant DNA mutations is vital for effective genomic selection, and it necessitates a comprehensive understanding of numerous genes associated with each known QTL.

Holsteins, renowned for their exceptional milk production capacity, represent the most popular dairy cattle breed worldwide. In Korea, this breed is also a major source of milk and milk products. The dairy cattle industry in Korea focuses on developing advanced herd management programs aimed at maximizing production and profitability and minimizing methane emissions. However, the sudden culling of cows from the herd often hampers these efforts. To address this, the Korea Animal Improvement Association (KAIA) has set general appearance and linear examination procedures for scoring cows and ranking them based on their obtained scores, with the objective of selecting the best dairy cows. This study considers 24 body conformation traits screened by KAIA, which were subjected to GWAS analysis. 

The evaluation of dairy cattle primarily relies on the observation of certain physical characteristics associated with longevity and production traits. These traits, collectively known as body conformation traits, have been utilized in dairy cattle judging in many countries since the 1990s [7]. In the dairy industry, profitability is solely dependent on the milk production capacity of cows. Therefore, an ideal cow should possess sound health, fertility, and an extended lifespan to sustain a productive dairy system. Body conformation traits offer important phenotypic information about dairy cattle, providing insights into functional body shapes that affect milk production. Several studies have indicated a genetic correlation between body conformation traits and economically significant traits such as calving ease, longevity, lameness, and overall economic value [8]. Furthermore, certain body conformation traits such as stature and body depth have been found to be genetically correlated with reproduction traits such as gestation length, calving interval, and days from calving to first insemination [9]. A body condition score assessment is employed to evaluate the energy reserve of dairy cows, which in turn is associated with their energy balance status and reproductive capability. Previous research has employed multiple analyses to determine the genetic correlation between reproductive ability and body condition score. The findings from these studies have indicated that dairy cows with a lower body condition score often exhibit reduced reproductive ability [10].

Recent years have seen a significant focus on conducting GWAS to investigate economic traits in dairy cattle, including fertility traits [11,12,13], production traits [14,15,16], somatic cell score [17], and disease resistance [18,19]. Numerous statistically significant SNPs and biologically meaningful genes have been reported. However, comparatively fewer studies have explored the relationship between body conformation traits and dairy cattle. Some notable studies include those on Czech Holsteins [6], Chinese Holstein cattle [7], Canadian Holsteins [8], US Holsteins [11], Mexican Holsteins [20], and Australian Holsteins [21]. Some researchers have employed linkage analysis to detect QTLs associated with conformation traits [22,23,24]. However, the genes identified for body conformation traits in these reports exhibited discordance, potentially arising from the use of different bovine genome assemblies for mapping genes, variations in population size, or differences in statistical methods employed in GWAS. There is only one study available on Korean body conformation traits, which primarily focuses on estimating genetic parameters [25]. Consequently, the exploration of the genetic architecture of these body conformation traits within this breed would provide baseline information regarding the QTLs or genes underlying the traits under investigation. As far as we are aware, this marks the first attempt at unraveling the genetic composition of body conformation traits in Korean Holstein cattle. Therefore, this study aims to identify significant SNPs (QTLs) associated with our studied traits and further map the underlying promising genes responsible for these traits.

## 2. Materials and Methods

### 2.1. Ethics Statement

The care and management of all animals used in this study were approved by the Animal Care and Use Committee of the National Institute of Animal Science (NIAS), Rural Development Administrations (RDA), South Korea (Approval No. 2016-189, approval date: 9 June 2016). All animal health and welfare practices followed the approved guidelines.

### 2.2. Animal Management and Phenotypes

The first parity phenotypic data were collected from 2329 Holstein dairy cattle located in Nonghyup livestock farms in different regions of Korea. The data were recorded between 2017 and 2018. There were 24 traits measured, including stature, height at front end, chest width, body depth, angularity, body condition score, locomotion, rump angle, rump width, loin strength, rear leg set, rear leg rear view, foot angle, heel depth, bone quality, udder depth, udder texture, udder support, fore udder attachment, front teat placement, front teat length, rear udder height, rear udder width, and rear teat placement. The phenotypic information was recorded for these 24 body conformation scores based on the guidelines provided by the Korean Animal Improvement Association (KAIA). The body conformation traits included 24 linear descriptive traits, which were scored on a scale from 1 (indicating the poorest body conformation) to 9 (indicating the best body conformation). The details of phenotypic distribution information for the studied animals are presented in Supplementary Material Figure S1.

### 2.3. Genotyping and Quality Control

A total of 2329 Holstein dairy cows were genotyped using the Illumina Bovine SNP 50 K v.3 BeadChip (Illumina Inc., San Diego, CA, USA), which contained a total of 49,203 embedded SNPs. SNPs located on sex chromosomes and with unknown and duplicate positions were removed for further quality control (QC) procedures. Several QC thresholds were set to remove poor-quality SNPs for further analysis. SNPs were discarded from the analysis when the SNP call rate was less than 90%, for individuals with a genotyping call rate less than 90%, and when minor allele frequency (MAF) was less than 1% (monomorphic). The genotype frequency significantly deviated (*p* < 0.000001) from Hardy–Weinberg Equilibrium (HWE). The identity-by-state (IBS) test was performed to determine if there were similar individuals or genotyping errors in the datasets. Pairs of individuals showing a similarity rate greater than 99% were considered either identical animals or indicative of genotyping errors. The entire QC process and IBS test were performed through the PLINK v1.9 toolset [26]. Furthermore, the missing alleles were imputed using Beagle v5.4 software [27]. After conducting the QC and IBS tests, a total of 38,720 SNPs and 2206 animals remained for further analysis.

### 2.4. Population Structure Analysis

A four-dimensional pairwise genetic distance matrix was computed using the multi-dimensional scatter (MDS) plot algorithm in the PLINK v1.9 toolset [26]. The resulting coordinates were then visualized in R software v4.2.0 [28]. MDS analysis, as illustrated in Supplementary Figure S2, was used to capture the preliminary glimpse of the genetic structure of the Korean Holstein populations and to identify and exclude any potential outliers.

### 2.5. Statistical Analysis

The statistical significance of the fixed factors and covariates were tested using ASReml-SA v4.2 [29] for fitting the factors into the animal model. For body conformation traits, we fitted three age group (30, 35, and 35 month over), and farm, birth year, and birth season combined into one (farm_birth_year_birth_season) of the animals as fixed effects in the animal model for ASReml-SA (average sparsity residual maximum likelihood) analysis [29]. The single-trait animal model was implemented for GBLUP as follows [30]:

y=Xb+Zu+e

where y is the vector of phenotypes; b is the vector of fixed effects; u is the vector of random genetic additive effects; and e is the vector of random residual effects, which is assumed to be normally distributed with N (0, $\sigma_{e}^{2})$. The X and Z are incidence matrices associating b and u to y. In matrix notation, the mixed model equation (MME) could be written as:

$$\left[\begin{array}{cc} X^{\prime }X & X^{\prime }Z\\ Z^{\prime }X & Z^{\prime }Z+G^{-1}\alpha \end{array}\right]\left[\begin{array}{c} \hat{b}\\ \hat{a} \end{array}\right]=\left[\begin{array}{c} X^{\prime }Y\\ Z^{\prime }Y \end{array}\right]$$

where $\alpha =\sigma_{e}^{2}/\sigma_{g}^{2}$, $\sigma_{g}^{2}$ is the genetic variance, and $\sigma_{e}^{2}$ is the error variance.

The genomic relationship matrix (G) was built using the genome-wide complex trait analysis (GCTA) tools developed by Yang et al. [31], which efficiently represents the genomic relationships between animals [30]. The following formula was used to create the G-matrix:

$$G=\frac{\left(M-P\right){\left(M-P\right)}^{\prime }}{2\;\sum_{i=1}^{n}P_{i}\left(1-P_{i}\right)}$$

where the marker matrix M has dimensions of n × m, n is the number of individuals, and m is the number of markers used. The marker alleles M were coded as AA (homozygous for the first allele) = 1, AB (heterozygous) = 0, and BB (homozygous for the second allele) = −1. The element of P matrix was calculated using the formula $P_{i}=2\left(P_{i}-0.5\right)$, where $P_{i}$ represents the minor allele frequency of the marker at locus i. M-P represents the incidence matrix (Z) for markers.

#### Genome-Wide Association Study

For GWAS analysis according to Aulchenko et al. [32], the residuals (obtained from a mixed model analysis by ASReml-SA v4.2) were used to estimate by fitting following single-marker regression analysis:

$$y=1_{n}\mu +\mathrm{Zg}+e$$

where y is the residual of the phenotype, $1_{n}$ is a vector of 1 s, μ is the overall mean, Z is the design matrix allocating to the records of the marker, g denotes the marker effects, and e is the vector of random residuals. In this model, the marker effects are fitted as fixed effects. It is worth noting that g is a vector whose size equals the number of SNP marker alleles since we only estimated additive effects. In the additive model, the SNP genotypes were coded as 1, 0, and −1 for the AA, AB, and BB genotypes, respectively, indicating the allele substitution effect of B on allele A.

The incorporation of categorical data within the GRAMMAR-based GWAS [32] framework represents a pivotal aspect of our study. This approach allows us to explore the genetic associations underlying complex categorical traits observed in Korean Holstein cows. In order to gain a more comprehensive understanding of the residuals data structure utilized in this study, we conducted a detailed examination of normality using the Shapiro–Wilk test [33]. This statistical test evaluates whether the residuals follow a normal distribution. For the assessment of residuals, we generated both a histogram and a density plot, and the quantile-quantile (QQ) plot displaying their actual values is commonly referenced (Supplementary Material Figure S3). 

The PLINK v1.9 toolset [26] was used to estimate the SNP effects by regressing the residuals of each phenotype on additive effects of each SNP using the ordinary least square (OLS) method, and the *p*-value for the regression coefficient was estimated. If the SNP marker has a significant effect on the trait, then it can be assumed that the SNP was in a linkage disequilibrium (LD) state with an unobserved QTL. For the statistical significance test of the effect of the SNP marker (QTL) on the trait, the null hypothesis (H_0_) assumes no QTL effect, while the alternative hypothesis assumes that QTL has an effect on the trait. The significance of the test’s statistic *p*-value (genomic control) threshold was set at α = 0.001 (i.e., SNPs with a *p*-value (*p* < 0.001, −log_10_P = 3) were regarded as significant SNPs). 

### 2.6. Bioinformatics Analysis

#### 2.6.1. Detection of Significant SNPs and Nearby Candidate Genes

We set a significant threshold above the suggestive level (1/number of variants), that is, (2.58 × 10^−5^) or 4.59 (−log_10_P), in order to identify genome-wide significant SNPs influencing the body conformation traits in Korean Holstein, according to Guo et al. [34]. Although the suggestive significance level may potentially lead to false positive results, we made an effort to encompass all plausible loci that could underlie body conformation traits in Holstein. Consequently, we adopted a less strict threshold to reduce the chance of missing potential markers associated with conformation traits through GWAS. In addition to the suggestive significance level, we also applied the Bonferroni correction (0.05/number of variants), equivalent to (1.29 × 10^−6^) or 5.89 (−log_10_P).

Putative candidate genes within the QTL regions and in the nearest upstream and downstream regions (500 kb) of our mapped significant SNPs were identified based on the bovine (Bos_taurus_UMD_3.1.1) genome assembly, using online resources such as Genome Data Viewer (https://www.ncbi.nlm.nih.gov/genome/gdv/?org=bos-taurus; accessed on 15 June 2023), BovineMine V1.6 (an integrated data warehouse for the Bovine Genome Database, http://128.206.116.13:8080/bovinemine/begin.do, accessed on 17 June 2023), and BGVD (Bovine Genome Variation Database and Selective Signatures, found at the link http://animal.nwsuaf.edu.cn/code/index.php/BosVar, accessed on 21 June 2023).

#### 2.6.2. Functional Annotations

The candidate genes obtained through GWAS were submitted into the Database for Annotation, Visualization, and Integrated Discovery (DAVID) [35]. The purpose was to conduct a comprehensive analysis of gene ontology (GO) terms [36] and Kyoto Encyclopedia of Genes and Genomes (KEGG) pathways [37]. For both functional analysis and pathway analysis, the statistically significant *p*-value threshold was set at *p* ≤ 0.05. This approach allowed us to explore the potential biological functions and pathways associated with the identified genes.

## 3. Results and Discussion

### 3.1. Summary Statistics of the Phenotypes

The summary statistics for the 24 body conformation traits of the Korean Holstein population are presented in Table 1. These traits were further classified into four major categories: body traits (including stature, height at front end, chest width, body depth, angularity, body condition score, and locomotion), rump traits (comprising rump angle, rump width, and loin strength), feet and leg traits (encompassing rear leg set, rear leg rear view, foot angle, heel depth, and bone quality), and udder traits (including udder depth, udder texture, udder support, fore udder attachment, front teat placement, front teat length, rear udder height, rear udder width, and rear teat placement). The average scores for the body traits ranged from 4.39 to 6.81, while the rump traits exhibited a mean range of 4.58–5.53. The studied population demonstrated average scores for feet and leg traits, ranging from 5.01 to 5.62. In contrast, the average scores for udder traits varied from 4.30 to 6.88. Among these traits, the highest coefficient of variation (32.18%) was observed in rear udder width, while the lowest (12.23%) was observed in height at front end.

### 3.2. GWAS for Body Conformation Traits

The genome-wide association study for body conformation traits identified a total of 24 significant SNPs distributed across all 29 *Bos taurus* autosomes (BTA). However, the distribution was uneven, with certain chromosomes showing a higher number of SNPs associated with different traits. Among the chromosomal regions, BTA22 had the highest number of identified SNPs, with a total of five. Additionally, BTA3, BTA4, BTA13, and BTA14 exhibited two, two, two, and three significant SNPs, respectively. 

To visualize the genome-wide distribution of significant SNPs, we generated Manhattan plots for body traits, rump traits, feet and leg traits, and udder traits. The level of significance was represented as the negative logarithm base 10 (−log_10_) of each SNP’s *p*-value. The statistical significance was performed on the two levels, 2.58 × 10^−5^ (4.59) for the suggestive significance threshold and 1.29 × 10^−6^ (5.89) for the genome-wide Bonferroni correction significance threshold. After the Bonferroni correction, no SNPs exhibited statistically significant associations with the body conformation traits. To assess population stratification, we calculated the genomic inflation factor, Lambda (λ), by comparing the median chi-squared test statistics obtained from a GWAS to the expected median of the chi-squared distribution. The approach for calculating lambda may vary based on the association analysis output [38]. In our study, we used *p*-values from the GWAS results of all traits and computed λ using the qchisq() function in R [28]. Additionally, we depicted Q-Q plots to illustrate the observed versus expected *p*-values (−log_10_P) for each trait. The genomic inflation factor should be close to 1 after correcting for population stratification [39]. In this study, the QQ plots clearly illustrate that the observed values closely align with the expected values, with the inflation factor (λ) ranging from 0.992 to 1.022 across all 24 body conformation traits (Supplementary Material Figures S5, S7, S9 and S11). These findings indicate that the population stratification was properly corrected by using an appropriate model. The very high value of the genomic inflation factor suggests that other factors, such as high linkage disequilibrium, strong association between phenotypic traits and SNPs, or systematic technical bias, might also contribute to the observed inflation [40]. A detailed summary of the GWAS results, including significant SNP IDs for the studied traits, SNP positions on the respective BTAs, effects on the traits, *p*-values, and nearby candidate genes is provided as follows.

#### 3.2.1. Body Traits

In the studied Korean Holstein population, a total of 10 significant SNPs were detected for various body traits. Specifically, stature was associated with two significant SNPs; for height at the front end and chest width, each trait was associated with one SNP; and for body depth and angularity, each trait was associated with three SNPs. This study reported no significant SNPs (Bonferroni-corrected and suggestive significance thresholds) for body condition score and locomotion traits. The significant markers identified through our GWAS analysis are presented in Table 2. In addition, Supplementary Material Figure S4 displays the Manhattan plots illustrating the genome-wide distribution of these significant SNPs that underlie our selected body traits. Moreover, QQ plots were created to observe the extent to which the observed *p*-values deviate from the expected *p*-values under the null hypothesis, assuming no association (Supplementary Material Figure S5).

For the stature trait, we identified two noteworthy SNPs through our GWAS analysis. Firstly, Hapmap60480-ss46526970 exhibited a *p*-value of 1.18 × 10^−5^. This SNP is situated between the *NDUFA9*, *KCNA1,* and *KCNA6* genes on BTA5, serving as an intergenic variant. Secondly, another significant SNP, Hapmap50195-BTA-105299, showed a *p*-value of 6.34 × 10^−6^. This particular SNP was found within the *LOC107132946* gene, and also as an intergenic variant on BTA11 (Table 2). The *KCNA1* gene encodes the voltage-gated potassium channel Kv1.1 protein, which plays a major role in refractory focal epilepsy disease in humans and is essential for proper brain development [41]. Furthermore, the recently identified variants underscore emerging connections between *KCNA1* and musculoskeletal abnormalities [42]. 

Regarding height at the front end, one SNP (ARS-BFGL-NGS-20317, *p*-value = 7.90 × 10^−6^) was identified as intergenic variants of the *RYBP* gene on BTA22 (Table 2). Researchers found that the *RYBP* gene plays a significant role in the development of skeletal muscles in cattle and in the differentiation of myoblasts [43]. Furthermore, *RYBP* has the potential to enhance the body measurement traits in beef cattle, thereby expediting breeding efforts focused on improving these traits within the Qinchuan cattle breed [44]. The expression of MRF family members is integral to normal muscle differentiation. Notably, during both prenatal and postnatal muscle development stages, Wnt ligands regulate the expression of MRF family members. This regulation activates a signaling pathway responsible for overseeing muscle growth, myogenesis, and the creation of multinucleated myotubes. For instance, Wei et al. [45] have demonstrated that genes within this family not only facilitate skeletal muscle growth through the regulation of myogenic regulatory factors such as MYOD and myogenin, but also contribute to an enhancement in meat quality.

For chest width, we identified a total of two genes, namely *CCDC12* and *PTH1R*, that were found within or nearby the significant SNPs (BTB-00853109, *p*-value = 1.52 × 10^−5^) on BTA22. One particularly noteworthy gene is the *PTH1R* gene, which has been identified as a candidate gene, both functionally and positionally, in relation to traits associated with leg weakness in pigs [46]. The *PTH1R* gene is responsible for mediating the effects of parathyroid hormone, and in turn plays a crucial role in the endochondral bone formation process [47]. One of the key functions of the *PTH1R* gene is its role in regulating the growth of cartilage and the apoptosis of chondrocytes [48]. Furthermore, the *PTH1R* gene plays a significant role in the development of the skeletal system and is closely associated with the occurrence of osteoarthritis in mice [49,50].

In the analysis of body depth, a total of three SNPs were identified on the BTA22 (namely, BTB-00853109 with a *p*-value of 1.99 × 10^−5^, Hapmap43881-BTA-54837 with a *p*-value of 2.02 × 10^−5^, and ARS-BFGL-NGS-117960 with a *p*-value of 3.20 × 10^−6^). Interestingly, the locus BTB-00853109 was identified at the 53.16 Mb genomic position on BTA22, showcasing an intriguing association with both chest width and body depth traits in Korean Holstein cattle. This overlap of genetic influence on multiple traits is referred to as pleiotropy, a phenomenon in line with the strong positive genetic correlation observed between these two traits. Furthermore, the common locus influencing both chest width and body depth suggests a potential shared genetic architecture for these traits. This finding opens up an intriguing avenue for future research. It necessitates the exploration of these genomic regions on a larger scale for validation. Moreover, there is a need to delve into the further implications of these SNPs in marker-assisted animal breeding. We identified a total of five putative candidate genes, namely *CCDC12*, *PTH1R*, *PRSS45*, *PRSS46*, and *TMIE*, surrounding our spotted significant marker loci for body depth. Among them, the *TMIE* gene, which is located 53.35 Mb immediate next to our mapped SNP locus (ARS-BFGL-NGS-117960) on BTA22, has been identified as associated with sensory cell development across vertebrate species [51]. *TMIE* exhibits expression not only in the developing inner ear but also in other tissues, including the brain, liver, kidney, and lung [52].

Additionally, three SNPs significantly associated with the angularity trait were located on BTA3 and BTA10, within the *GNAI3* and *AP3B1* genes, respectively. The G protein subunit alpha i3 (*GNAI3*) is a gene with the potential to play a significant role in goats’ heat tolerance mechanisms [53]. The expression of the *GNAI3* gene in sclerotomal derivatives is essential for establishing the normal pattern of the axial skeleton [54]. The observation that rib fusions are restricted to the cartilaginous, distal portion of the ribs in *GNAI3* knockout mice, with apparently normal development of the bony proximal ribs, suggests that the primaxial/abaxial classification system in somitic development could shed light on the function of Gαi [55,56]. Increasing evidence supports the involvement of *GNAI3* in various cellular processes, including proliferation, apoptosis, cytokinesis, and differentiation [57,58,59]. Furthermore, *GNAI3* has been implicated in the regulation of craniofacial growth and development. Additionally, alterations in the human *GNAI3* gene can result in auriculo-condylar syndrome (ACS), a rare craniofacial congenital disorder characterized by mandible hypoplasia [60]. 

#### 3.2.2. Rump Traits

A total of four significant SNPs associated with rump traits were detected on BTA14 and BTA17. In our GWAS analysis of Korean Holstein, no significant SNPs were found for rump angle and loin strength among the rump traits, even after applying Bonferroni correction and considering the suggestive significance threshold. The whole-genome association of noteworthy SNPs associated with rump traits in Holstein cattle are presented in Table 2. To visually depict the distribution of significant SNPs across the genome for rump traits in Holstein, a Manhattan plot was created (Supplementary Material Figure S6). Furthermore, a QQ plot was generated to assess the deviations of observed *p*-values from expected *p*-values, assuming no association (Supplementary Material Figure S7).

Regarding rump width, three significant SNPs were located within the intergenic variant regions of the genes *LOC104974091*, *LOC614209*, *ANGPT1*, and *LOC782496* on BTA14. Additionally, one SNP was found in the upstream region of the *OSBP2* gene. Among the potential candidate genes that have been identified, *ANGPT1* stands out. This gene plays a significant role in regulating blood vessels within the bone marrow of mice [61]. A noteworthy study had previously proposed that bone cells are responsible for producing *ANGPT1*, which in turn supports and oversees the upkeep of stem cells within their designated environment. *ANGPT1* carries out the crucial task of minimizing blood vessel permeability, which could be achieved by enhancing the connections between endothelial cells. Furthermore, *ANGPT1* plays a vital role in the developmental process of blood vessels in mice [62]. 

#### 3.2.3. Feet and Leg Traits

The GWAS analysis conducted on feet and leg traits in the Korean Holstein population dataset revealed a total of four significant SNPs (Table 2). Among these, one SNP with rear leg rear view, one SNP with heel depth, and two SNPs with bone quality were identified. However, there is no significant SNP that meets the Bonferroni-corrected and suggestive significance threshold for rear leg set and foot angle. Out of the four SNPs, three were located within intergenic variants of three known genes, while another one was categorized as upstream gene variants of known and uncharacterized gene locations. Furthermore, Supplementary Material Figure S8 presents Manhattan plots illustrating the genome-wide distribution of the significant SNPs that underlie our selected feet and leg traits. Additionally, QQ plots were generated to observe the extent to which the observed *p*-values deviated from the expected *p*-values under the null hypothesis, assuming no association (Supplementary Material Figure S9).

Regarding rear leg rear view, one significant SNP (ARS-BFGL-NGS-629 with a *p*-value of 1.05 × 10^−5^) was detected within intergenic variants of *MALRD1* gene on BTA13. The *MALRD1* gene plays an essential role in intestinal cellular proliferation and growth that offers a potential target for future research [63].

In the case of heel depth, no SNPs were located within intron regions of known genes. Only one significant SNP (BTB-01928726, *p*-value = 2.08 × 10^−5^) was mapped on BTA4 and was found within genes such as *INHBA* and *LOC104972162*. Inhibin β A (*INHBA*) plays a role in regulating pituitary follicle-stimulating hormone levels. Researchers have identified the genes responsible for encoding inhibins α and β (*INHA*, *INHBA*) as potential candidates for analyzing their association with fertility in Hanoverian horses. Consequently, in Hanoverian horses, single-nucleotide polymorphisms (SNPs) and haplotypes within the *INHBA* gene have been reliably linked to the pregnancy rate per estrus and the embryonic and paternal components of breeding values [64,65,66]. The *INHBA* gene serves as a marker for granulosa cell development and exhibits expression in various ovarian cell types [67]. This includes oocytes in all follicle types, granulosa cells following the primary follicular phase, theca cells in sinus follicles, and surface epithelial cells in both the corpus luteum and ovaries [68]. Moreover, *INHBA* expression is detected in cumulus and mural granulosa cells within sinus follicles. Li et al. [69] proposed that *INHBA* displays varying expression levels across different follicular stages and exhibits particularly high expression during the granulosa cell growth phase in multiparous lamb goat populations. Notably, mutations in the *INHBA* gene have been found to significantly impact litter size in sheep. This further confirms its potential to influence the lambing performance of goats by regulating follicle development [67].

As bone quality is an essential trait in lactating cows, two significant SNPs (ARS-BFGL-NGS-37048, *p*-value = 1.49 × 10^−5^; Hapmap54735-ss46526095, *p*-value = 4.31 × 10^−6^) were detected on BTA4 and BTA16. These SNPs were positioned within genes including *EVX1*, *HOXA13*, and *VAMP4*. The transcription factor *HOXA13* is crucial for the morphogenesis and patterning of the vertebrate skeleton during embryonic development in animals [70]. Particularly, the *HOXA13* gene plays a pivotal role in numerous functions. It regulates the formation of digits, phalangeal joints, and carpal/tarsal elements during limb development [71,72]. Additionally, during organogenesis, it modulates the development of the digestive and urogenital tracts, including the differentiation of the mammalian female reproductive system [73]. Furthermore, there is compelling evidence indicating pleiotropic effects on *HOXA13*. Mutations in this gene simultaneously impact the development of both the urogenital system and the limbs [74]. Notably, in mice, a 50-base pair deletion at the first exon of *HOXA13* leads to hypodactyly [75]. Conversely, the expansion of an N-terminal polyalanine in this first exon is linked to abnormalities in both the limbs and genitourinary system in humans [74].

#### 3.2.4. Udder Traits

A total of six significant SNPs were identified for udder traits in the studied population (Table 2). Specifically, the traits of udder texture, fore udder attachment, rear udder height, and rear udder width exhibited associations with two, one, two, and one SNP, respectively. Remarkably, within the scope of this investigation, no SNPs meeting the criteria for significance (both under the Bonferroni correction and the suggestive significance threshold) were observed in relation to udder depth, udder support, front teat placement, front teat length, and rear teat placement in the context of Korean Holstein cattle. Within the intergenic variants of known genes, we identified five SNPs: BTB-01693574 (*p*-value = 1.96 × 10^−5^), BTB-01584048 (*p*-value = 7.57 × 10^−6^), ARS-BFGL-NGS-118699 (*p*-value = 1.96 × 10^−5^), Hapmap46979-BTA-32175 (*p*-value = 9.87 × 10^−6^), and BTA-11097-rs29016861 (*p*-value =1.52 × 10^−5^). Notably, another SNP, Hapmap29824-BTA-137304 (*p*-value = 7.76 × 10^−6^), was found downstream of known gene variants. Manhattan plots showing the genome-wide distribution of the significant SNPs underlying our selected udder traits are provided in Supplementary Material Figure S10. Additionally, we generated QQ plots to assess the extent to which observed *p*-values differ from expected *p*-values under the null hypothesis, assuming no association (Supplementary Material Figure S11). 

In relation to udder texture, a noteworthy significant SNP was identified within the intergenic variant of the *LOC104969871* gene on BTA2. Another SNP was also situated within the intergenic region of neighboring genes, including *MIR2285K-4* and *LOC788808* on BTA26. The function of the *MIR2285K-4* protein has not been extensively studied. For fore udder attachment, one significant SNP was found within the intergenic variants of *LOC511409*, *LOC104969247* gene located onBTA8. The functions of the nearby candidate genes identified within significant SNP regions in cattle are not well documented.

Two significant SNPs were identified on BTA13 and BTA28 associated with rear udder height in the Holstein population. Both SNPs were located within the intergenic variants of genes: *LOC104973698*, *LOC107133022*, *CDK1*, and *RHOBTB1*. Although the function of the *CDK1* gene has not been extensively studied, recent in vivo research has conclusively established that *CDK1* is both essential and sufficient for driving the resumption of meiosis during mouse oocyte maturation. Additionally, *CDK1* is implicated in the progression of the cell cycle in mammals [76]. Conversely, the *RHOBTB1* gene plays a crucial role in vascular smooth muscle cells, contributing to the regulation of vasomotor function and blood pressure [77]. A significant SNP was discovered in the regions downstream of the genes *SLC17A1* and *LRRC16*, both situated on BTA23, with relevance to fore udder attachment. In relation to this, Wubuli et al. [78] have reported that the expression of the *SLC17A1* gene might play a role in transporting uric acid from the liver to the serum and kidney in pigs. Notably, the expression of *SLC17A1* demonstrated a connection to the export of urate in the kidneys and the movement of phosphate (P) across hepatic basolateral membranes. Intriguingly, its expression exhibited a notably higher level in liver and lung tissues compared to its relatively lower expression in the kidney. This observation aligns with earlier research findings that also documented the expression of the *SLC17A1* gene across various tissues including the heart, muscles, kidneys, liver, and lungs. Furthermore, this gene is involved in multiple transport functions such as chloride-dependent anion transport, sodium-dependent phosphate uptake, and poly-specific organic anion transport, all of which contribute to the circulation of urate.

The incorporation of categorical data, specifically Holstein linear body conformation traits, into GRAMMAR-based GWAS [32] holds the potential to significantly advance our understanding of the genetic basis of these traits in Holstein cattle populations. This approach can provide finer trait resolution, improve the detection of rare variants, reduce phenotypic noise, enhance interpretability, enable multivariate analysis, and effectively address population stratification and relatedness issues. Ultimately, these advantages contribute to a more comprehensive and accurate exploration of the genetic architecture underlying Holstein body conformation traits, benefiting both scientific research and practical breeding programs in the dairy industry.

### 3.3. Functional Analysis

**Figure 1:**
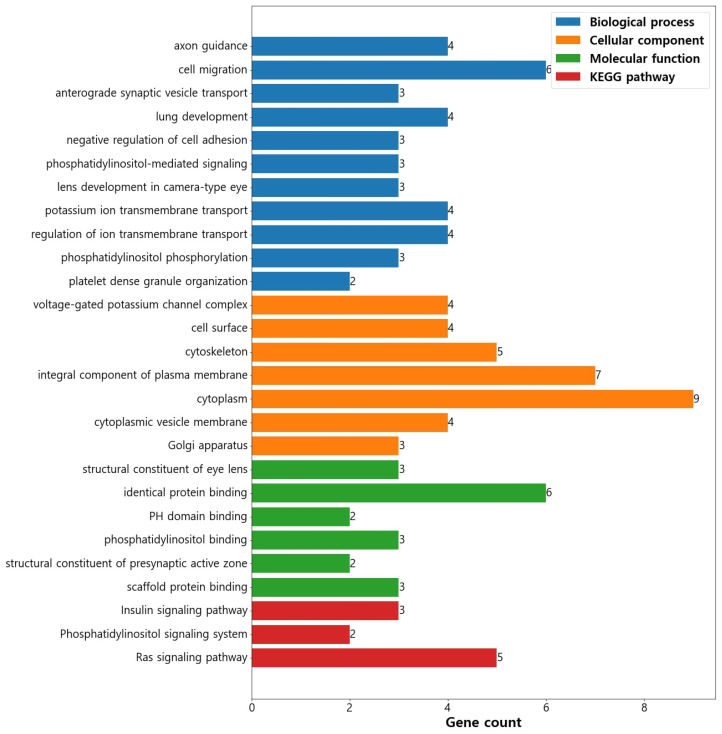
Significant GO terms (biological process, cellular component, and molecular function) and KEGG pathways of candidate genes associated with body conformation traits.

We conducted gene ontology and pathway enrichment analyses using the DAVID functional classification clustering tools to gain insights into the genes identified within quantitative trait loci (QTL) windows. The purpose of these analyses was to explore the functional significance of the genes related to the studied traits. Notably, a total of 37 gene ontology (GO) terms exhibited significant enrichment (*p* < 0.05) within the dataset (Supplementary Material Table S1). These enriched GO terms were categorized into distinct functional groups, comprising 16 terms associated with biological processes, 11 terms related to cellular components, and 10 terms concerning molecular functions (Figure 1). The study highlighted several biological process terms, such as axon guidance (GO:0007411), cell migration (GO:0016477), lung development (GO:0030324), negative regulation of cell adhesion (GO:0007162), phosphatidylinositol-mediated signaling (GO:0048015), potassium ion transmembrane transport (GO:0071805), regulation of ion transmembrane transport (GO:0034765), and phosphatidylinositol phosphorylation (GO:0046854). These terms were found to be the most significant factors influencing the traits investigated. Axon guidance plays a vital role in nervous system development, with a close relationship to brain-derived neurotrophic factor (BDNF) growth [79]. Moreover, BDNF development is closely intertwined with the process of axon guidance. Cell migration is essential for the development and maintenance of multicellular organisms, influencing various reproductive events such as embryo formation [80], spermatogenesis [81], immunological responses [82], and oogenesis [83]. Additionally, it affects cow conception rate, daughter pregnancy rate [84], nervous system development [85], perinatal mortality [86], and immunity [87]. Phosphatidylinositol-mediated signaling has significant regulatory roles, such as in human ovarian granulosa cells [88]. Potassium is crucial for supporting the reproduction of early-lactation dairy cows, particularly during heat stress [89]. Regulation of ion transmembrane transport is involved in signal transduction, intercellular communication, gas and nutrients exchange, embryogenesis, and growth [90]. Phosphatidylinositol plays a crucial role in various physiological functions, including muscle contraction, cell proliferation, and differentiation [91].

In terms of cellular component, the study found significance in voltage-gated potassium channel complex (GO:0008076), cell surface (GO:0009986), integral component of plasma membrane (GO:0005887), cytoplasm (GO:0005737), and cytoplasmic vesicle membrane (GO:0030659). Voltage-gated potassium channels facilitate ion transport across cell membranes, regulating cellular electrical activity and maintaining membrane potential [92]. The integral component of the plasma membrane gene set is essential for various cellular processes and their interactions with lipid bilayers and membrane-associated proteins [93].

Furthermore, significant enrichments were observed for molecular function terms, including structural constituent of eye lens (GO:0005212), identical protein binding (GO:0042802), and phosphotyrosine binding (GO:0001784). 

Furthermore, the KEGG pathways analysis revealed the enrichment of three specific pathways (*p* ≤ 0.05) within the dataset. These pathways include the insulin signaling pathway (bta04910), involving three genes; the phosphatidylinositol signaling system (bta04070), consisting of two genes; and the Ras signaling pathway (bta04014), with five genes (Table 3). The enrichment of these pathways indicates their potential involvement in the regulation of cellular processes and signaling cascades. For instance, the insulin signaling pathway’s enrichment suggests its crucial role in mediating cellular responses to insulin and impacting various metabolic processes. Additionally, the phosphatidylinositol signaling system’s enrichment implies its significance in cellular signaling events mediated by phosphatidylinositols. Lastly, the enrichment of the Ras signaling pathway points to its potential involvement in regulating cellular activities influenced by Ras proteins.

Within our studied Holstein population, certain candidate genes, such as *CCDC12* and *PTH1R*, showed influence on more than one trait, indicating a pleiotropic effect that impacts multiple traits simultaneously. For example, the *CCDC12* gene was associated with chest width and body depth. The results of the gene ontology enrichment analysis and the KEGG pathways analysis provide valuable evidence for the functional relevance and potential roles of specific biological processes and pathways in the dataset. These findings contribute to a deeper understanding of the genetic and molecular mechanisms governing the studied system, guiding further investigations into the functional relationships and interactions of the analyzed genes.

## 4. Conclusions

In this GWAS analysis, we performed an association study for conformation traits in Korean Holstein cattle. The current study is the first-ever attempt at a GWAS in Korean Holstein for body conformation traits according to the published reports. A GWAS with a larger sample size is considered as a potential tool to identify novel genomic regions underlying our traits of interest. It is important to note that conformation traits are likely controlled by multiple genes, each contributing a small proportion of the variation. Furthermore, mapping genes to SNP loci may not always result in locating the genes within or in close proximity to the identified SNPs. As there were no previous report of GWAS in Korean Holstein for conformation traits, the findings of the current study serve as a baseline and would certainly require further research to be validated. Other concerns might be raised as to the effect of breed composition and population structure on the results of the current GWAS analysis. The present GWAS for body conformation traits in Korean Holstein permitted the identification of chromosomal regions, contributing to a better understanding of the genetic and physiologic mechanisms regulating the traits, and identified candidate genes for the investigation of causal mutations. Remarkably, we have identified *KCNA1*, *RYBP*, *PTH1R*, *TMIE*, *GNAI3*, *ANGPT1*, *MALRD1*, *INHBA*, *HOXA13*, *CDK1*, *RHOBTB1*, and *SLC17A1* as the most promising candidate genes for stature, height at the front end, chest width, body depth, angularity, rump width, rear leg rear view, heel depth, bone quality, rear udder height, and rear udder width, respectively. These findings hold tremendous potential for future marker-assisted selection in Holstein cattle breeding programs, offering a pathway towards improved trait selection and genetic enhancement in this valuable breed. In conclusion, our pioneering GWAS analysis has illuminated the path forward in unlocking the genetic secrets behind Korean Holstein cattle’s conformation traits, paving the way for innovative advancements in cattle breeding practices.

## Figures and Tables

**Table 1:** Summary statistics for body conformation traits in the Korean Holstein population.

Traits	Mean	SD	CV (%)	Min	Max
Body traits					
Stature	6.81	1.28	18.84	1	9
Height at front end	4.88	0.60	12.23	3	8
Chest width	4.39	1.00	22.80	1	7
Body depth	4.75	1.00	21.21	1	8
Angularity	5.17	1.05	20.22	2	8
Body condition score	5.17	0.99	19.25	1	8
Locomotion	5.84	1.55	26.55	2	9
Rump traits					
Rump angle	4.75	1.05	22.14	1	9
Rump width	4.58	1.06	23.28	1	8
Loin strength	5.53	1.08	19.56	1	9
Feet and leg traits					
Rear leg set	5.01	0.99	19.73	2	9
Rear leg rear view	5.62	1.41	25.13	2	9
Foot angle	5.15	0.96	18.67	2	9
Heel depth	5.43	1.05	19.41	1	9
Bone quality	5.60	1.01	18.08	3	9
Udder traits					
Udder depth	6.40	1.16	18.18	3	9
Udder texture	5.44	1.36	24.99	2	9
Udder support	5.79	1.16	20.08	2	9
Fore udder attachment	5.67	1.17	20.67	1	9
Front teat placement	4.94	0.93	18.86	2	8
Front teat length	4.30	0.99	23.17	1	8
Rear udder height	6.88	1.18	17.22	1	9
Rear udder width	4.70	1.51	32.18	1	9
Rear teat placement	6.43	0.98	15.27	3	9

SD, standard deviation; CV, coefficient of variation; Min, minimum; Max, maximum.

**Table 2:** Genome-wide significant SNPs (*p* ≥ 2.58 × 10^−5^) associated with body conformation traits and nearest candidate genes in Korean Holstein population.

SNP	BTA:Position (bp)	*p*-Value	−log_10_P	Nearest Gene	$\sigma^{2}\left(\mathrm{SNP}\right)/\sigma^{2}\left(\mathrm{trait}\right)$
Stature					
Hapmap60480-ss46526970	5:105,870,613	1.18 × 10^−5^	4.930	*NDUFA9*, *KCNA1*, *KCNA6*	0.018
Hapmap50195-BTA-105299	11:76,615,660	6.34 × 10^−6^	5.198	*LOC107132946*	0.023
Height at front end					
ARS-BFGL-NGS-20317	22:29,854,903	7.90 × 10^−6^	5.103	*RYBP*	0.022
Chest width					
BTB-00853109	22:53,158,683	1.52 × 10^−5^	4.819	*CCDC12*, *PTH1R*	0.022
Body depth					
BTB-00853109	22:53,158,683	1.99 × 10^−5^	4.701	*CCDC12*, *PTH1R*	0.022
Hapmap43881-BTA-54837	22:53,309,647	2.02 × 10^−5^	4.695	*PRSS45*, *PRSS46*	0.022
ARS-BFGL-NGS-117960	22:53,348,693	3.20 × 10^−6^	5.495	*TMIE*	0.027
Angularity					
BTA-67308-no-rs	3:33,975,389	6.32 × 10^−6^	5.199	*GNAI3*	0.024
Hapmap51025-BTA-67309	3:33,997,602	6.32 × 10^−6^	5.199	*GNAI3*	0.024
ARS-BFGL-NGS-5218	10:9,208,908	2.31 × 10^−5^	4.637	*AP3B1*	0.019
Rump width					
BTB-00752634	14:59,049,305	3.16 × 10^−6^	5.500	*LOC104974091*, *LOC614209*	0.022
BTB-00574588	14:59,077,923	5.65 × 10^−6^	5.248	*LOC104974091*, *LOC614209*	0.021
ARS-BFGL-BAC-26802	14:59,502,755	2.06 × 10^−5^	4.686	*ANGPT1*, *LOC782496*	0.019
ARS-BFGL-NGS-5369	17:71,841,734	8.45 × 10^−6^	5.073	*OSBP2*	0.022
Rear leg rear view					
ARS-BFGL-NGS-629	13:21,442,240	1.05 × 10^−5^	4.978	MALRD1	0.020
Heel depth					
BTB-01928726	4:80,280,325	2.08 × 10^−5^	4.682	*INHBA*, *LOC104972162*	0.020
Bone quality					
ARS-BFGL-NGS-37048	4:69,262,899	1.49 × 10^−5^	4.826	*EVX1*, *HOXA13*	0.025
Hapmap54735-ss46526095	16:40,001,986	4.31 × 10^−6^	5.365	*VAMP4*	0.026
Udder texture					
BTB-01693574	2:23,061,078	1.96 × 10^−5^	4.709	*LOC104969871*	0.020
BTB-01584048	26:29,529,492	7.57 × 10^−6^	5.121	*MIR2285K-4*, *LOC788808*	0.020
Fore udder attachment					
ARS-BFGL-NGS-118699	8:2,641,745	1.96 × 10^−5^	4.708	*LOC511409*, *LOC104969247*	0.022
Rear udder height					
Hapmap46979-BTA-32175	13:5,795,316	9.87 × 10^−6^	5.006	*LOC104973698, LOC107133022*	0.024
BTA-11097-rs29016861	28:16,641,022	1.52 × 10^−5^	4.819	*CDK1*, *RHOBTB1*	0.022
Rear udder width					
Hapmap29824-BTA-137304	23:32,110,882	7.76 × 10^−6^	5.110	*SLC17A1*, *LRRC16A,*	0.021

SNP, single-nucleotide polymorphism; BTA, *Bos taurus* autosome; bp, base pair (kb); $\frac{\sigma^{2}\left(\mathrm{SNP}\right)}{\sigma^{2}\left(\mathrm{trait}\right)}$, proportion of phenotypic variance due to the SNP.

**Table 3:** KEGG pathways significantly enriched (*p* < 0.05) using candidate genes associated with the body conformation traits.

Term ID	Term Name	Gene Count	*p*-Value	Genes
bta04910	Insulin signaling pathway	3	0.00107	*RYBP*, *OSBP2*, *MALRD1*
bta04070	Phosphatidylinositol signaling system	2	0.01310	*TMIE*, *CDK1*
bta04014	Ras signaling pathway	5	0.03288	*CCDC12*, *LOC614209*, *OSBP2*, *EVX1*, *SLC17A1*

## Data Availability

The dataset is available from the corresponding author on reasonable request.

## Supplementary Materials

The following are available online at https://www.mdpi.com/article/10.3390/ani13182964/s1, Figure S1: Phenotypic distribution of 24 body conformation traits of Korean Holstein, Figure S2: Two-dimensional (C1 and C2) MDS plot based on data from Korean Holstein population, Figure S3: QQ, histogram, and density plots (a-x) of residuals used in GWAS analysis for 24 body conformation traits in Korean Holstein. Figure S4: Manhattan plots of genome-wide −log10 (*p*-values) for body traits in Holstein cattle, Figure S5: Quantile-quantile (Q-Q) plots and genomic inflation factor (λ) of the GWAS analysis for body traits in Korean Holstein, Figure S6: Manhattan plots of genome-wide −log10 (*p*-values) for rump traits in Holstein cattle, Figure S7: Quantile-quantile (Q-Q) plots and genomic inflation factor (λ) of the GWAS analysis for rump traits in Korean Holstein, Figure S8: Manhattan plots of genome-wide −log10 (*p*-values) for feet and leg traits in Holstein cattle, Figure S9: Quantile-quantile (Q-Q) plots and genomic inflation factor (λ) of the GWAS analysis for feet and leg traits in Korean Holstein, Figure S10: Manhattan plots of genome-wide −log10 (*p*-values) for udder traits in Holstein cattle, Figure S11: Quantile-quantile (Q-Q) plots and genomic inflation factor (λ) of the GWAS analysis for udder traits in Korean Holstein. Table S1: GO terms from DAVID v2023q2 software significantly enriched using candidate genes associated with the body conformation traits.
