# Supplementary material for: Genome-Wide Association Studies for Body Conformation Traits in Korean Holstein Population
Source: Animals (Basel). 2023 Sep 19;13(18):2964. doi: 10.3390/ani13182964 (PMC10526087; doi:10.3390/ani13182964)
Supplement: Supplementary file 1 [file animals-13-02964-s001.zip › animals-2553327-supplementary.pdf]

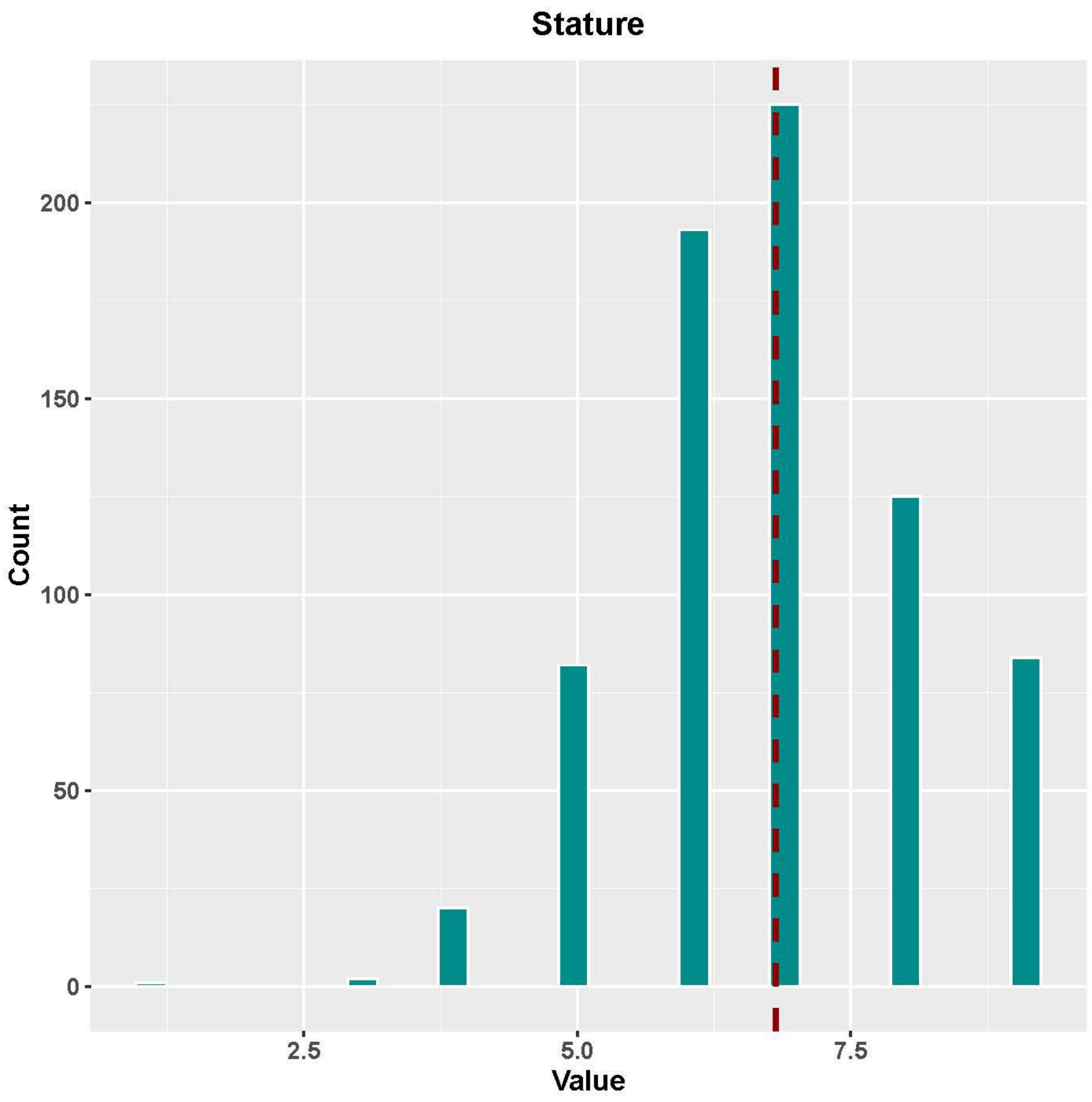

Stature
Count
200
150
100
50
0
2.5
5.0
7.5
Value

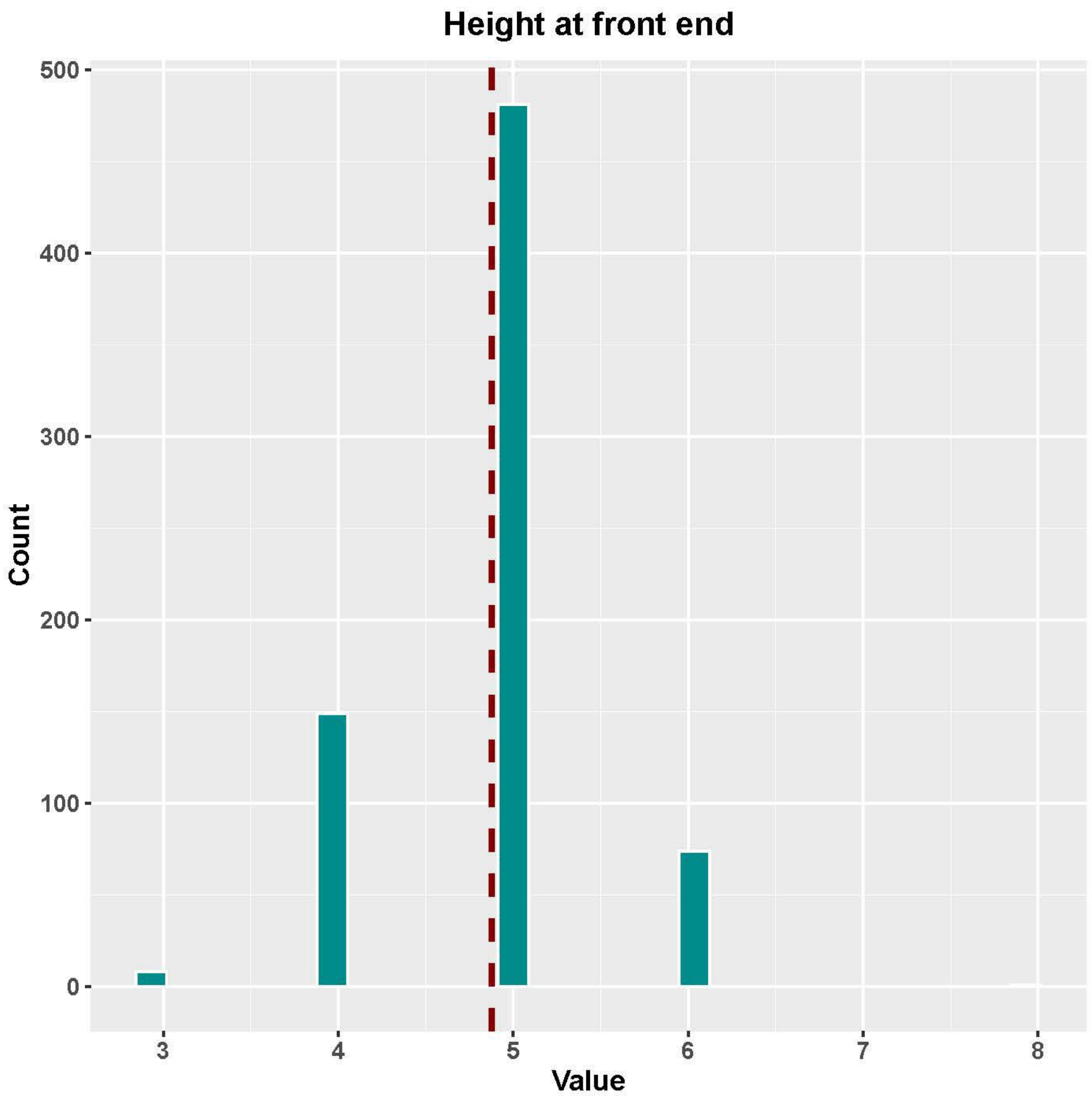

Height at front end
Count
500
400
300
200
100
0
3
4
5
6
7
8
Value

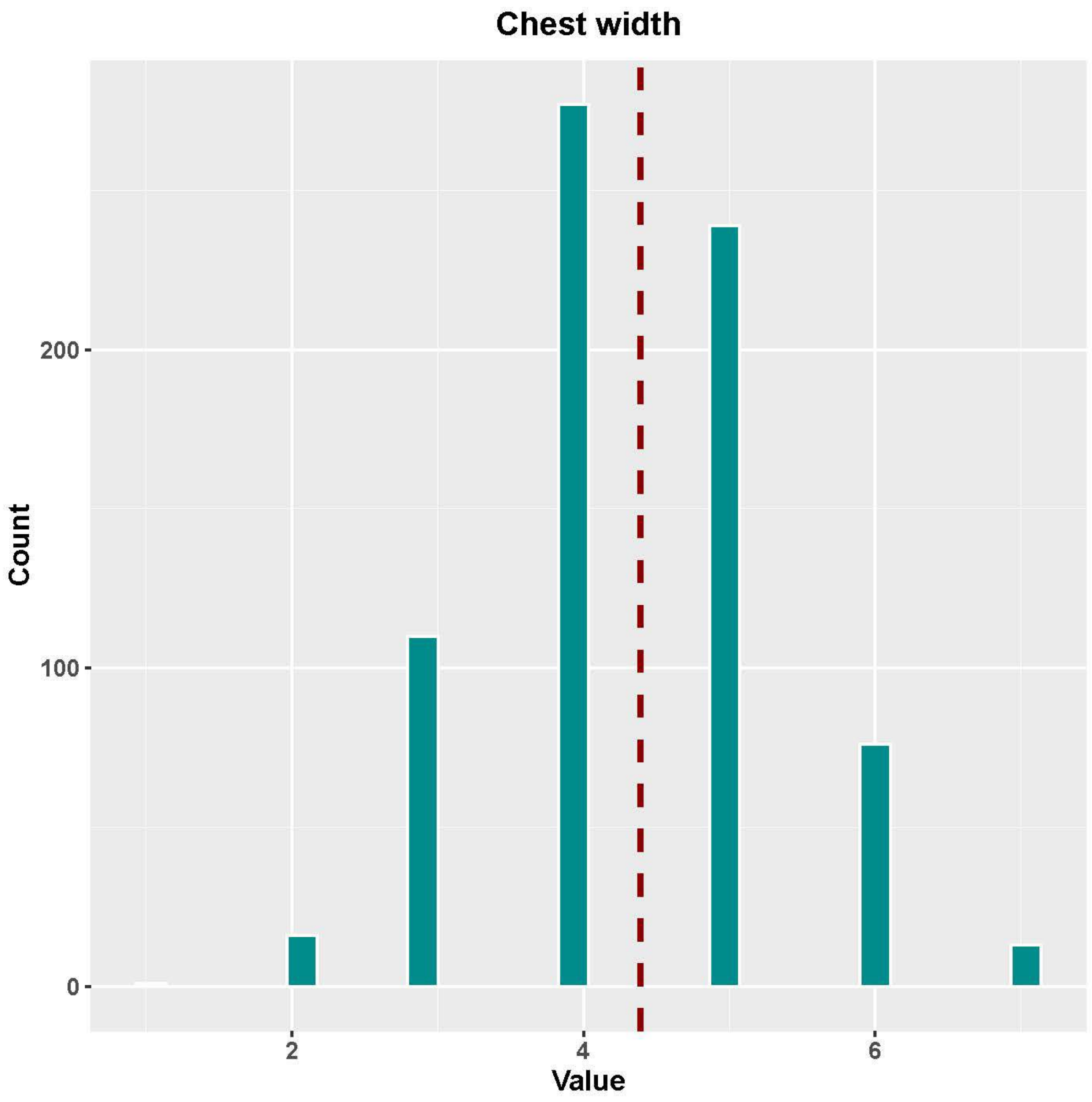
Chest width
Count
200
100
0
2
4
6
Value

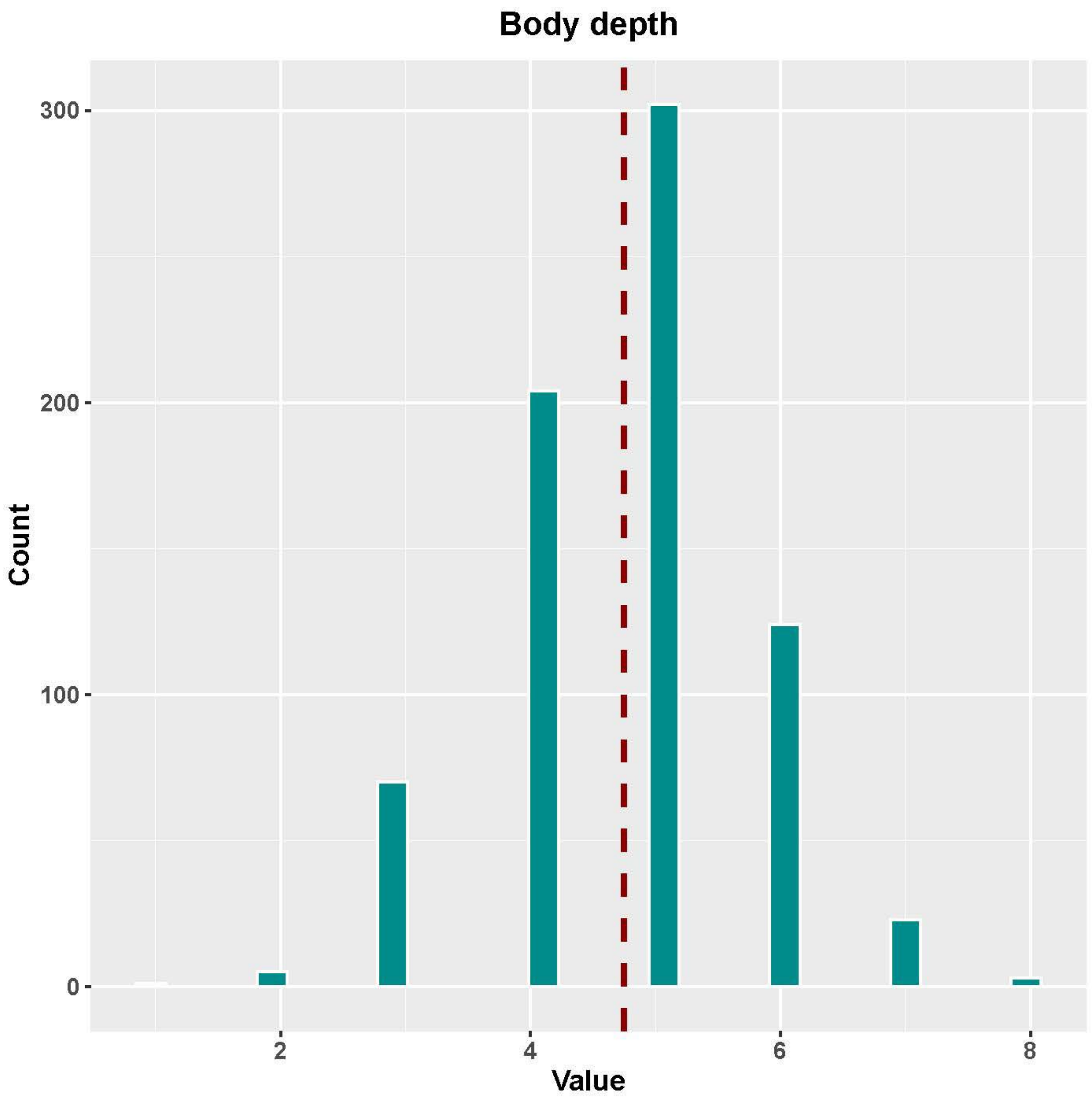

Body depth
Count
Value
300
200
100
0
2
4
6
8

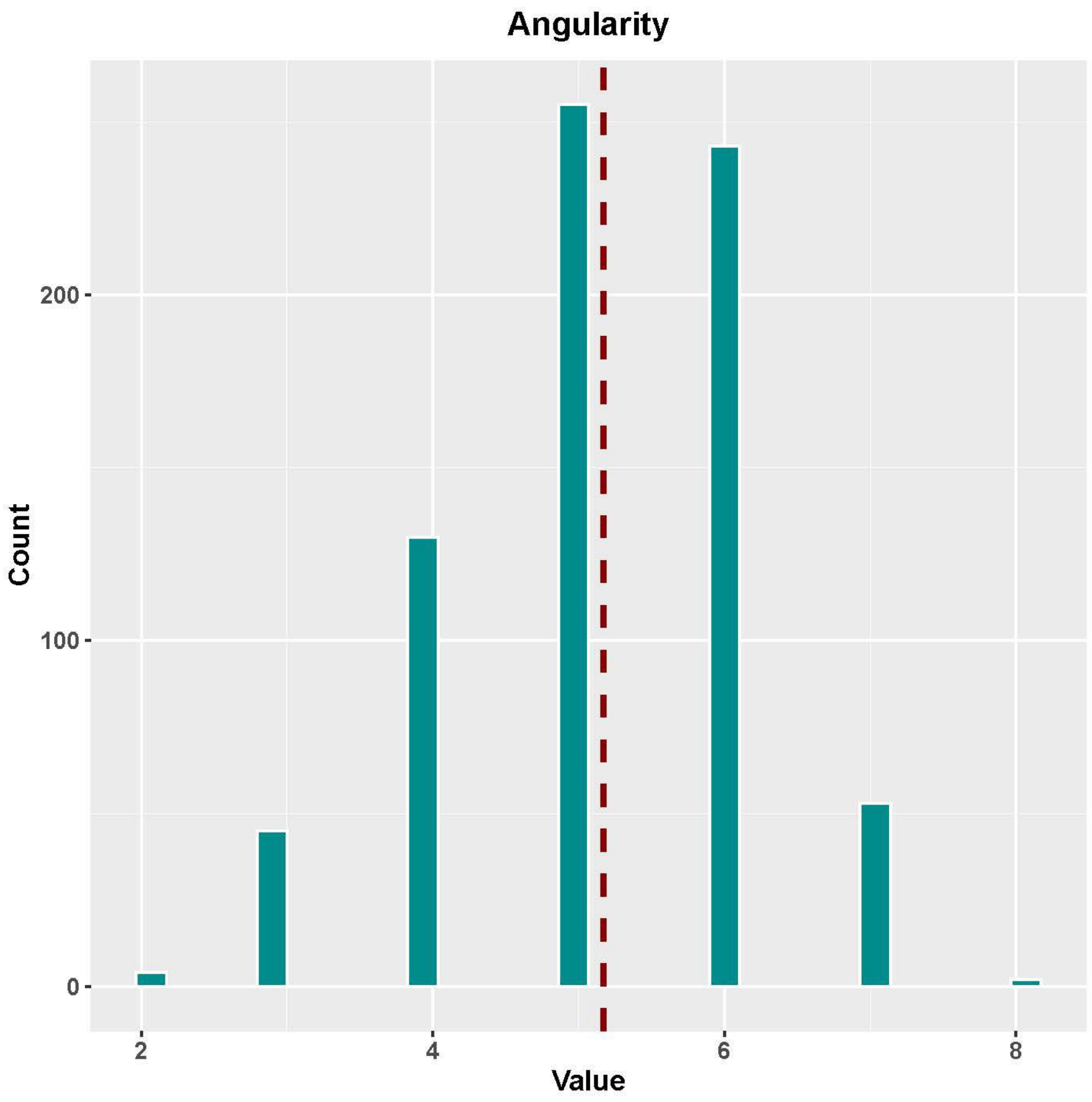
Angularity
Count
200
100
0
2
4
6
8
Value

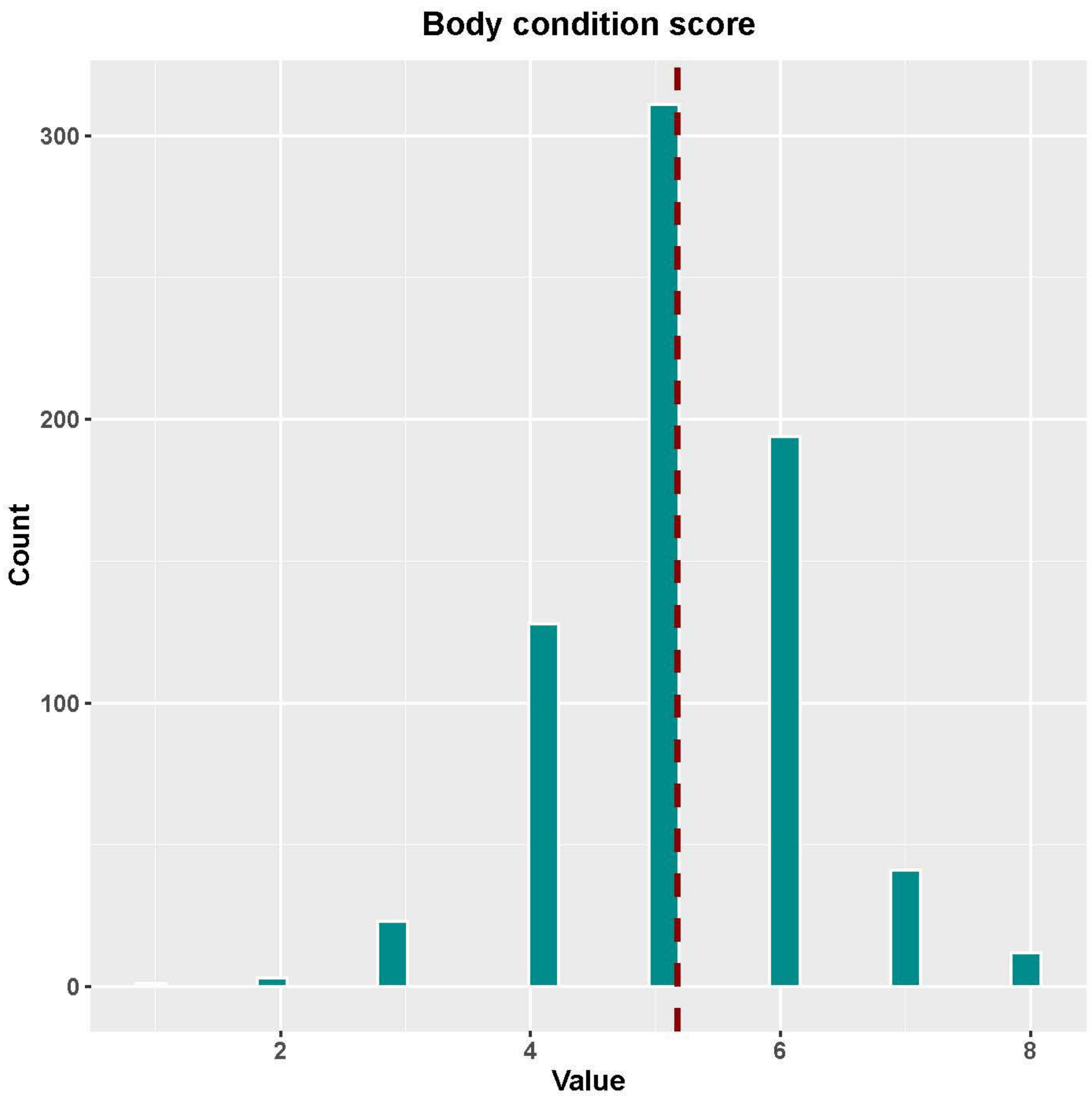
Body condition score
Count
300
200
100
0
2
4
6
8
Value

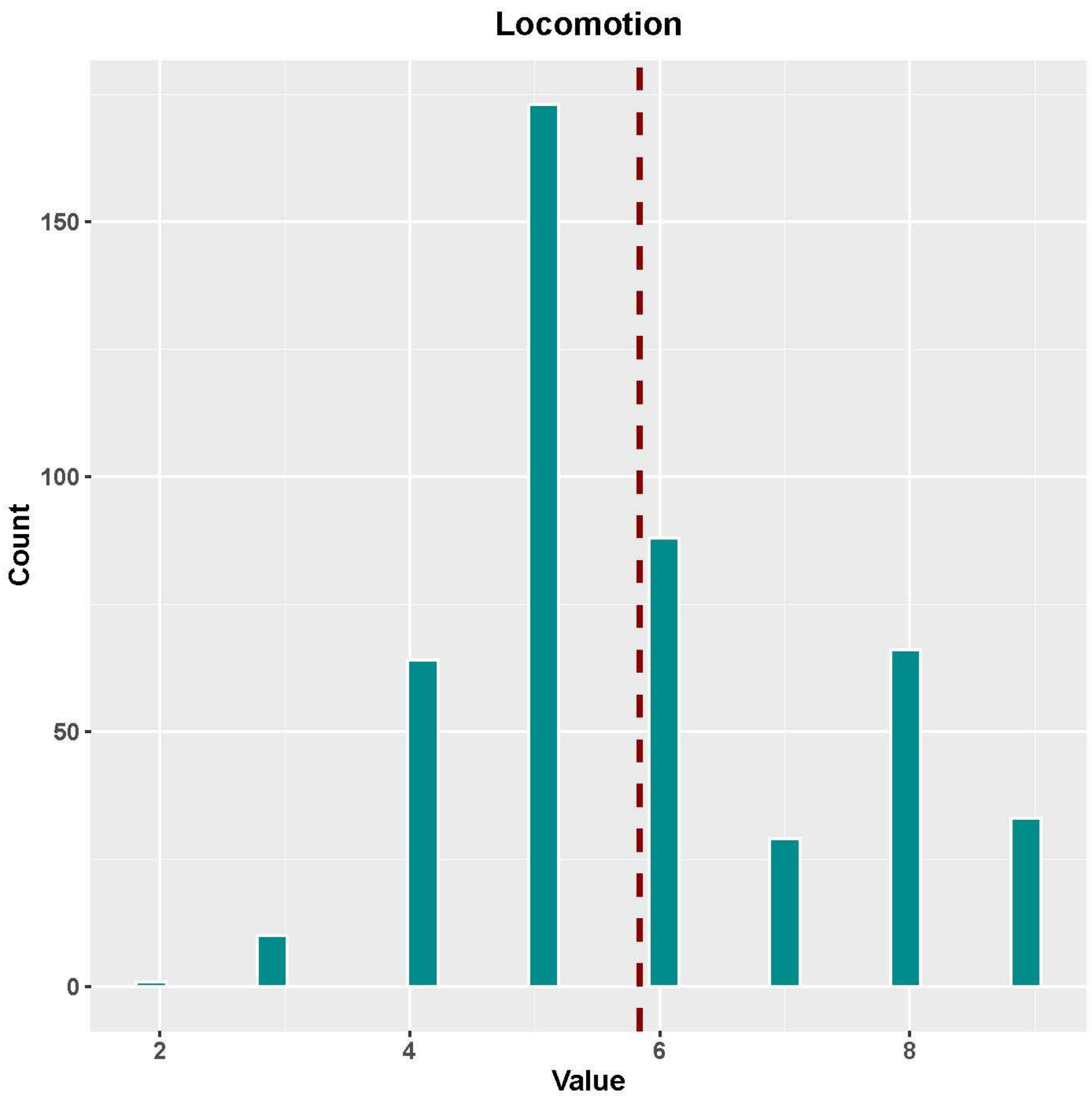
Locomotion
Count
150
100
50
0
2
4
6
8
Value

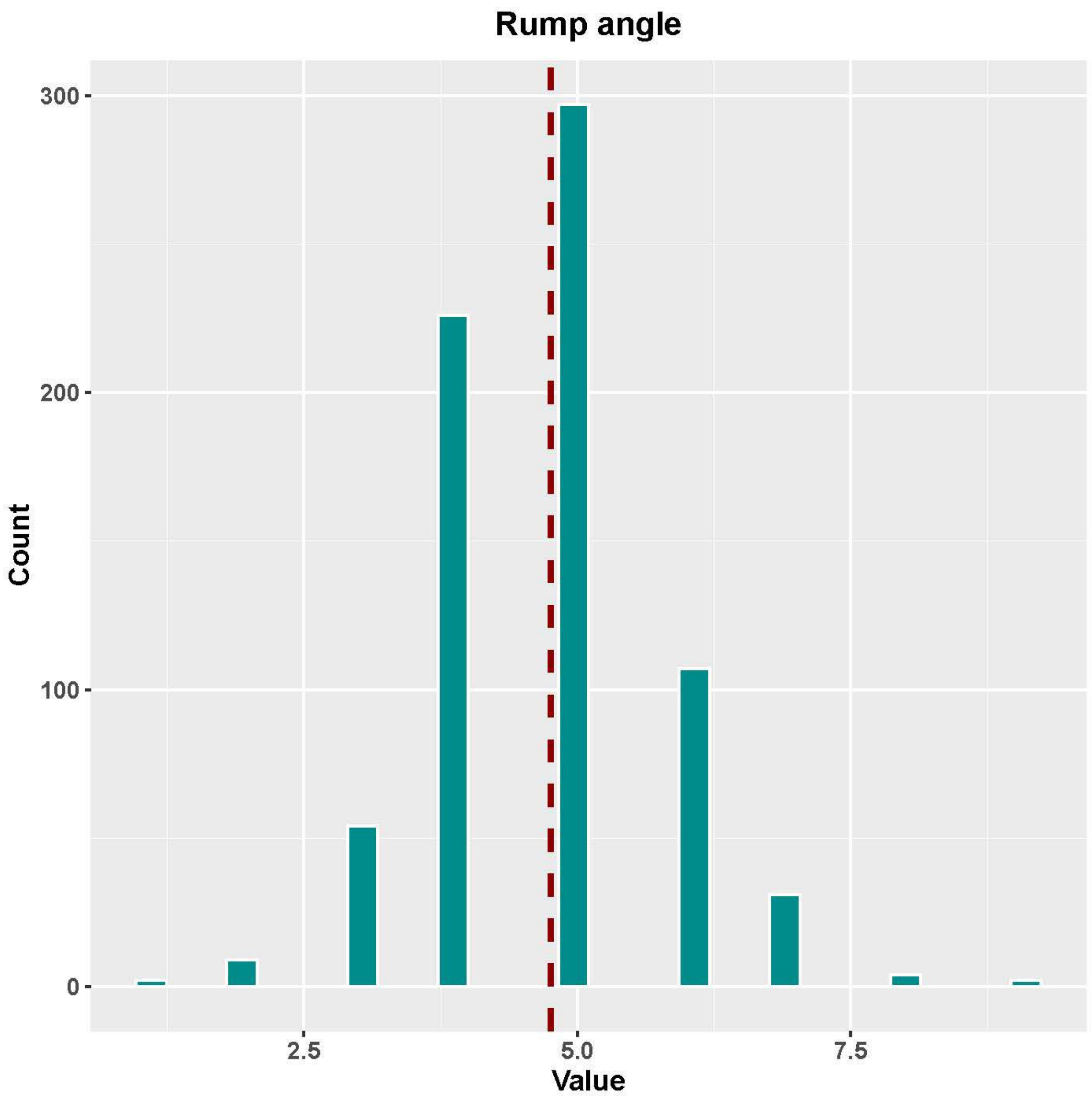
Rump angle
300
200
100
0
Count
2.5
5.0
7.5
Value

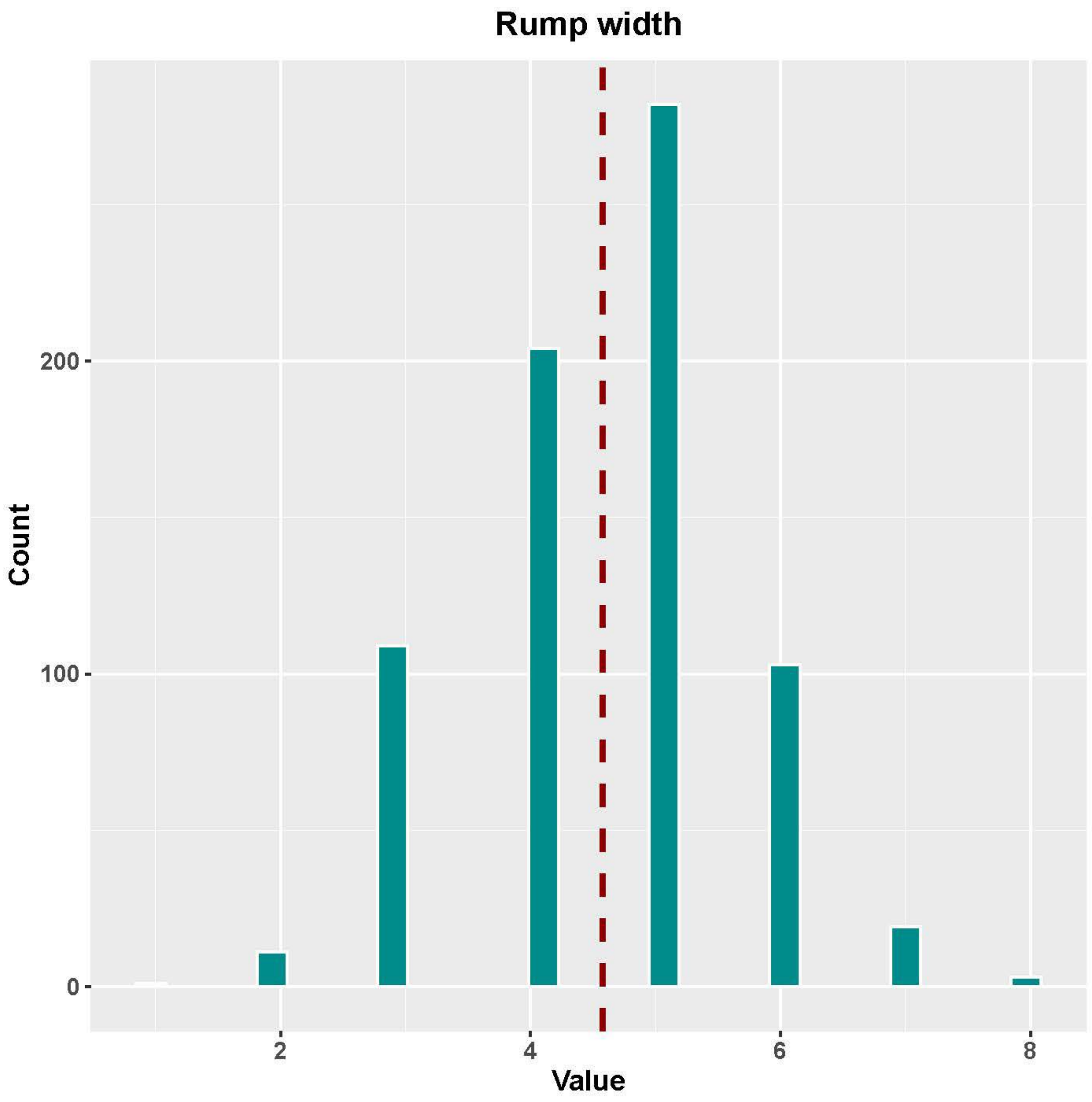

Rump width
Count
200
100
0
2
4
6
8
Value

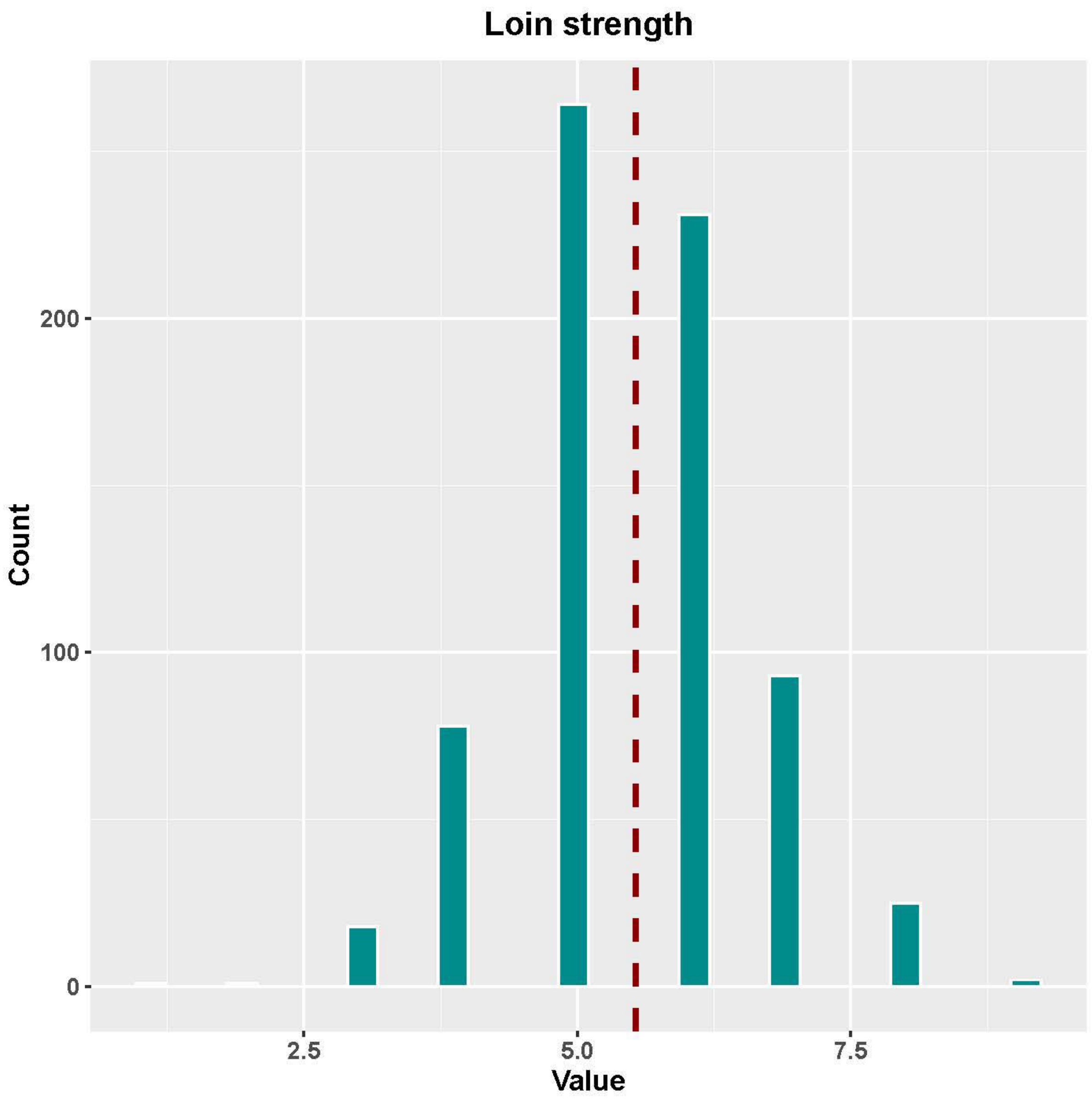

Loin strength
Count
200
100
0
2.5
5.0
7.5
Value

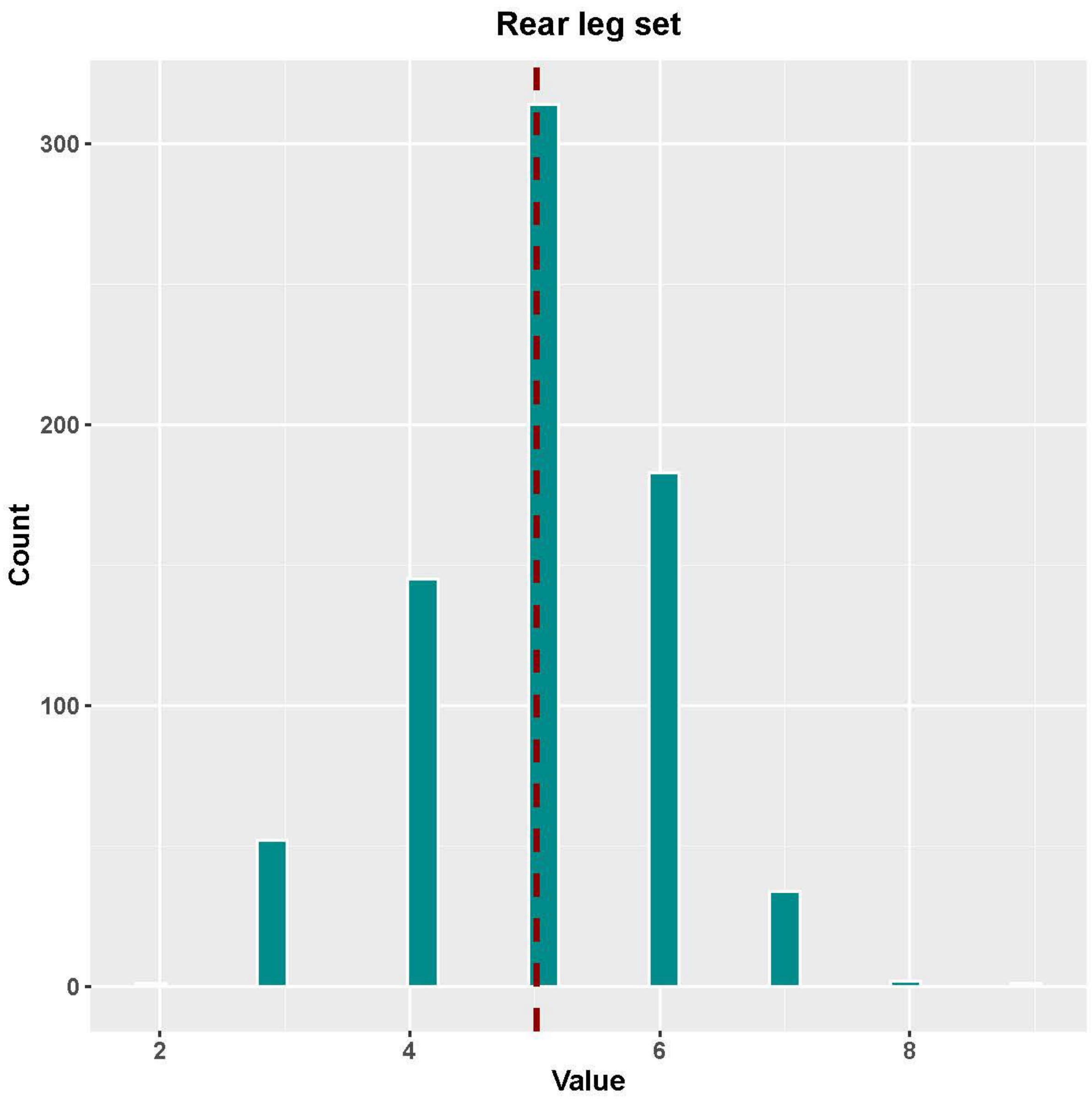
Rear leg set
300
200
100
0
Count
2
4
6
8
Value

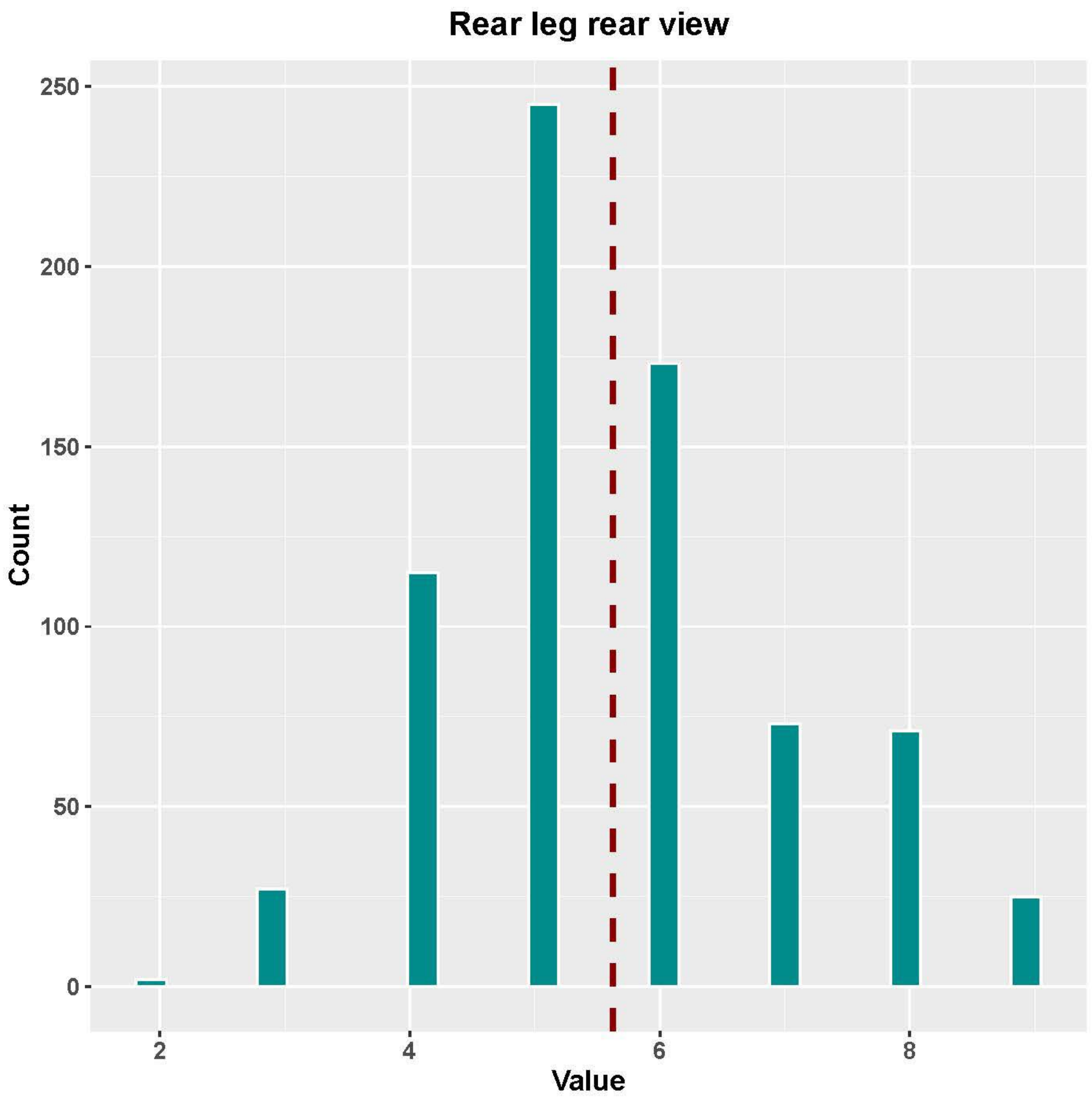

Rear leg rear view
Count
250
200
150
100
50
0
2
4
6
8
Value

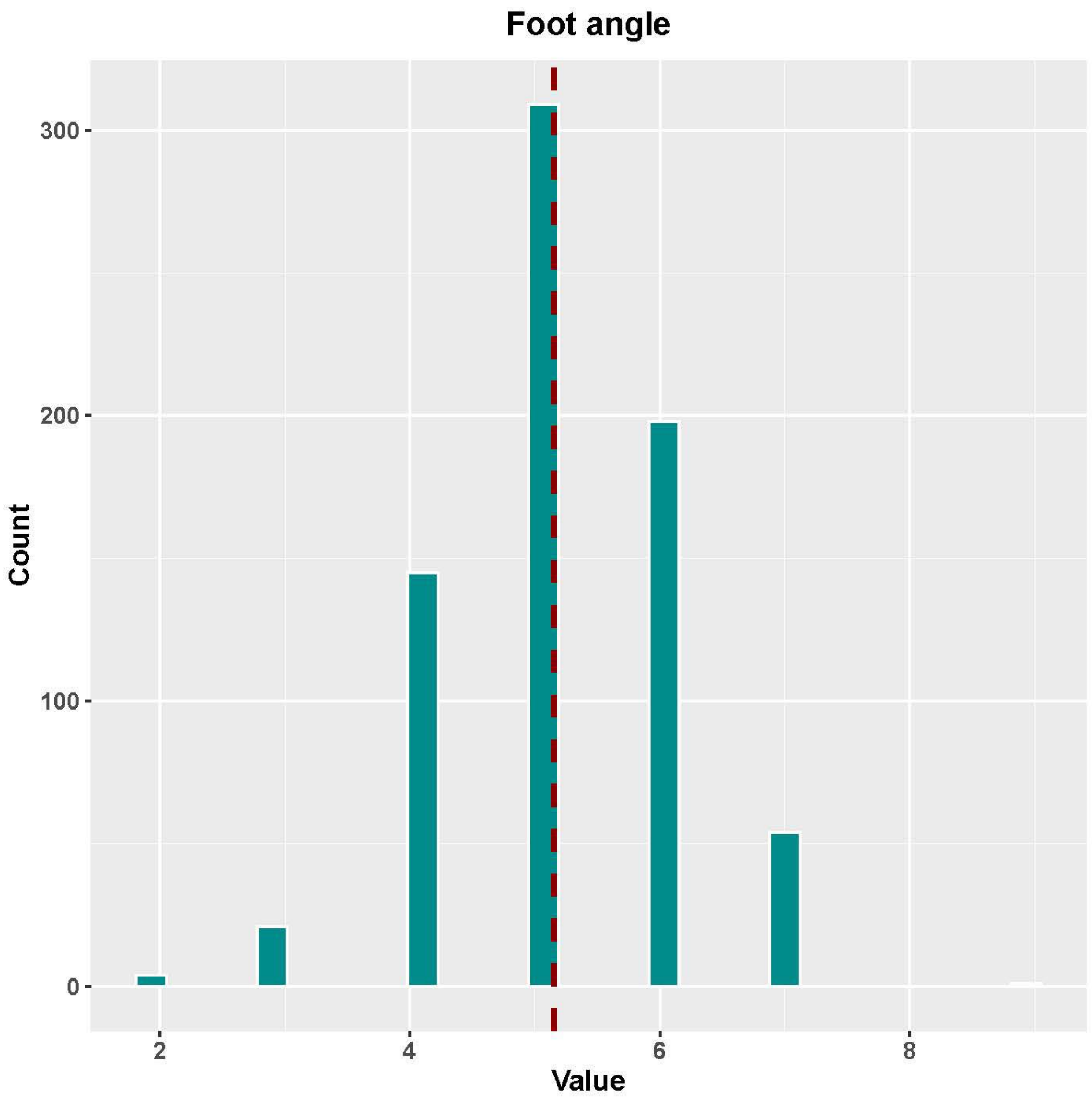

Foot angle
Count
Value
0
100
200
300
2
4
6
8

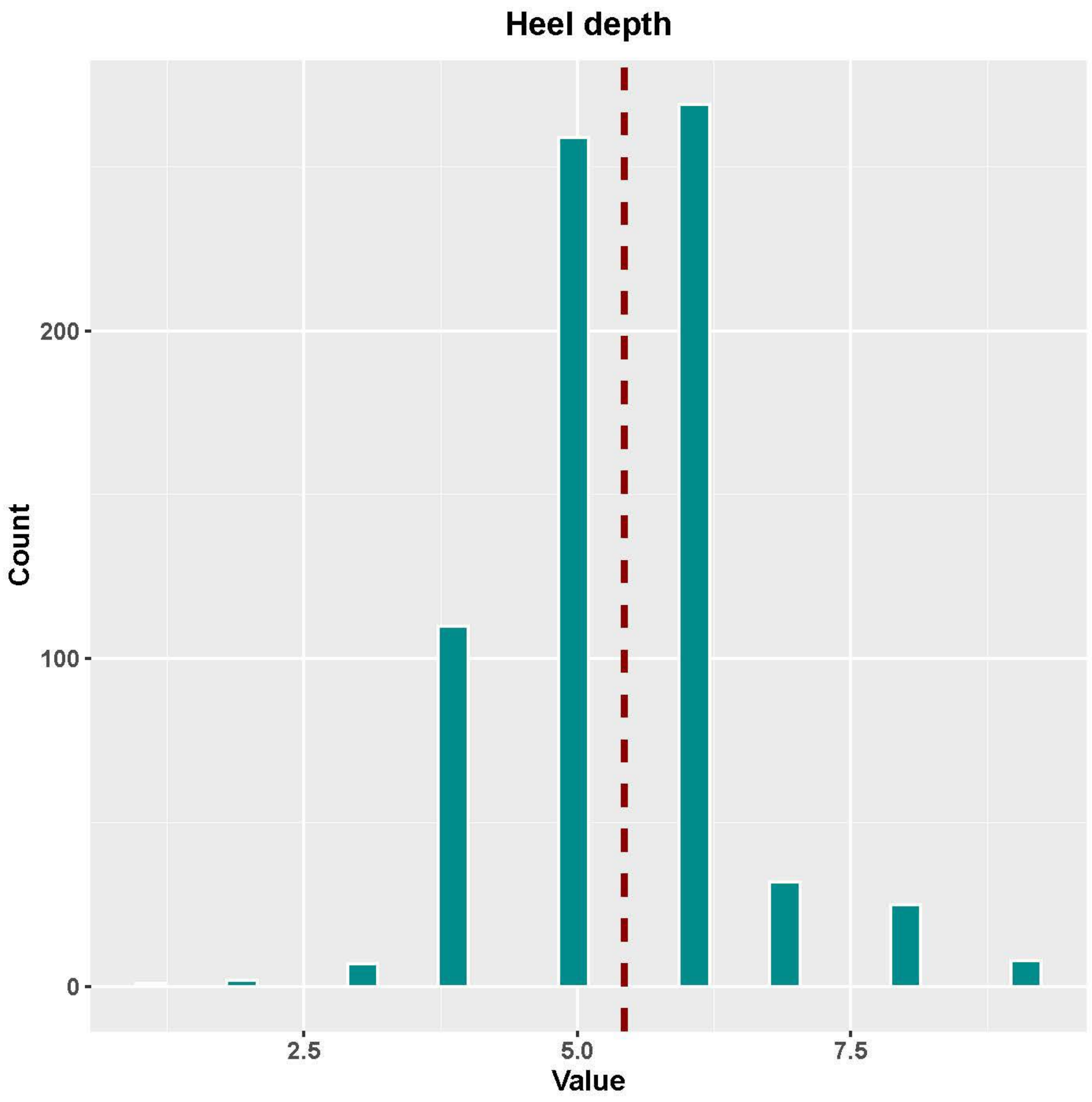
Heel depth
Count
200
100
0
2.5
5.0
7.5
Value

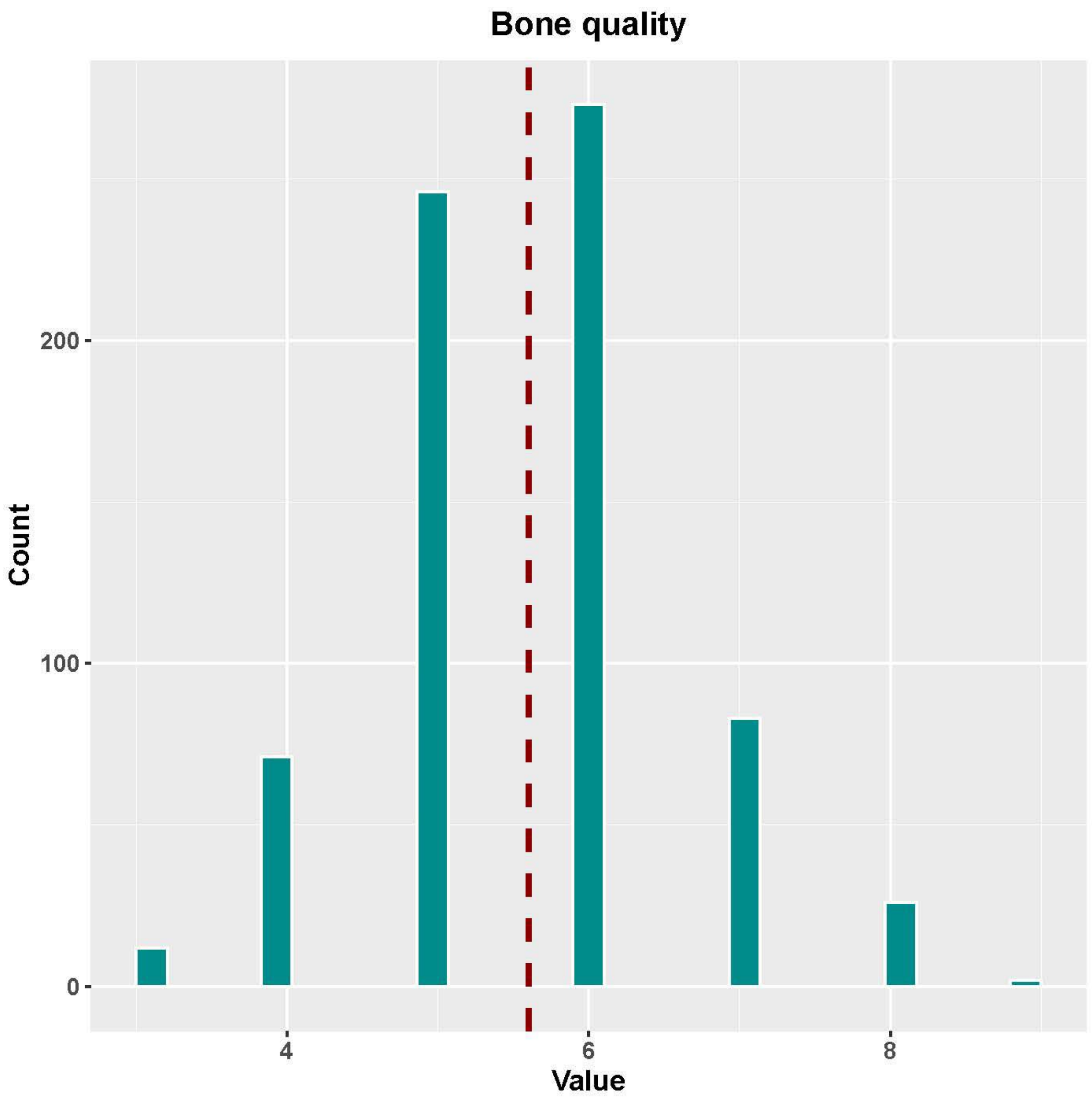

Bone quality
Count
Value
0
100
200
4
6
8

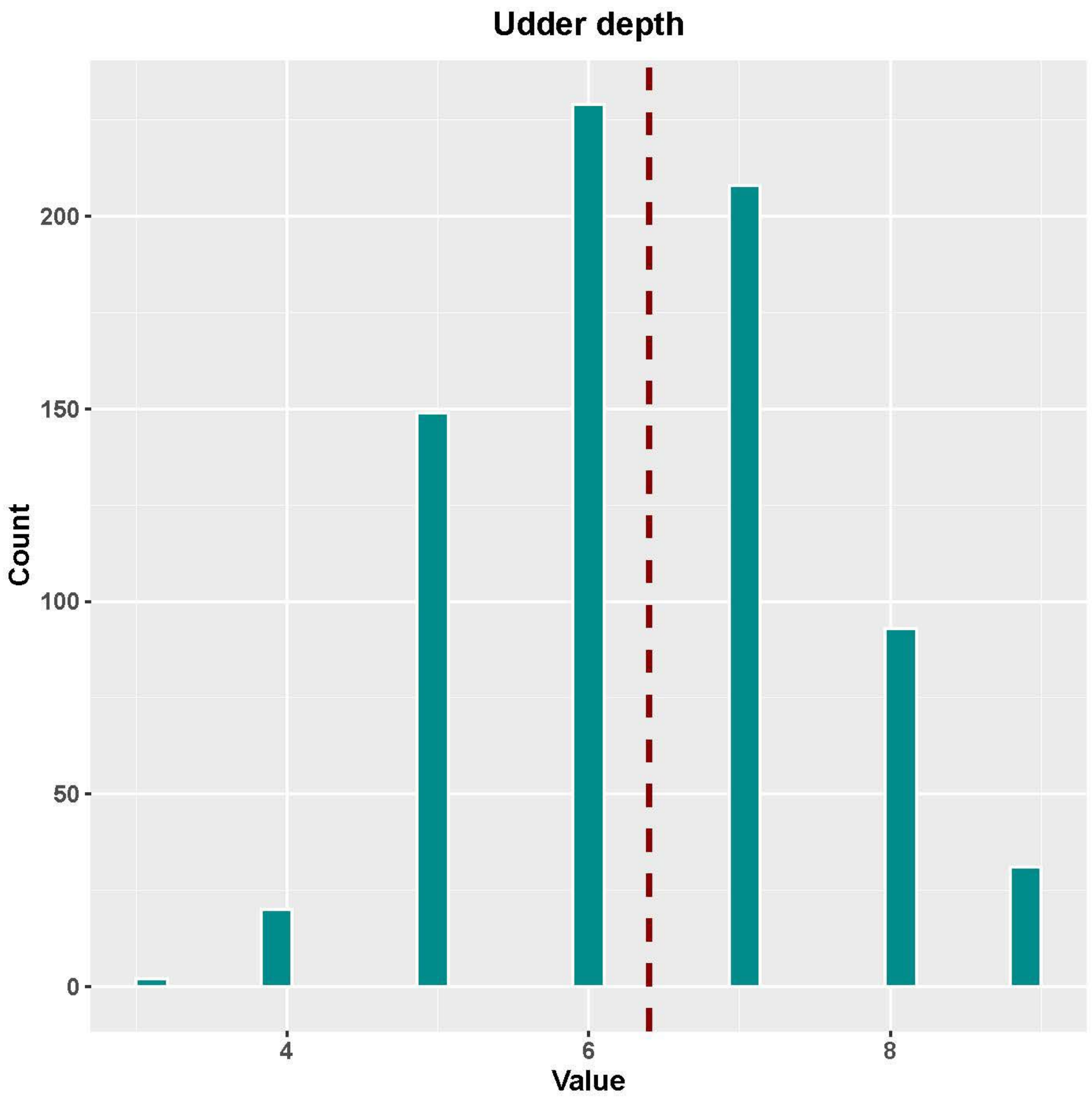

Udder depth
Count
Value
0
50
100
150
200
4
6
8

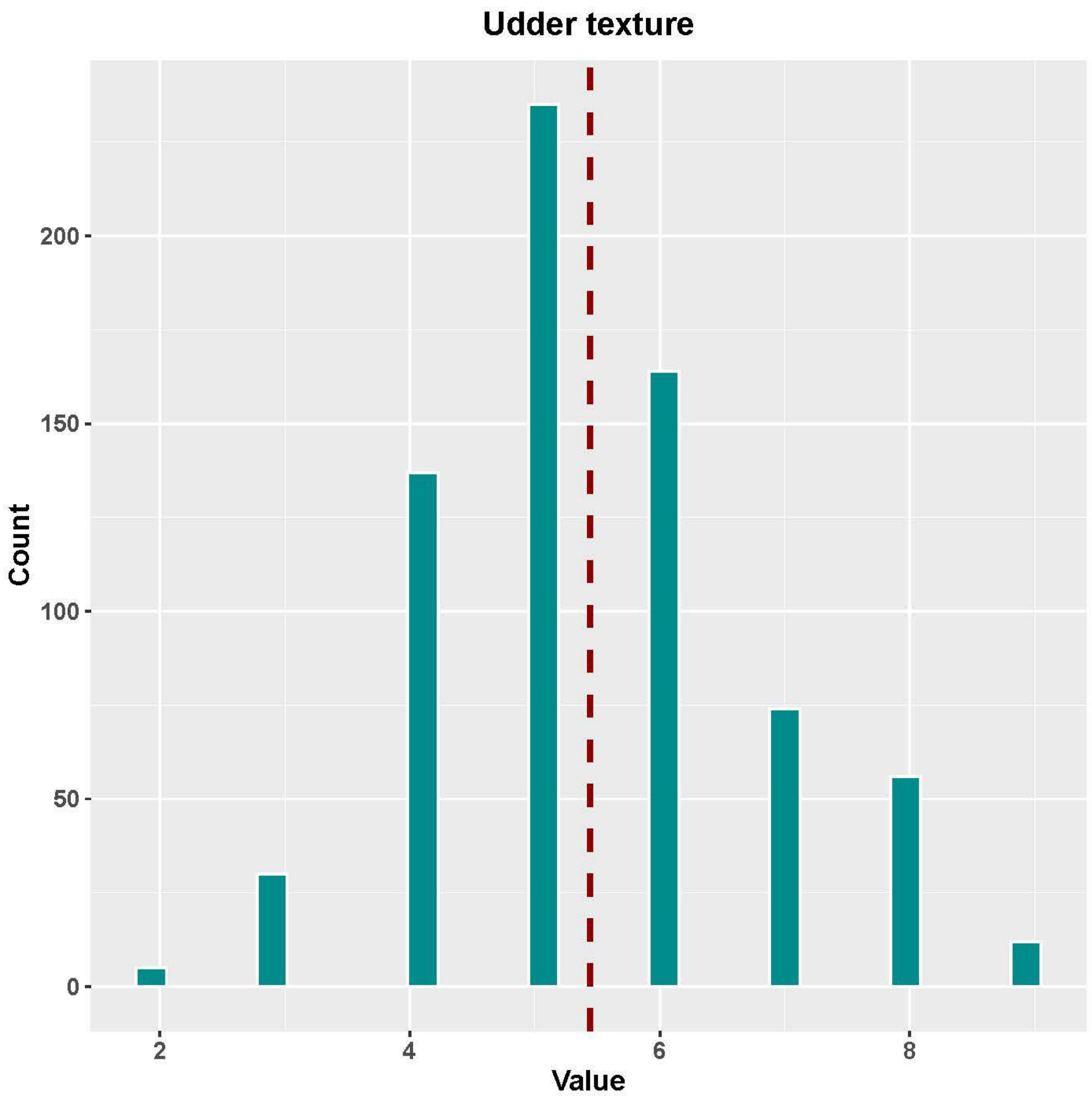
Udder texture
Count
200
150
100
50
0
2
4
6
8
Value

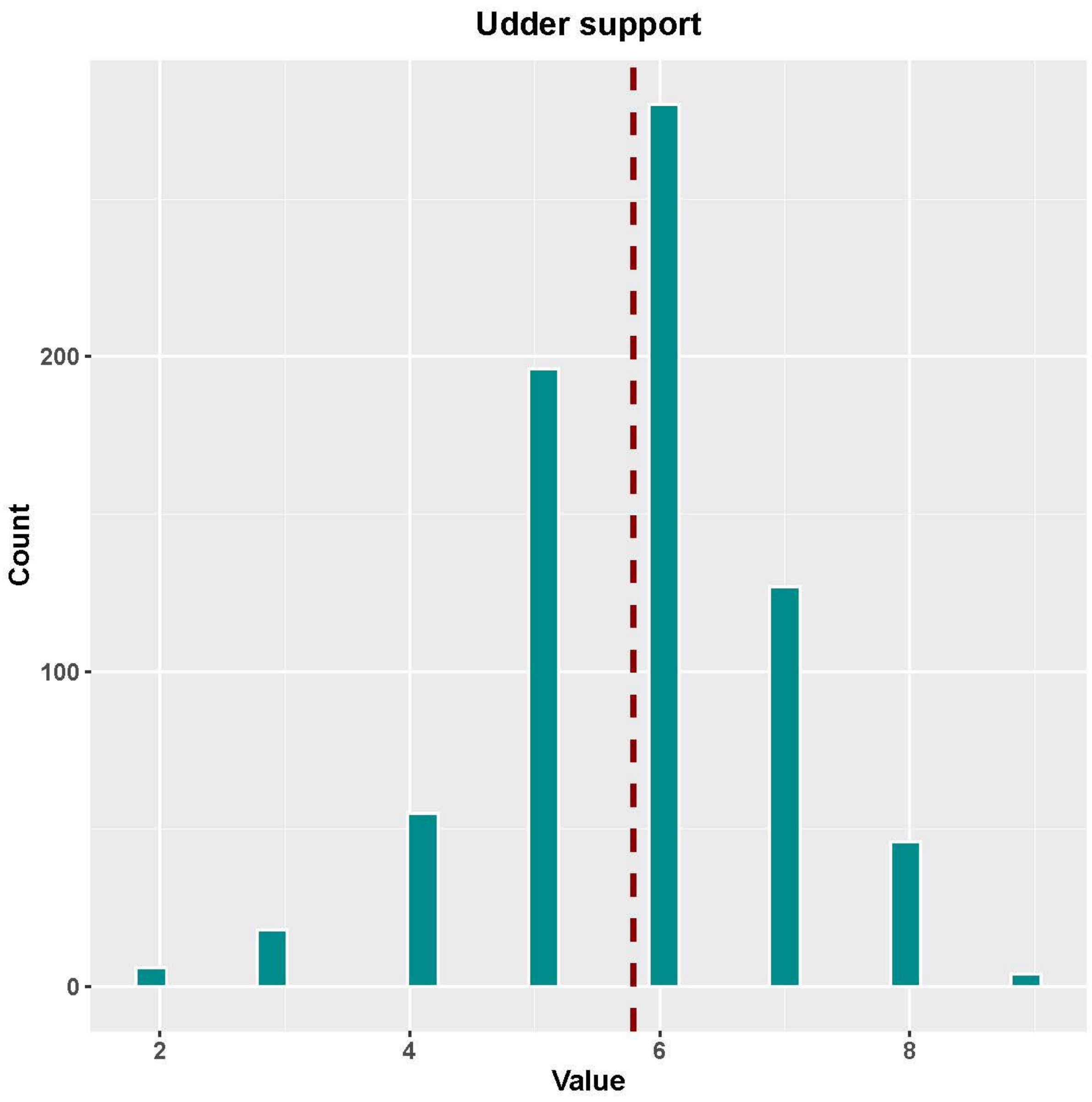

Udder support
Count
200
100
0
2
4
6
8
Value

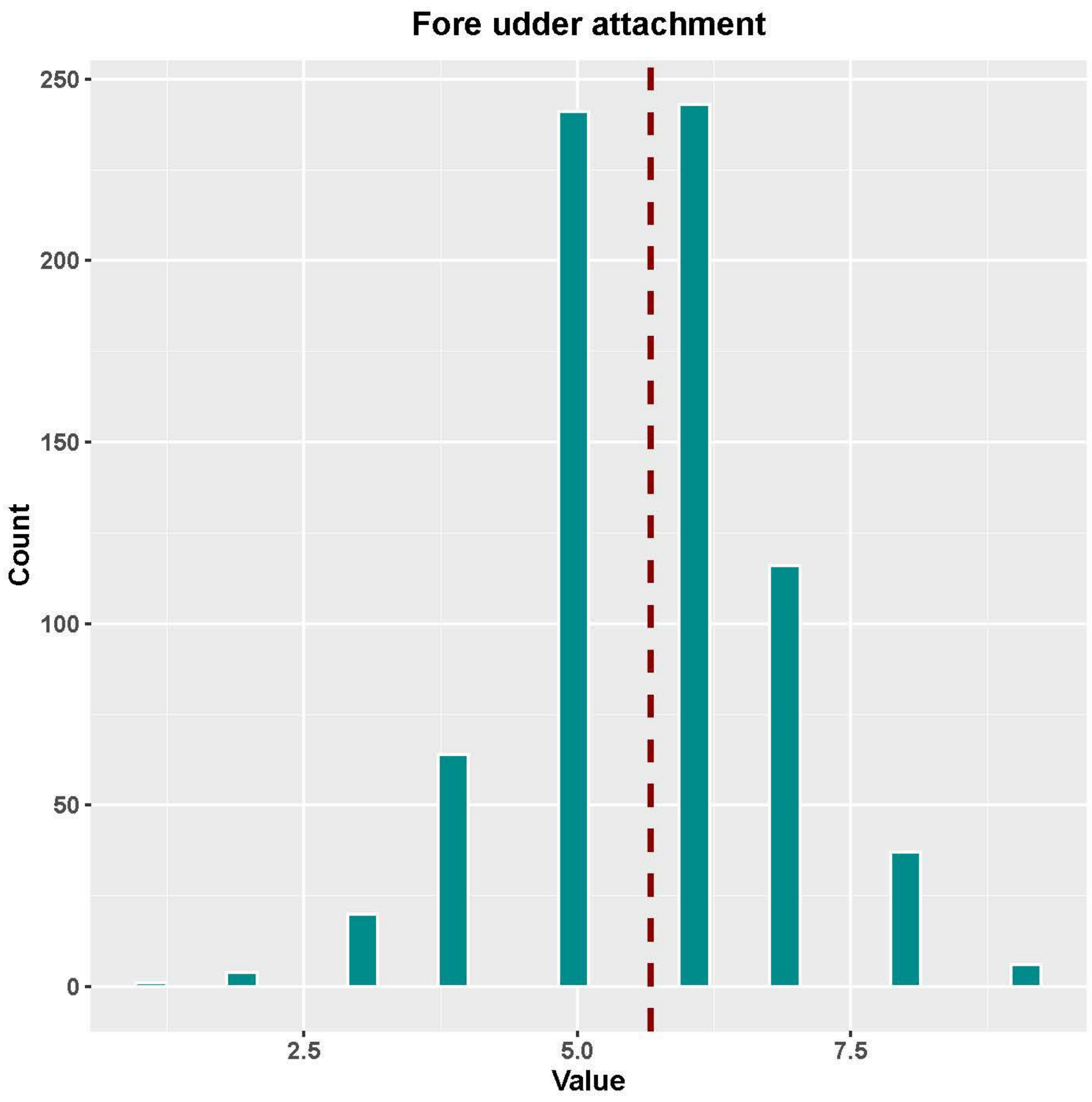
Fore udder attachment
Count
250
200
150
100
50
0
2.5
5.0
7.5
Value

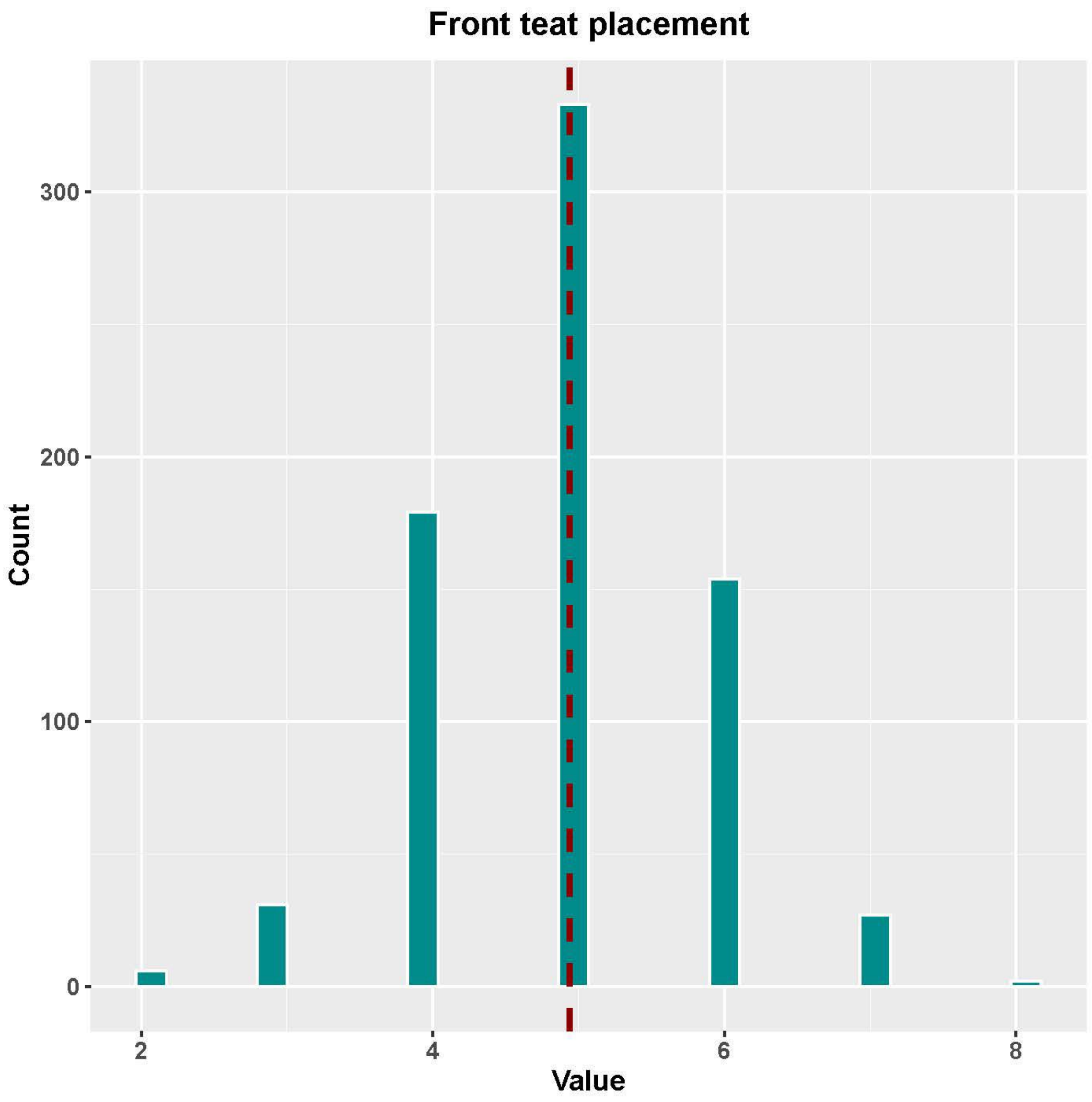

Front teat placement
Count
Value
0
100
200
300
2
4
6
8

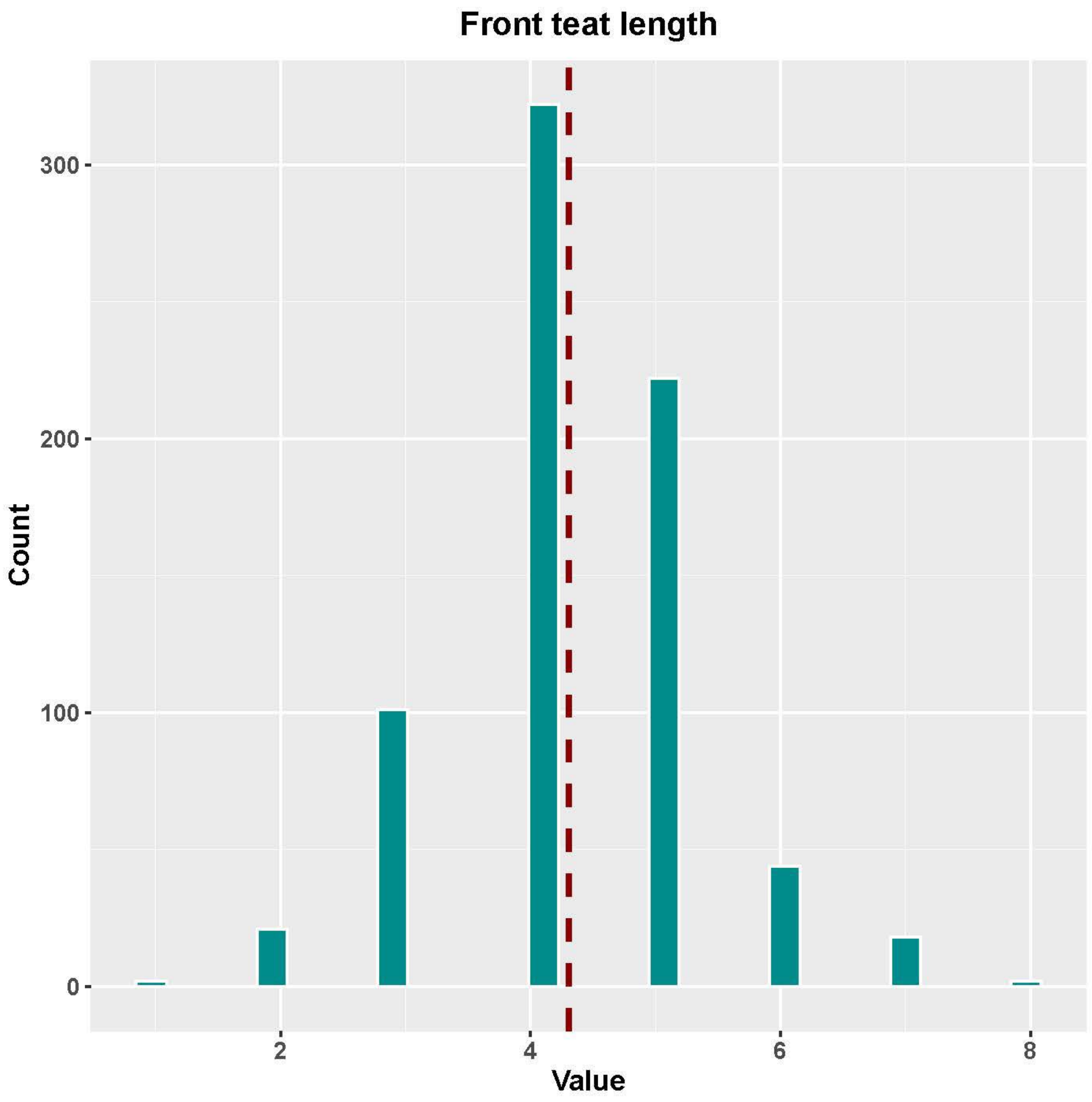

Front teat length
Count
300
200
100
0
2
4
6
8
Value

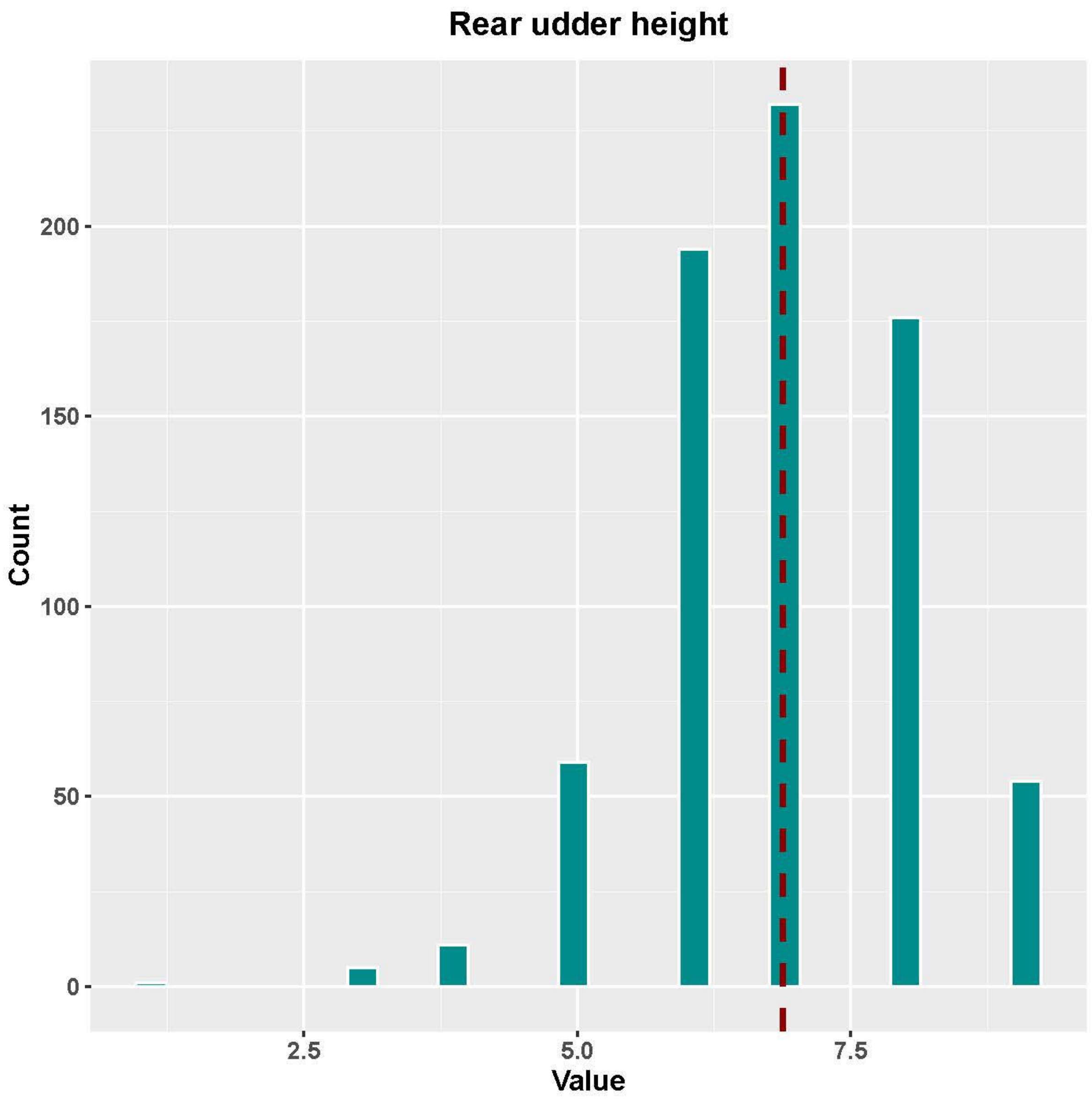
Rear udder height
Count
200
150
100
50
0
2.5
5.0
7.5
Value

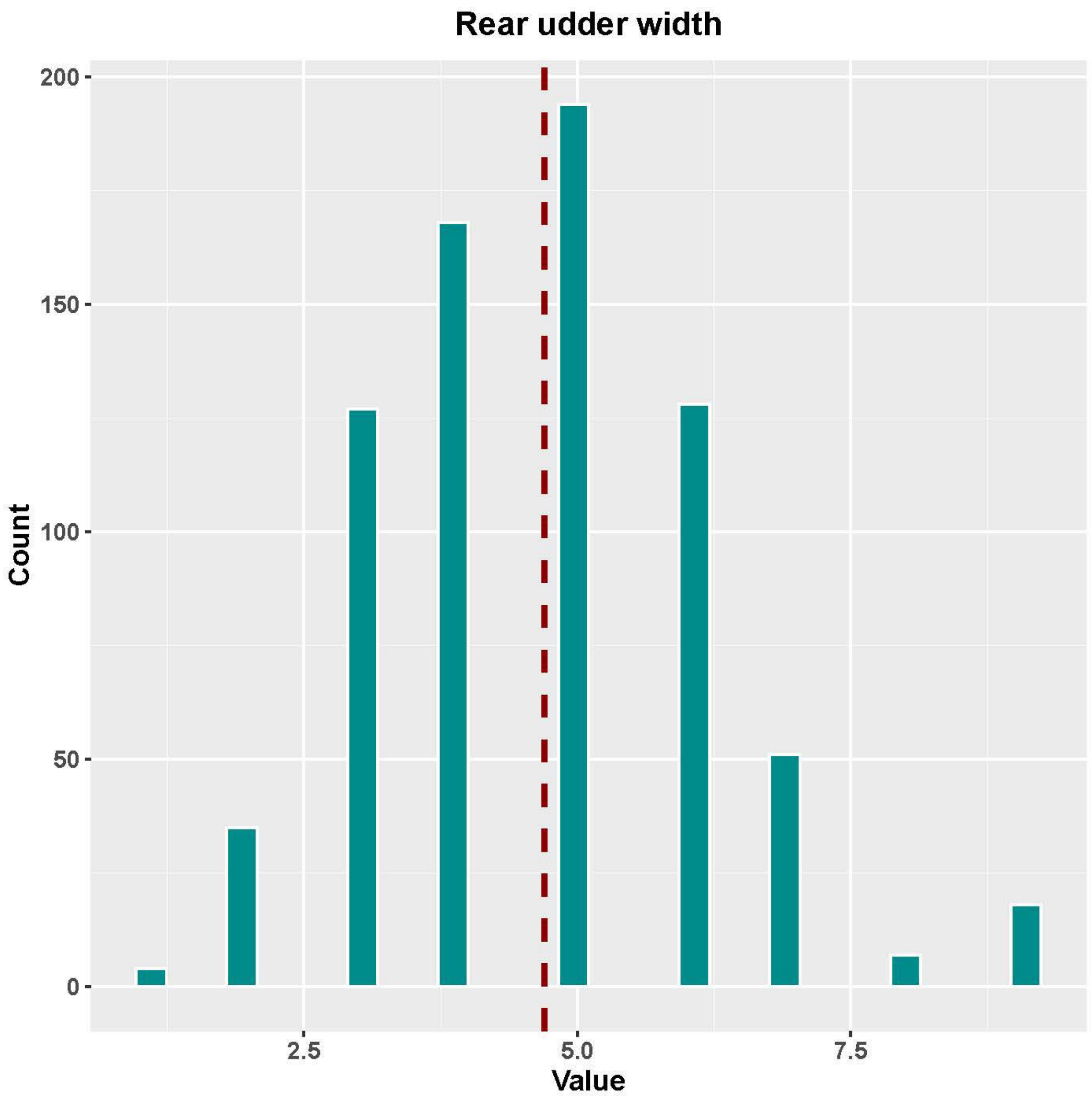

Rear udder width
Count
Value
200
150
100
50
0
2.5
5.0
7.5

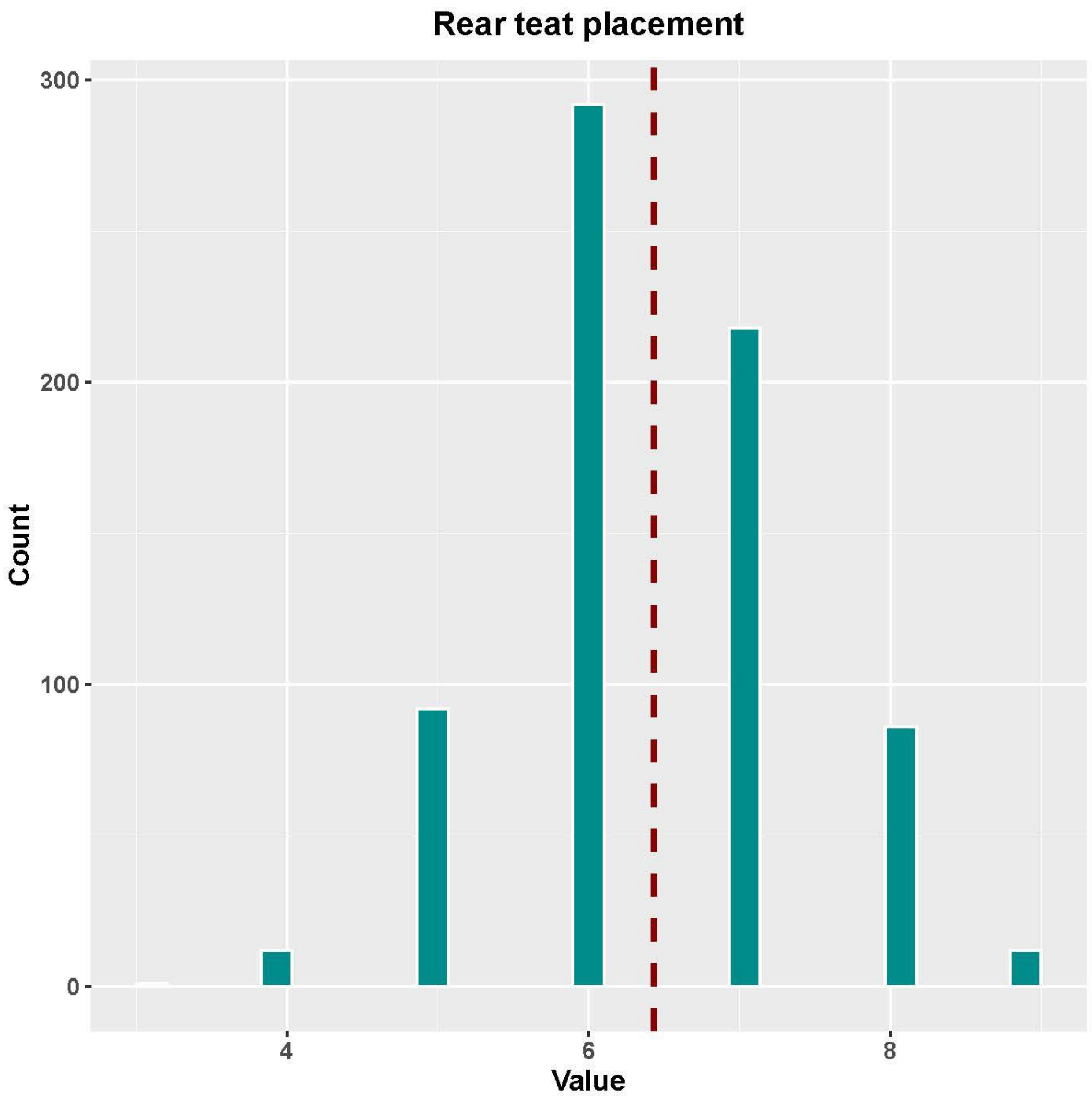

Figure S1. Phenotypic distribution of 24 body conformation traits of Korean Holstein.

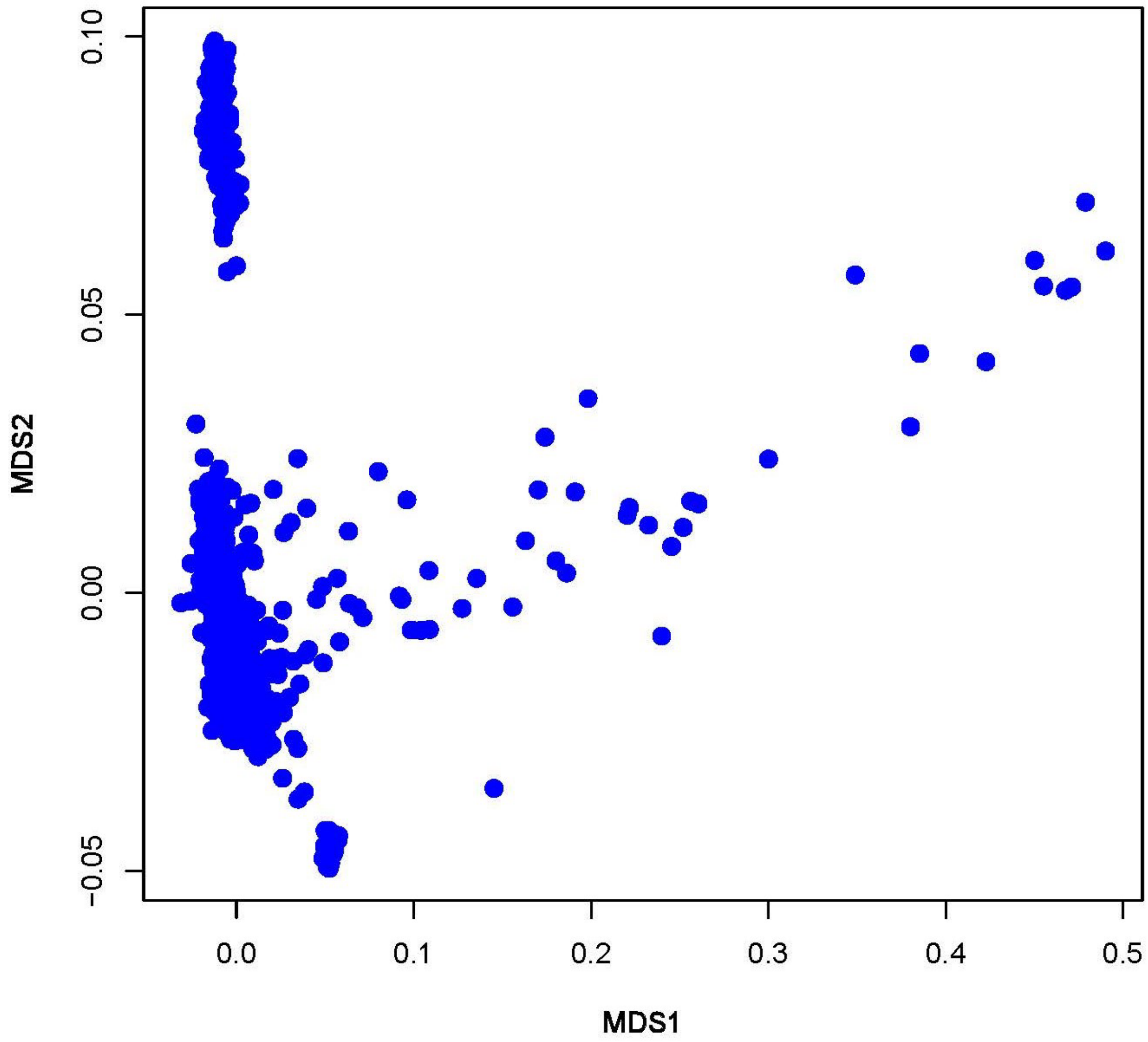

Figure S2. Two dimensional (C1 and C2) MDS plot based on data from Korean Holstein population.

(a). Stature

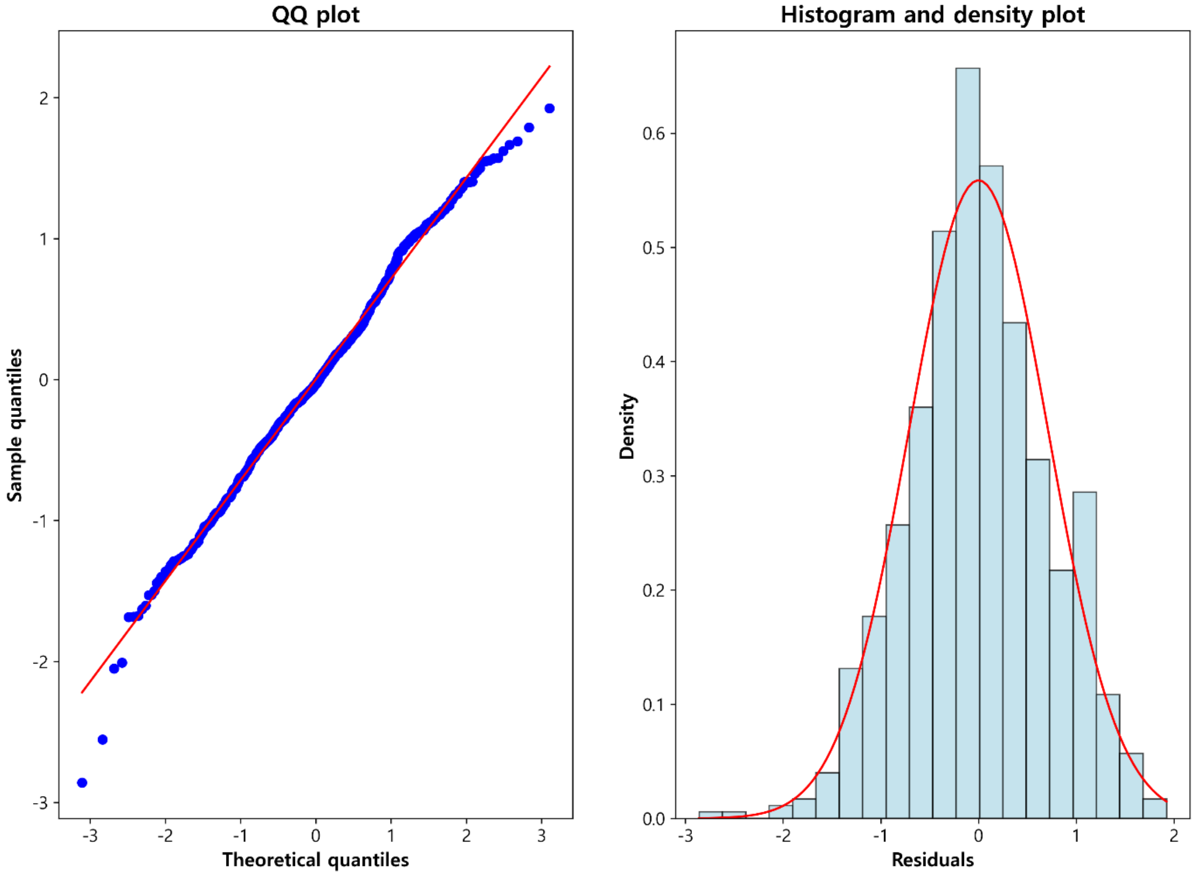
QQ plot
Sample quantiles
Theoretical quantiles
2
1
0
-1
-2
-3
-3
-2
-1
0
1
2
3
Histogram and density plot
Density
Residuals
0.6
0.5
0.4
0.3
0.2
0.1
0.0
-3
-2
-1
0
1
2

(b). Height at front end

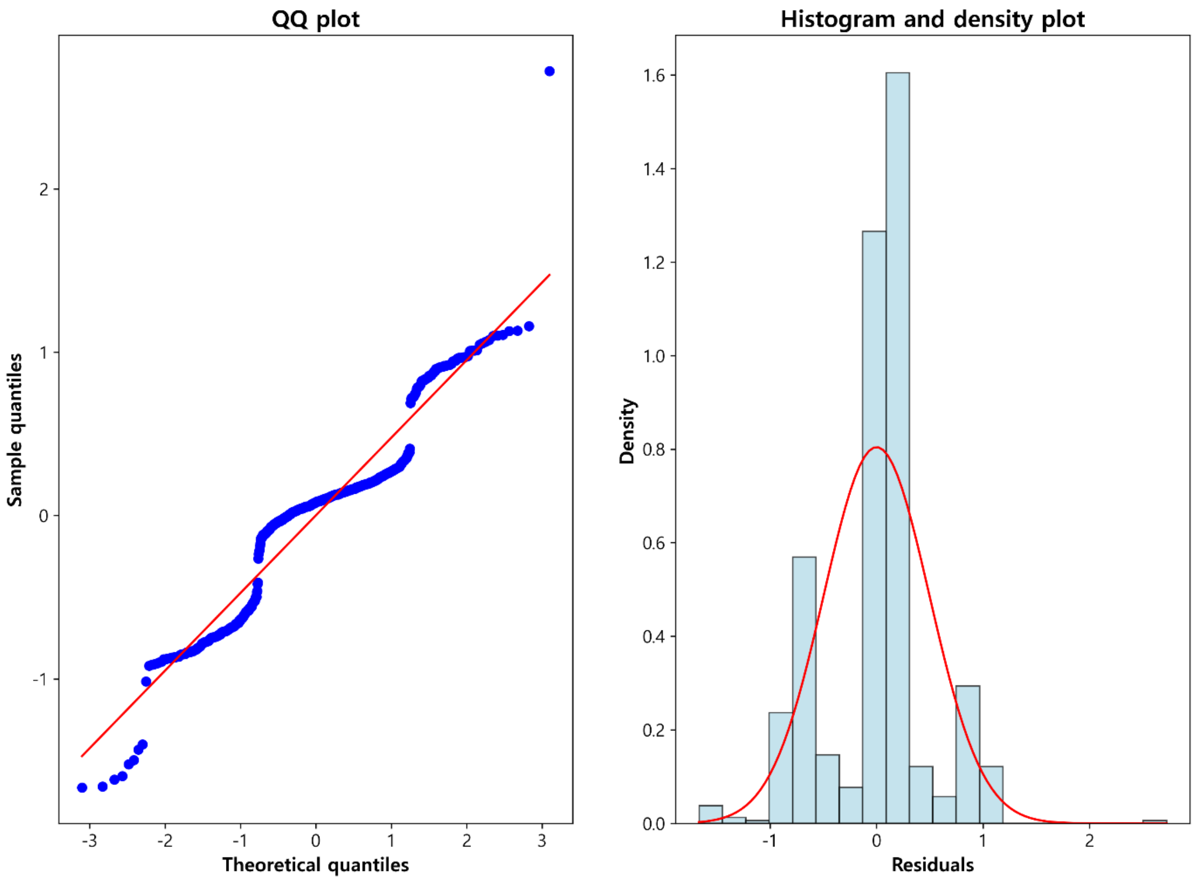

(c). Chest width

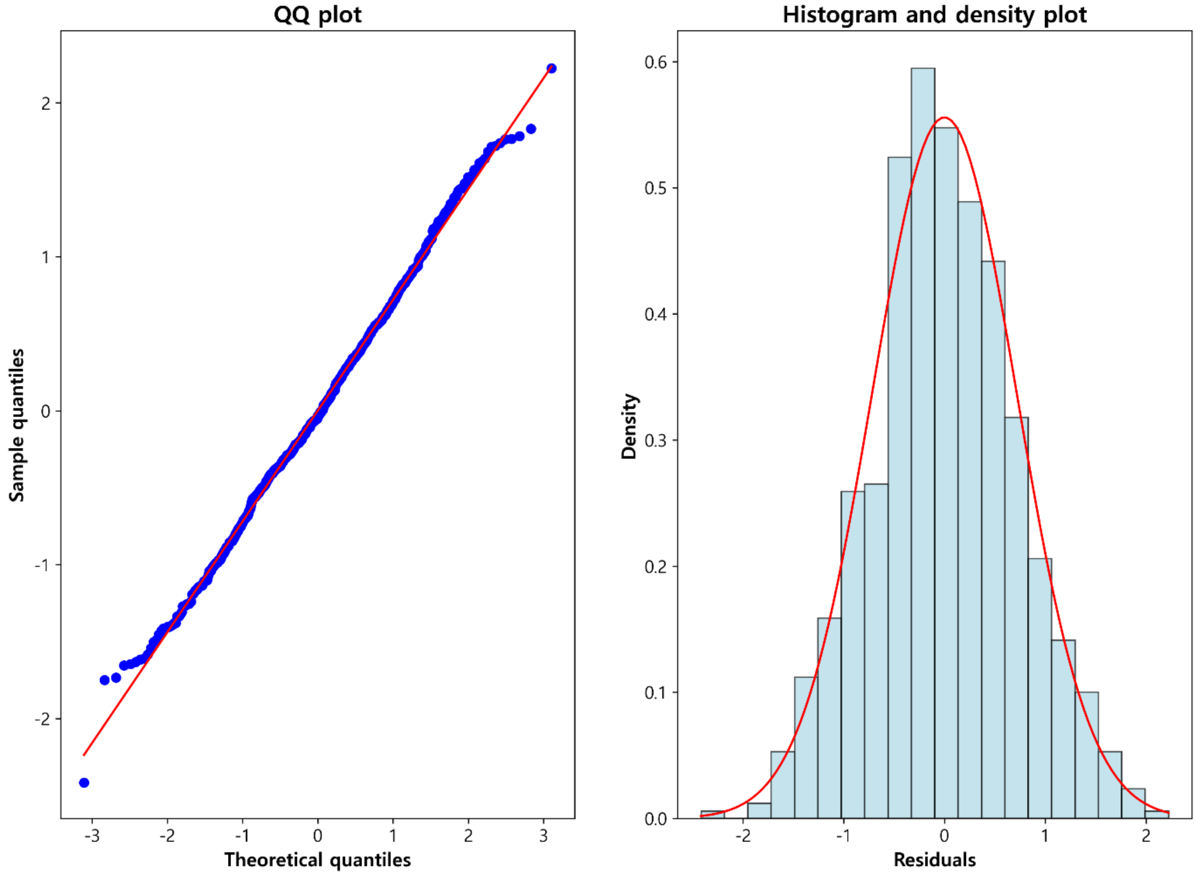

(d). Body depth

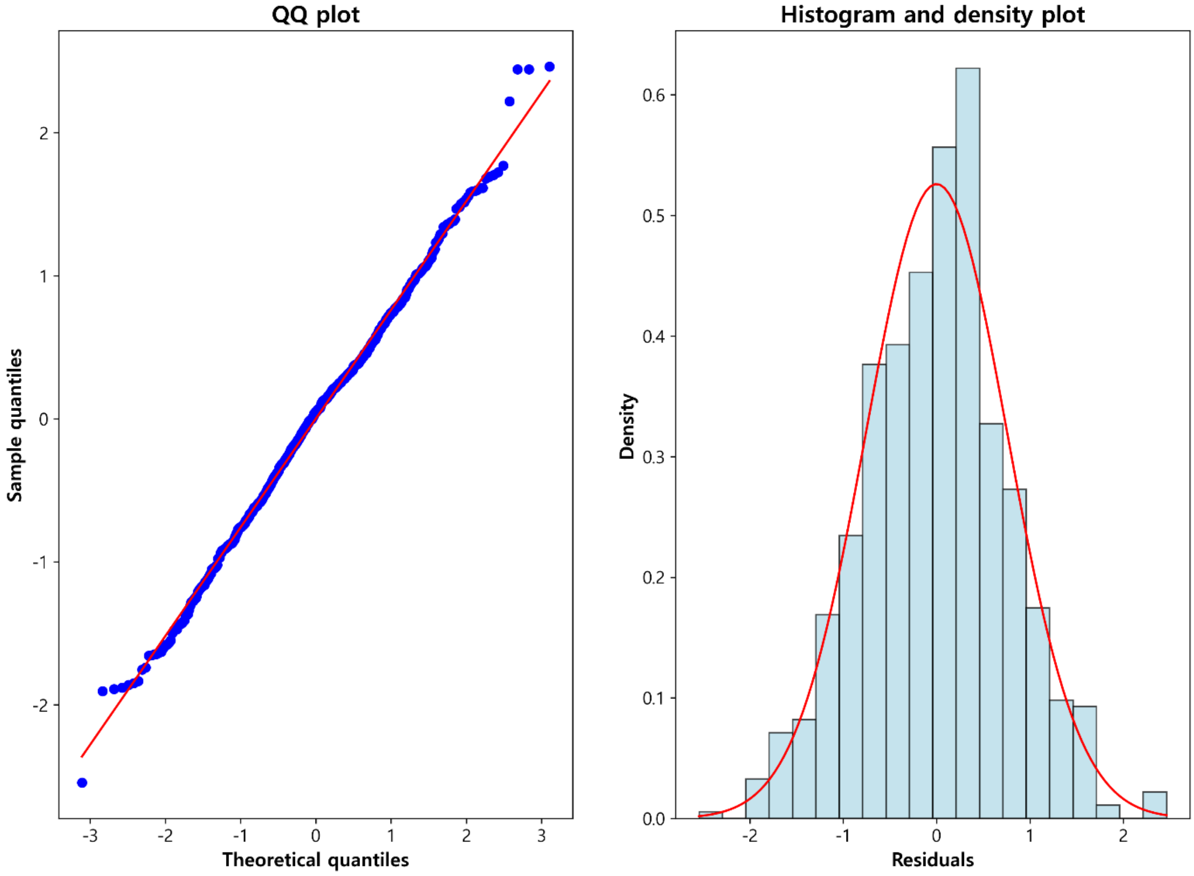

QQ plot
Sample quantiles
Theoretical quantiles
-3
-2
-1
0
1
2
3
Histogram and density plot
Density
Residuals
0.0
0.1
0.2
0.3
0.4
0.5
0.6

(e). Angularity

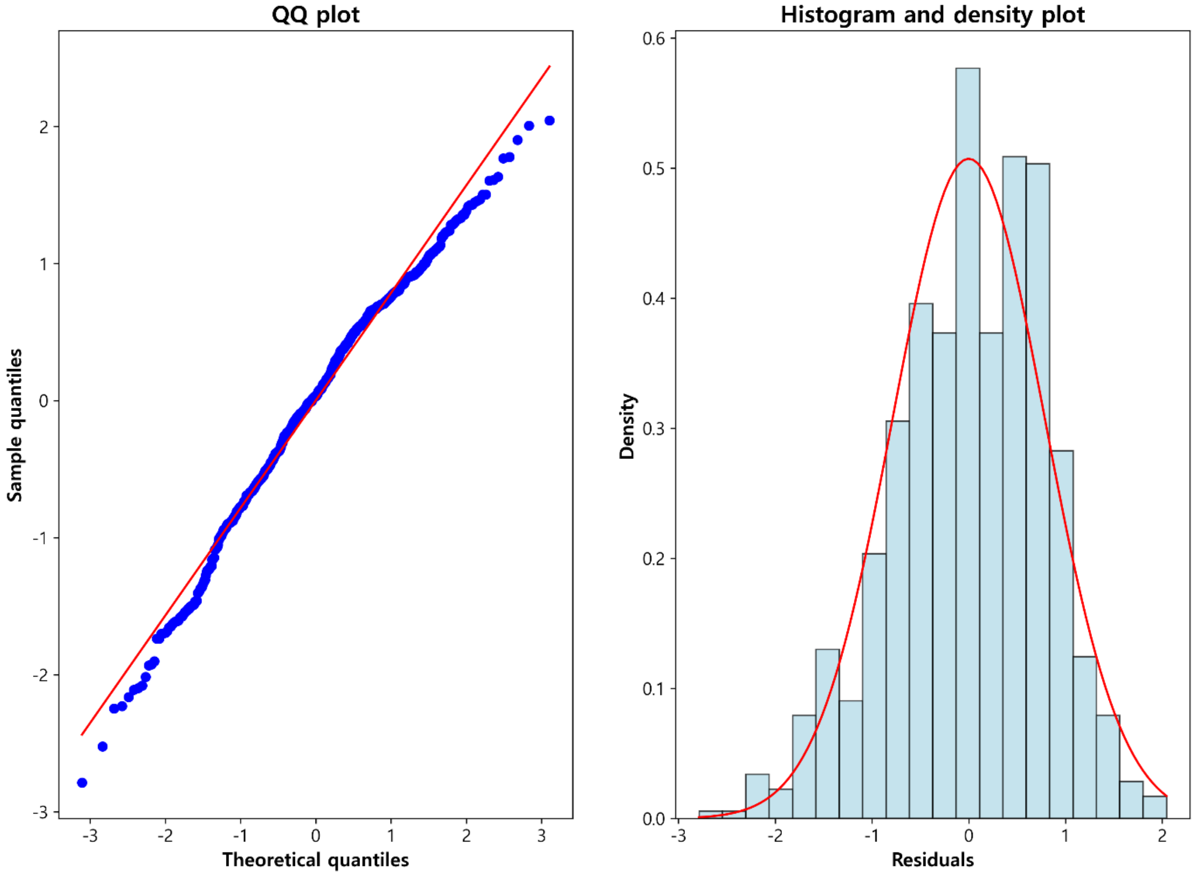

(f). Body condition score

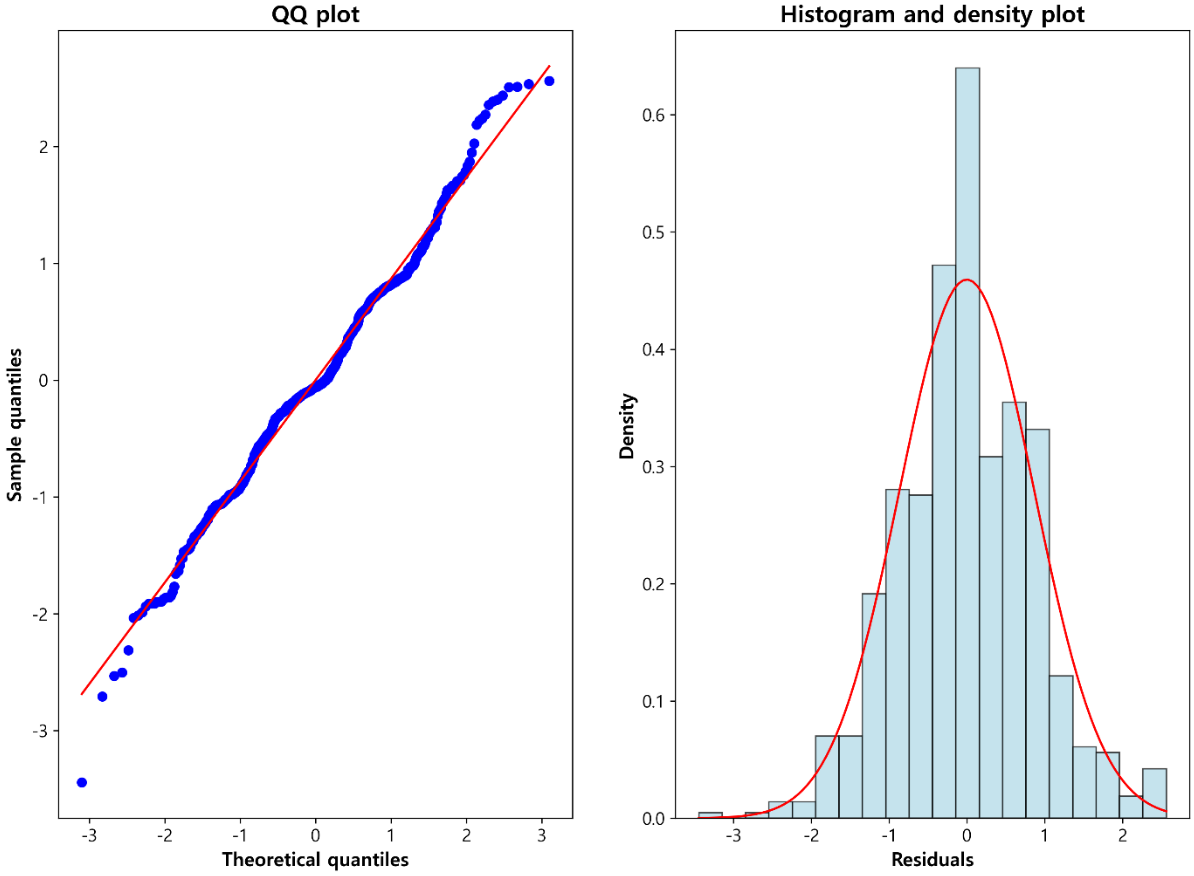

(g). Locomotion

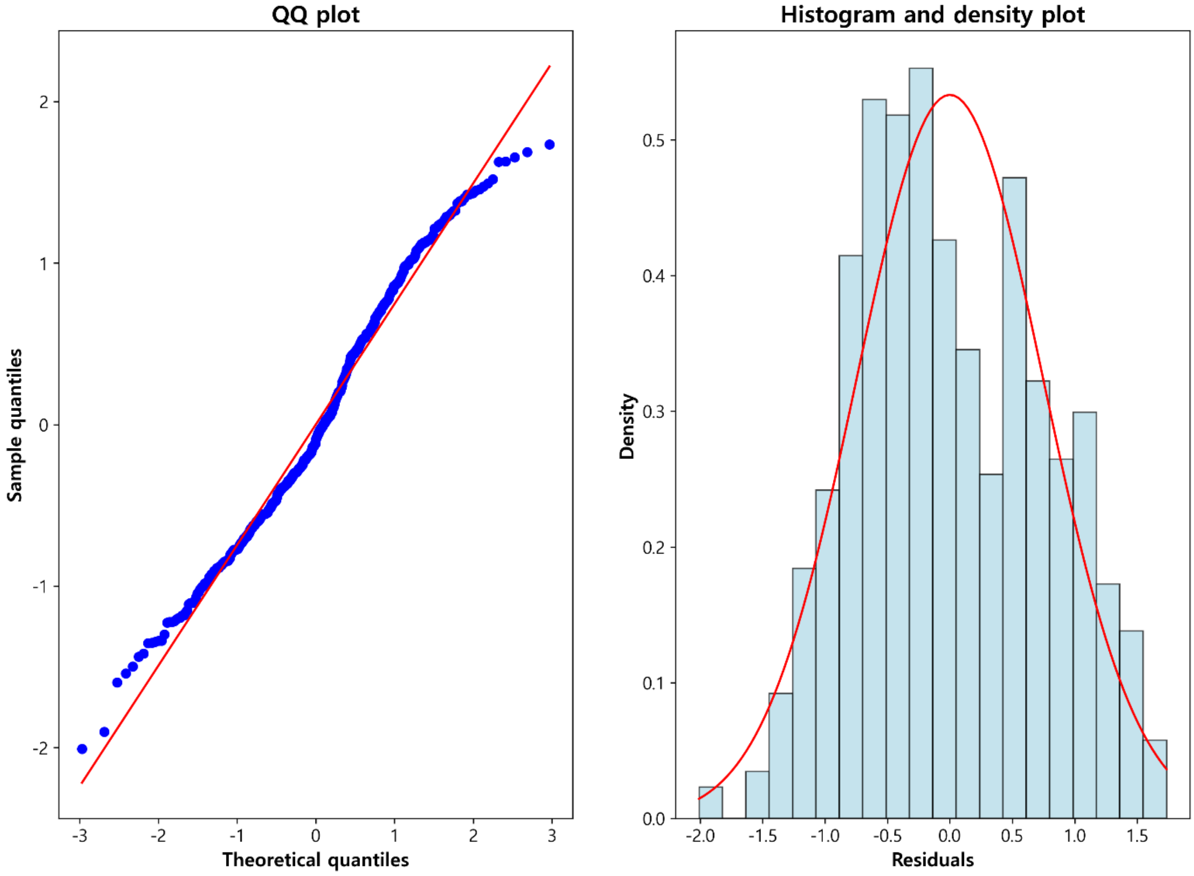

(h). Rump angle

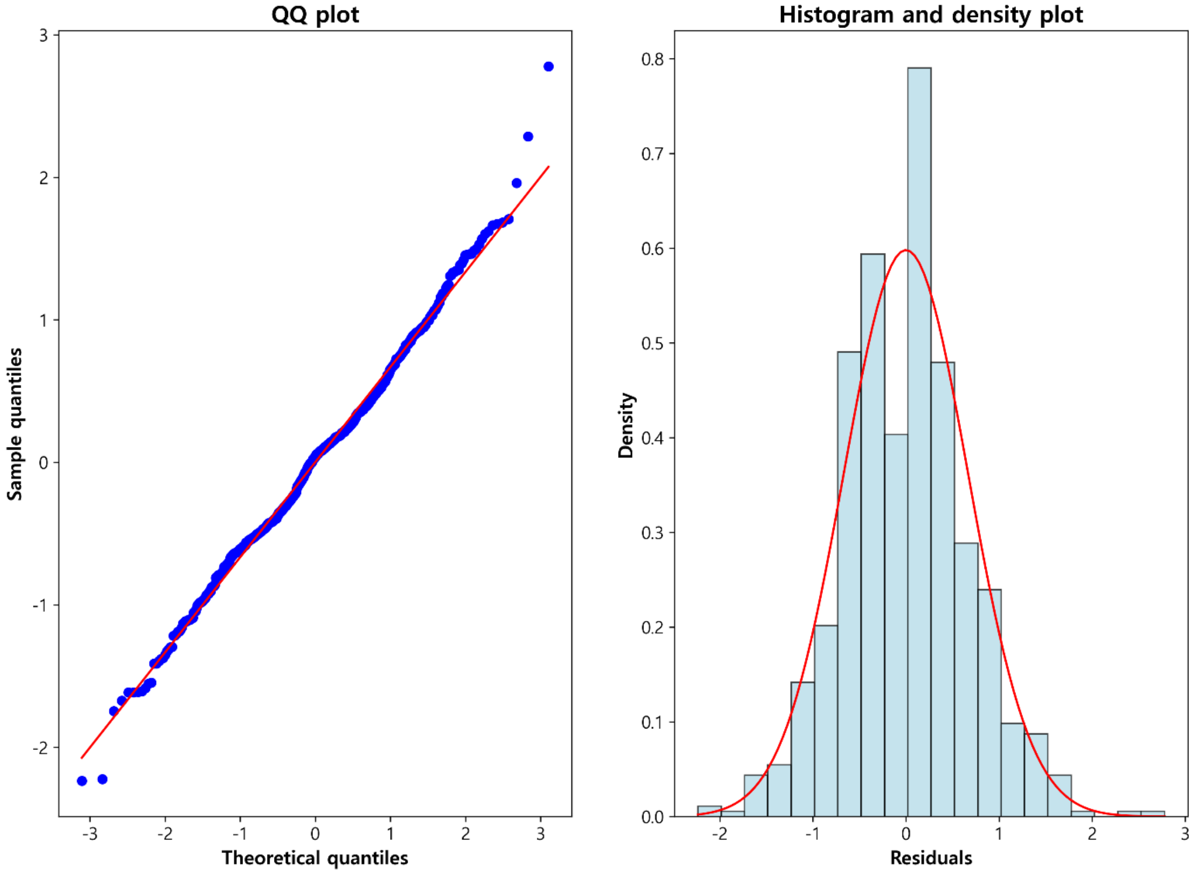

QQ plot
Sample quantiles
Theoretical quantiles
3
2
1
0
-1
-2
-3
-2
-1
0
1
2
3
Histogram and density plot
Density
Residuals
0.8
0.7
0.6
0.5
0.4
0.3
0.2
0.1
0.0
-2
-1
0
1
2
3

(i). Rump width

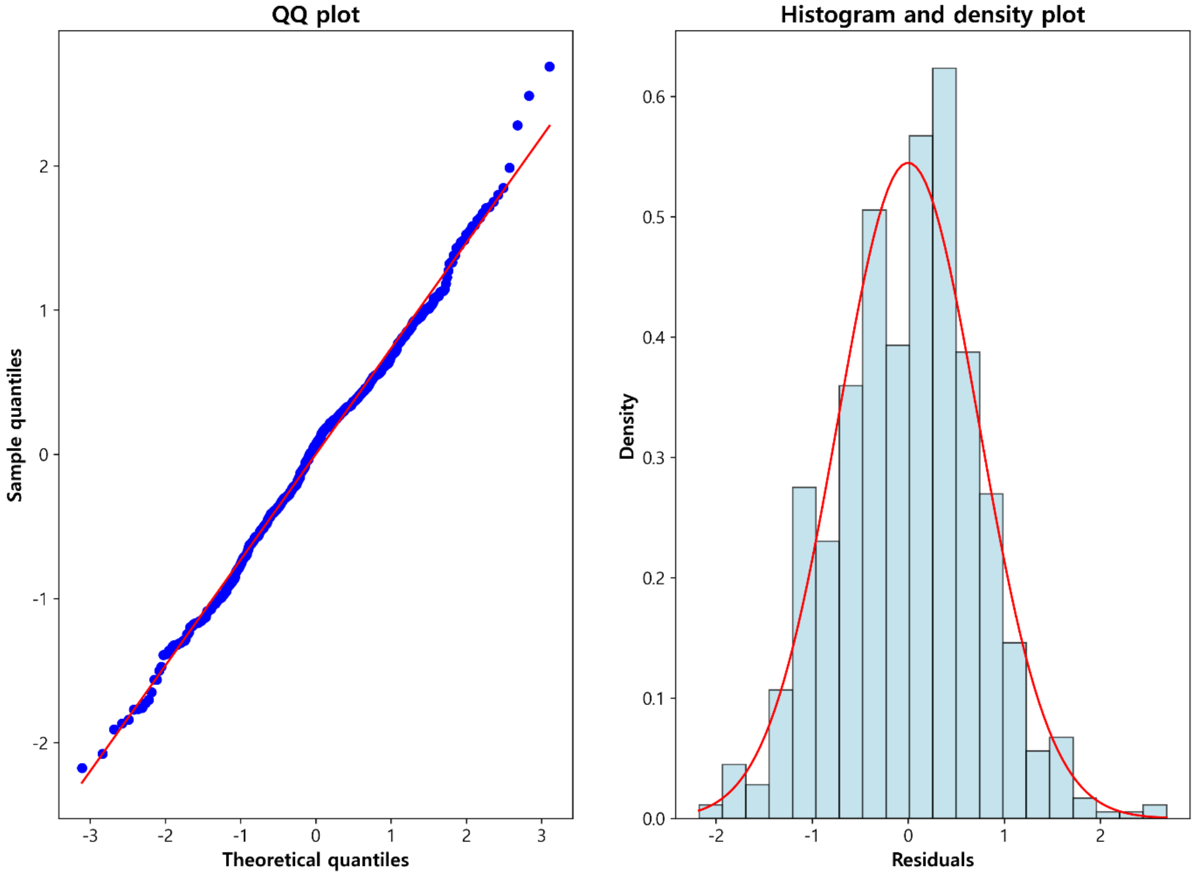

QQ plot
Sample quantiles
Theoretical quantiles
Histogram and density plot
Density
Residuals

(j). Loin strength

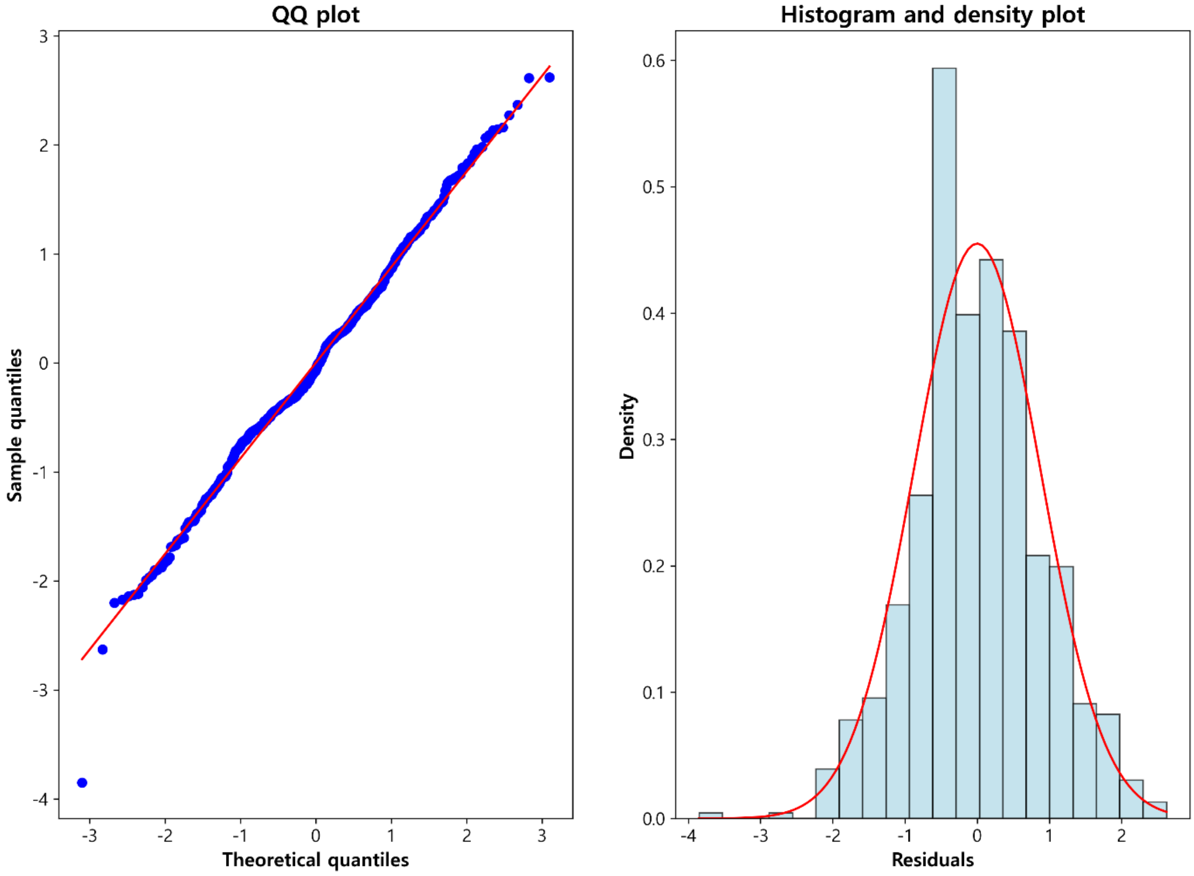

(k). Rear leg set

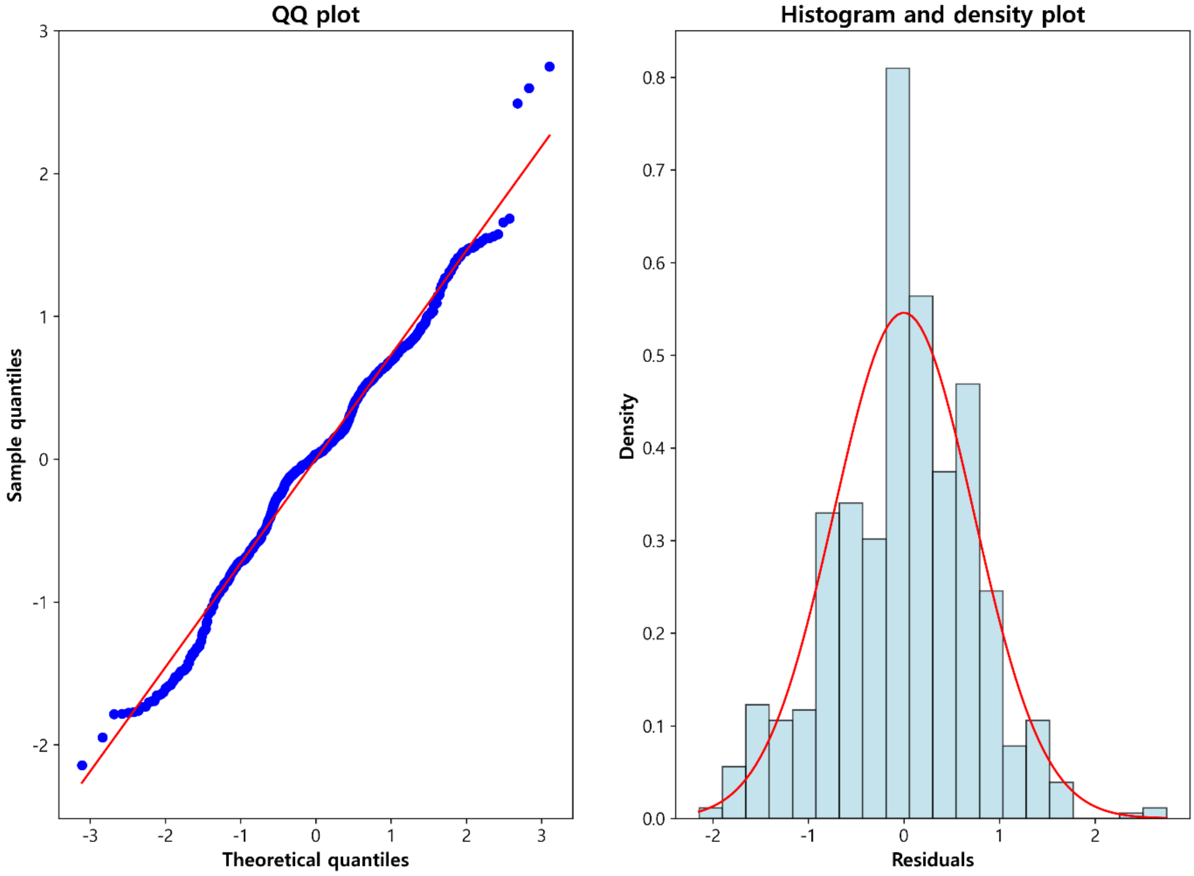

(l). Rear leg rear view

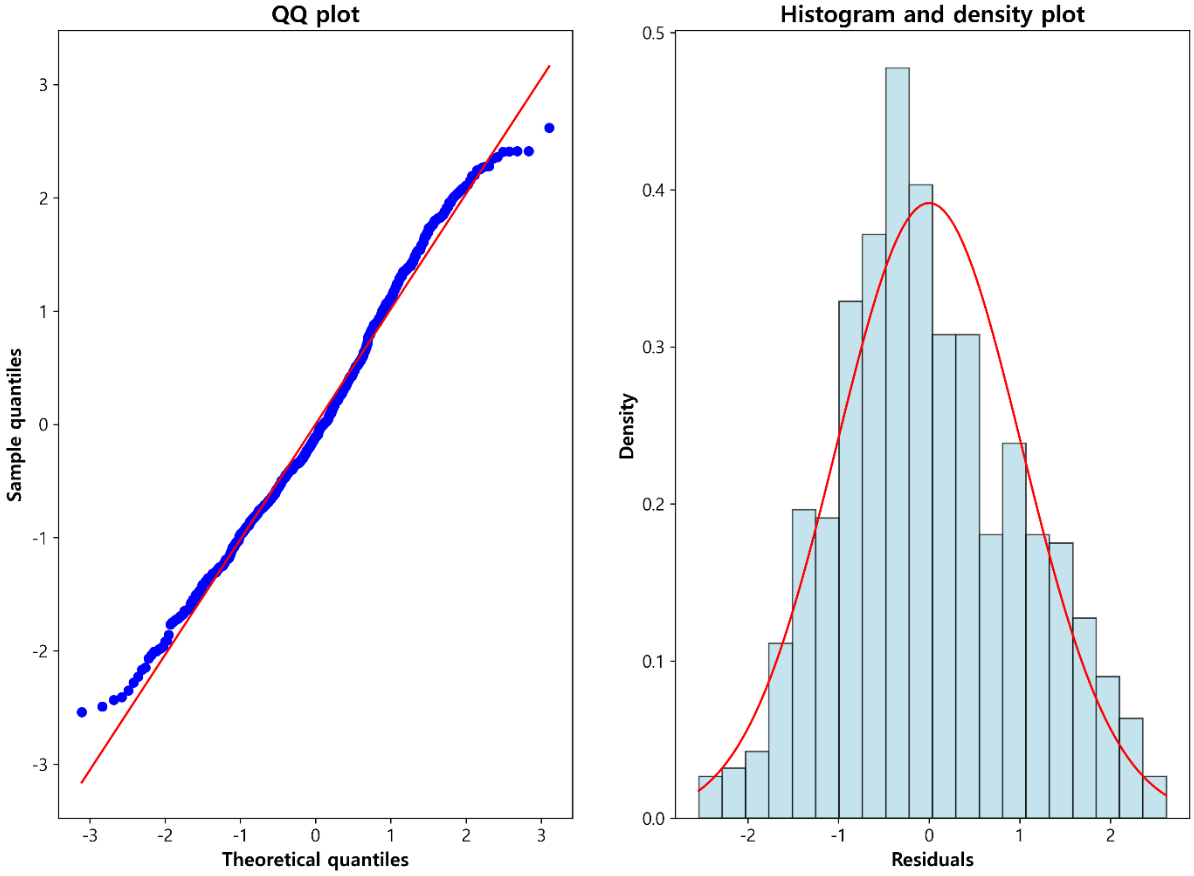

(m). Foot angle

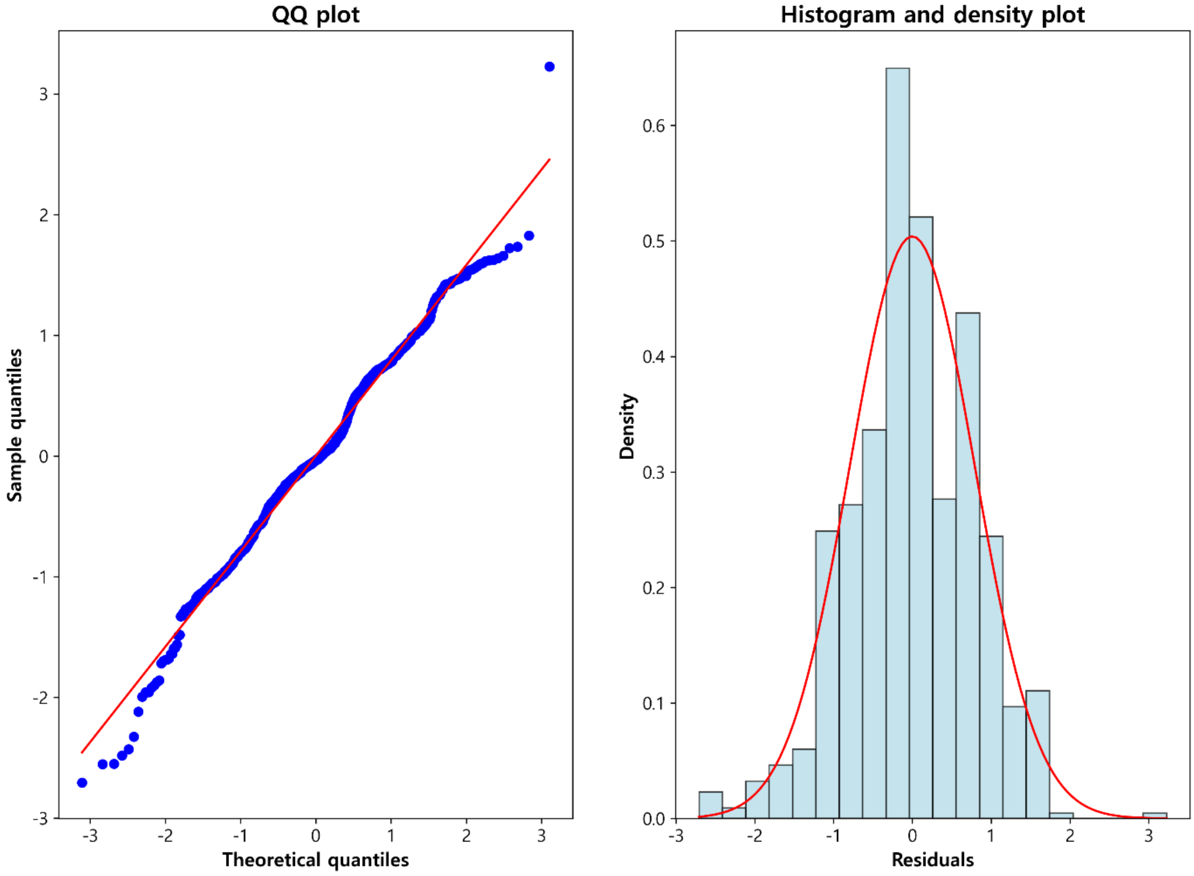
QQ plot
Sample quantiles
Theoretical quantiles
Histogram and density plot
Density
Residuals

(n). Heel depth

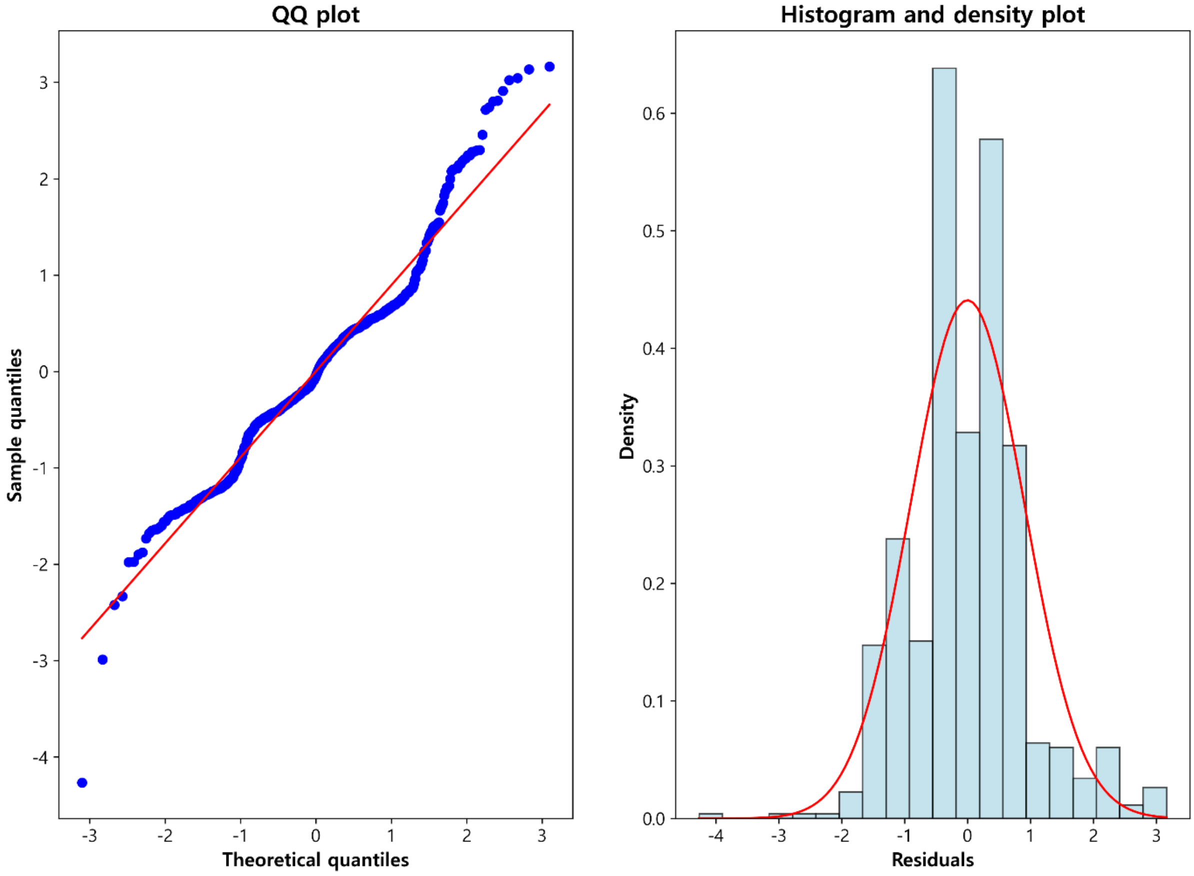
QQ plot
Sample quantiles
Theoretical quantiles
3
2
1
0
-1
-2
-3
-4
-3
-2
-1
0
1
2
3
Histogram and density plot
Density
Residuals
0.6
0.5
0.4
0.3
0.2
0.1
0.0
-4
-3
-2
-1
0
1
2
3

(o). Bone quality

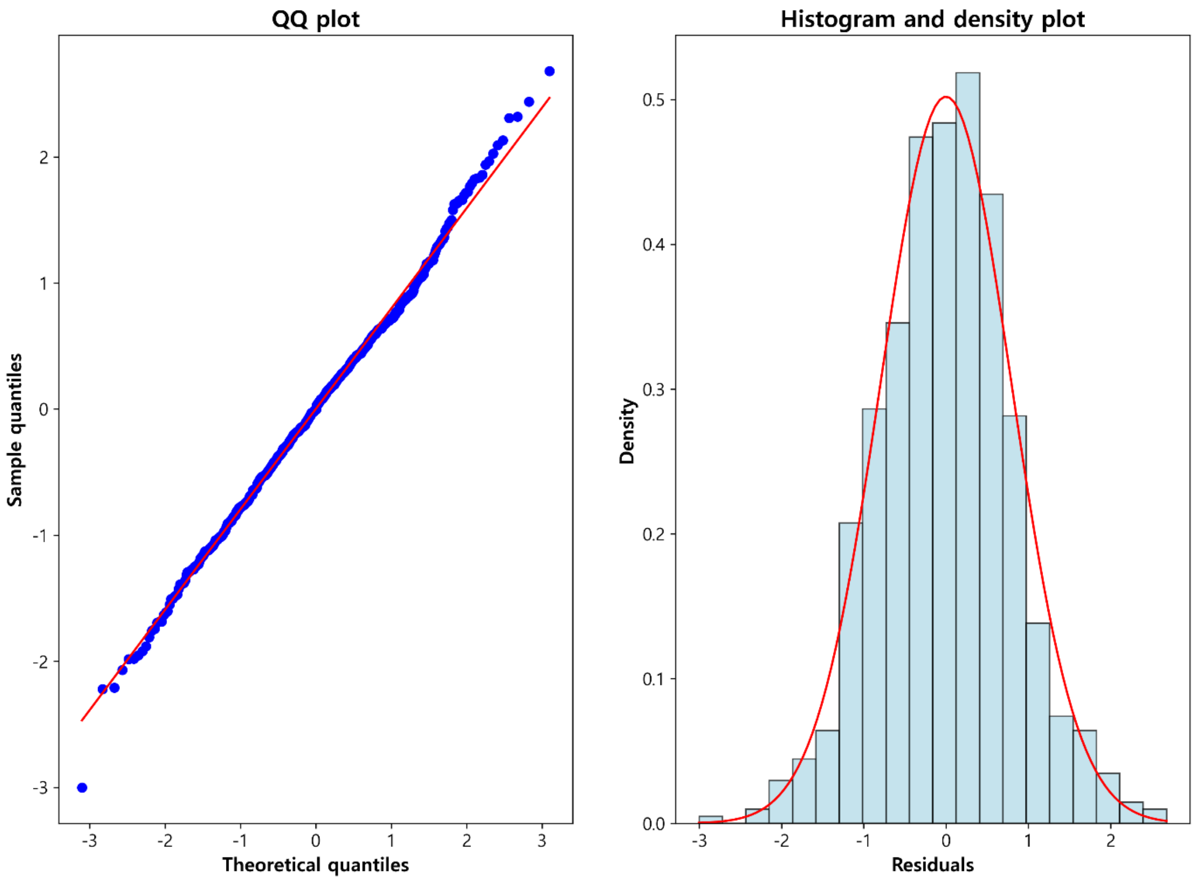

(p). Udder depth

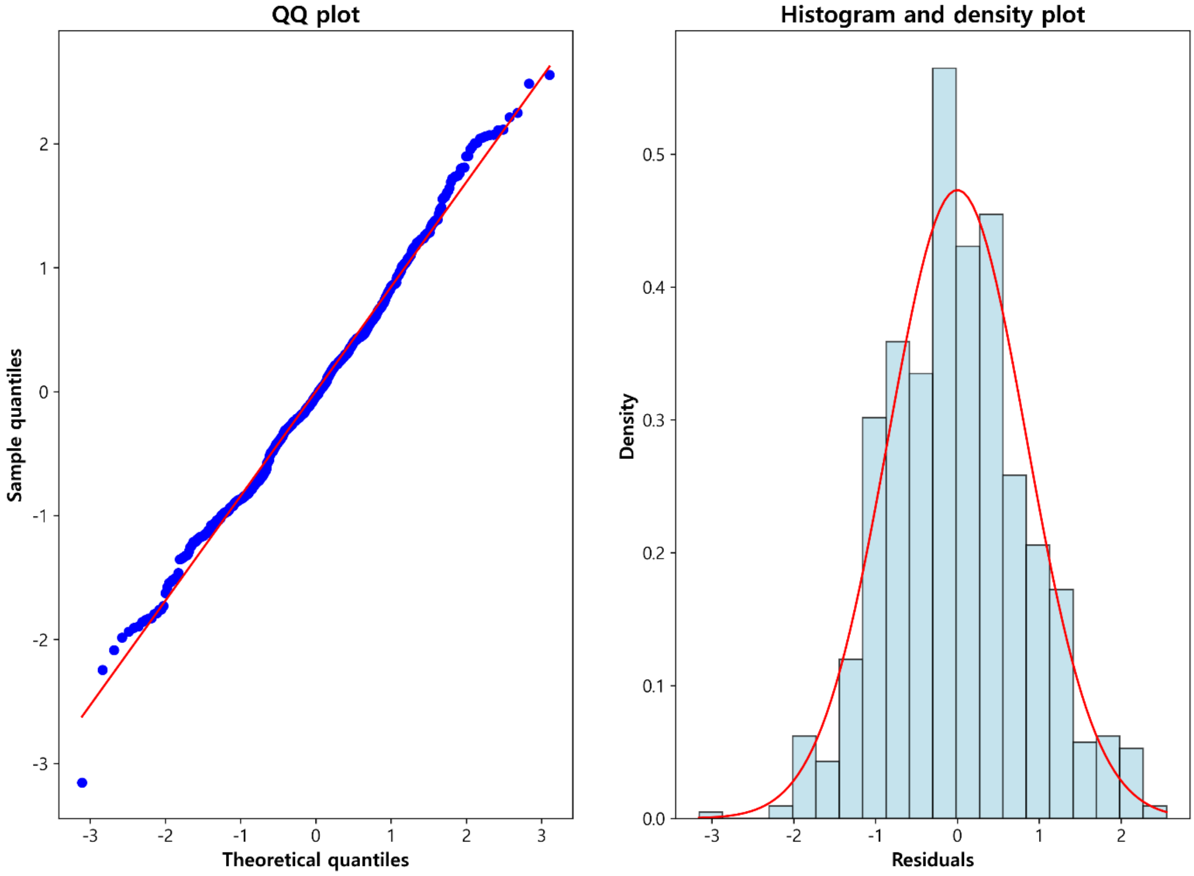

(q). Udder texture

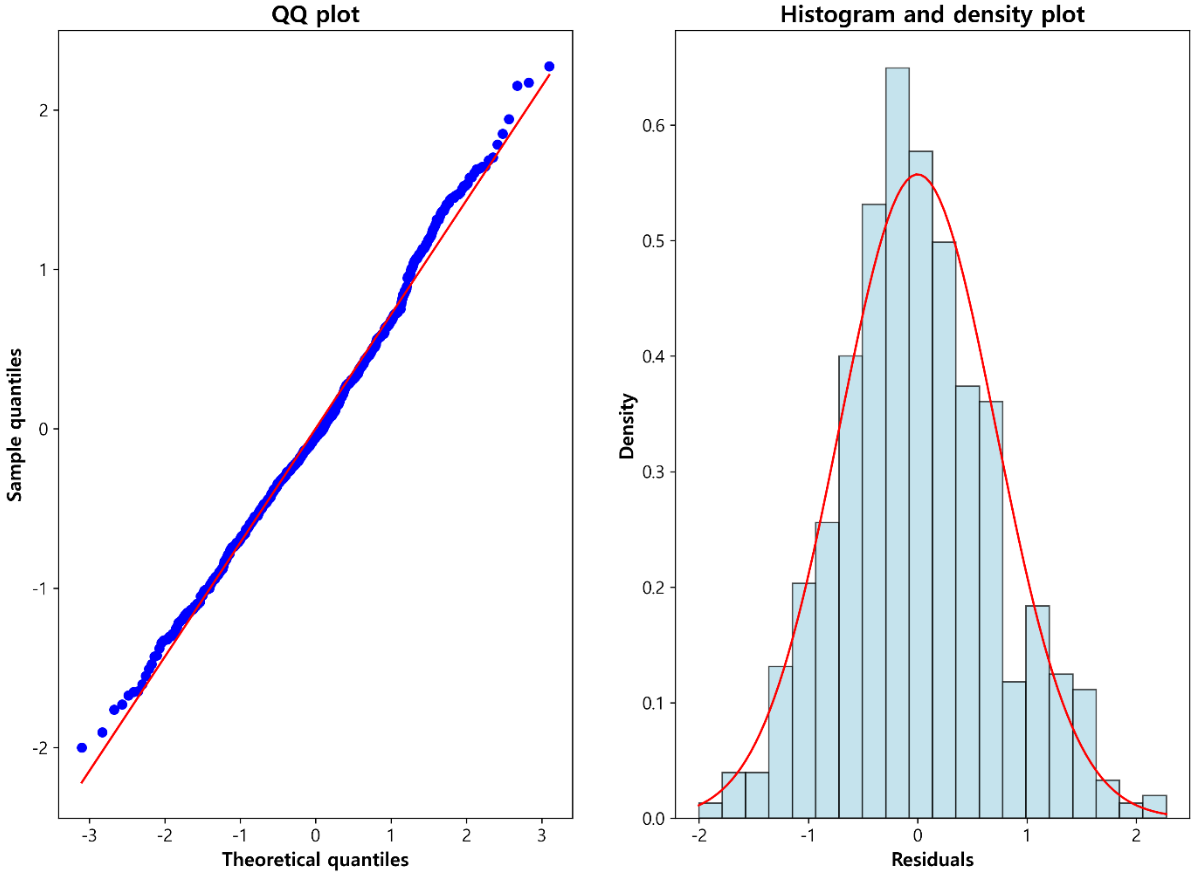

(r). Udder support

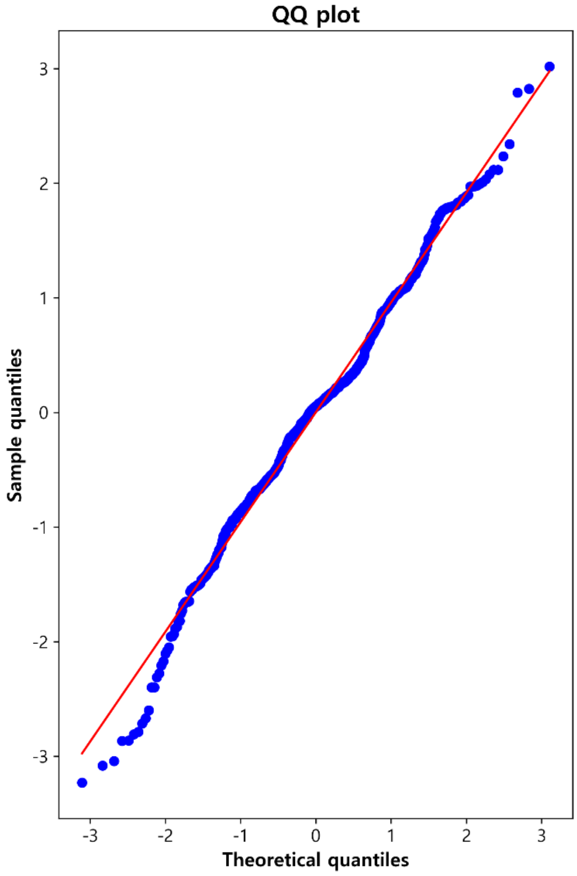

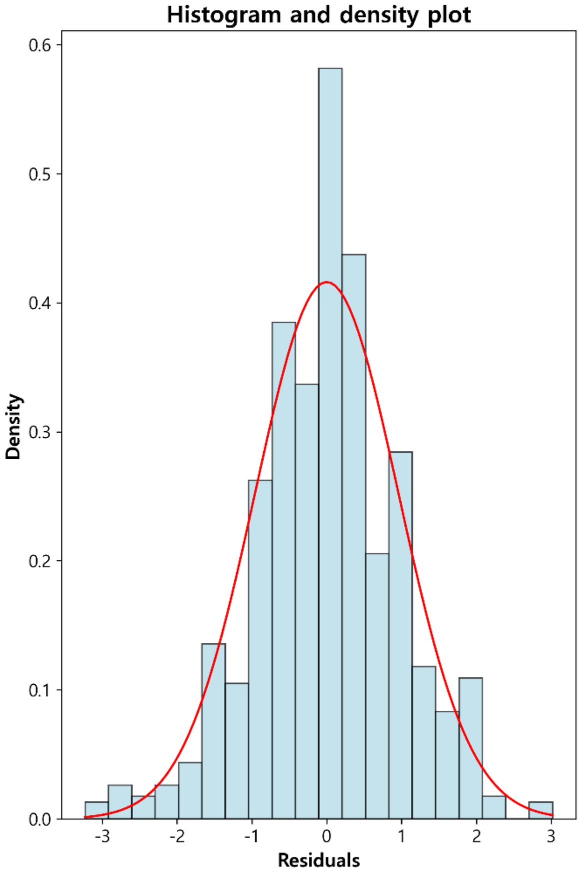

(s). Fore udder attachment

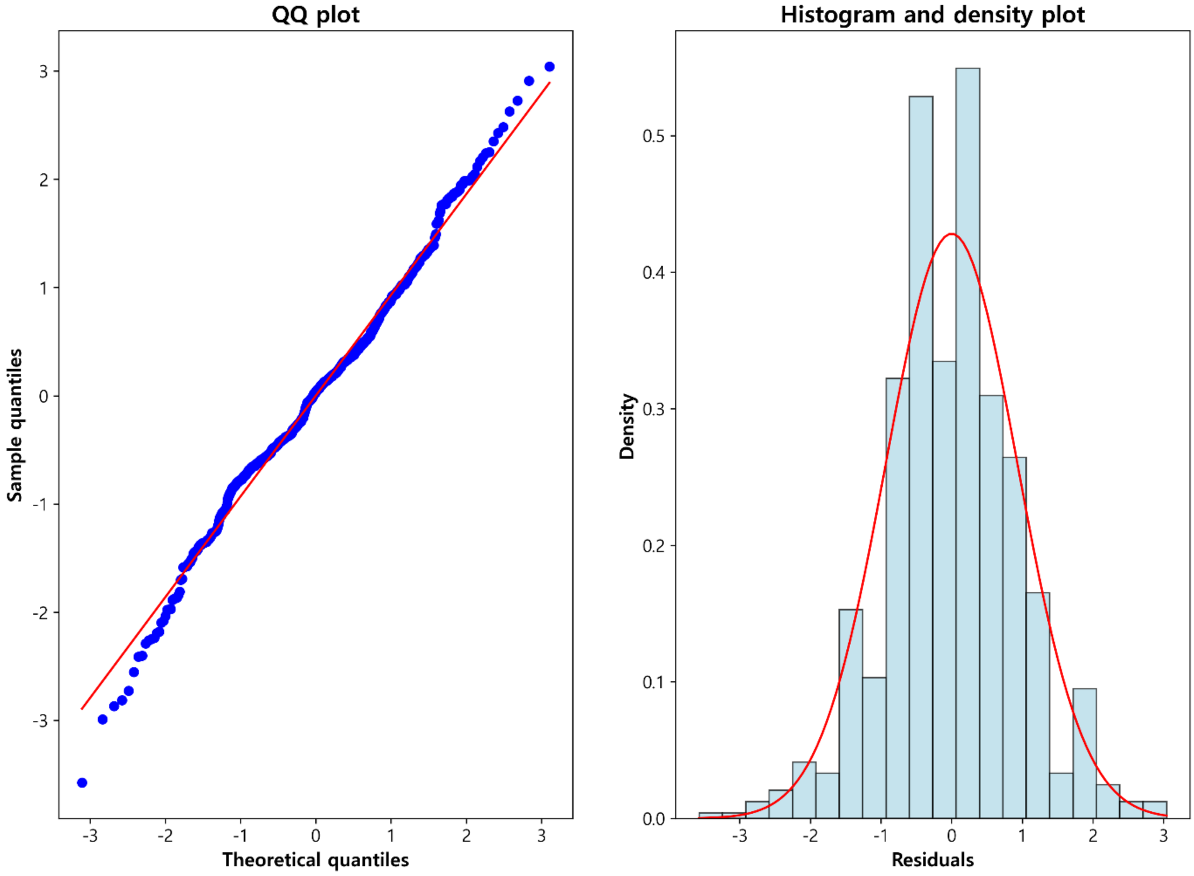

(t). Front teat placement

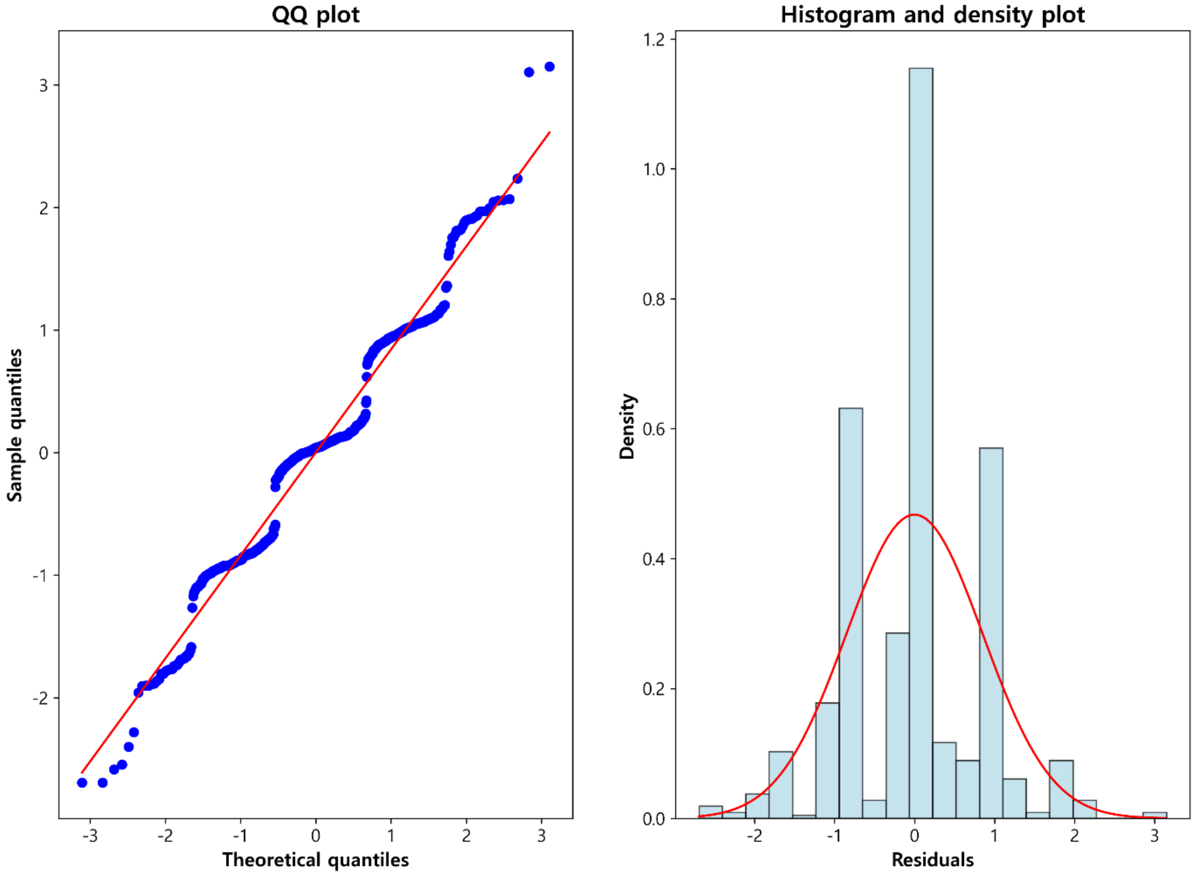

(u). Front teat length

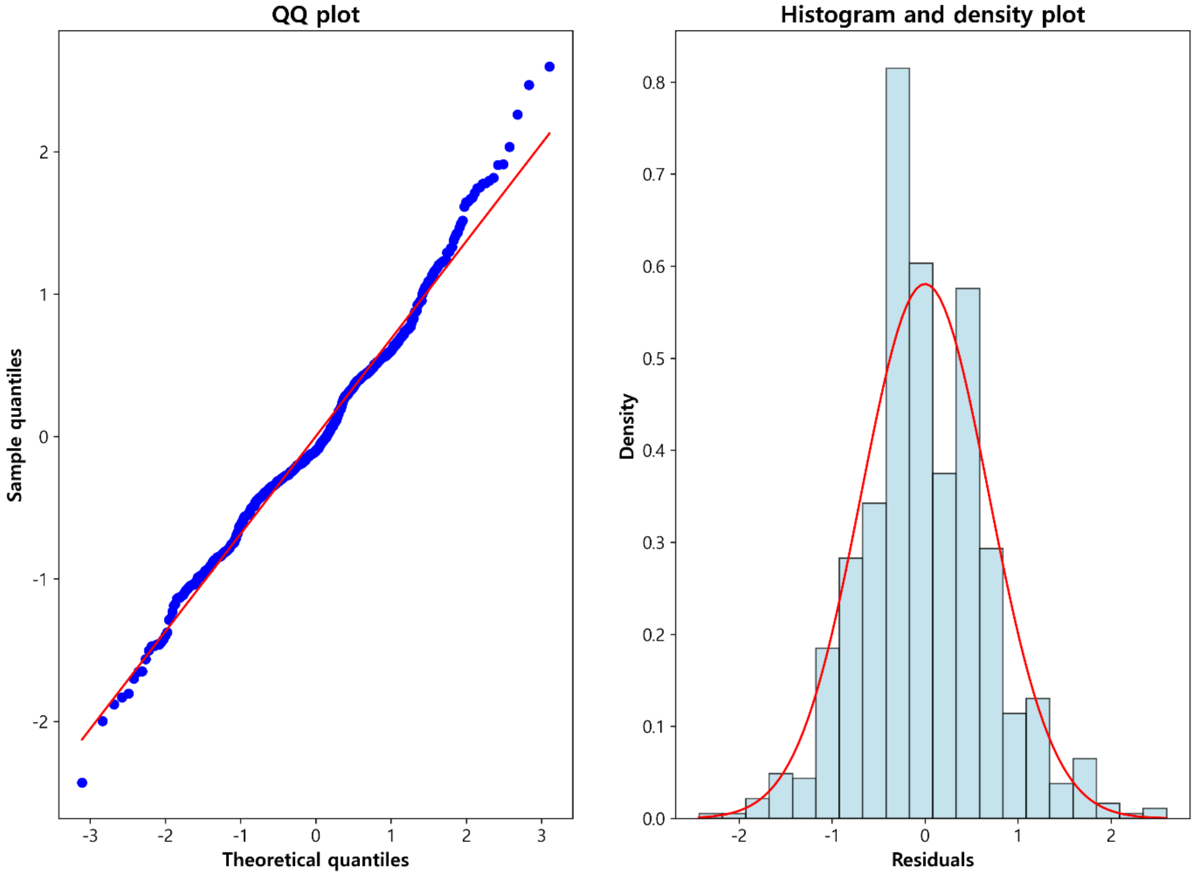

(v). Rear udder height

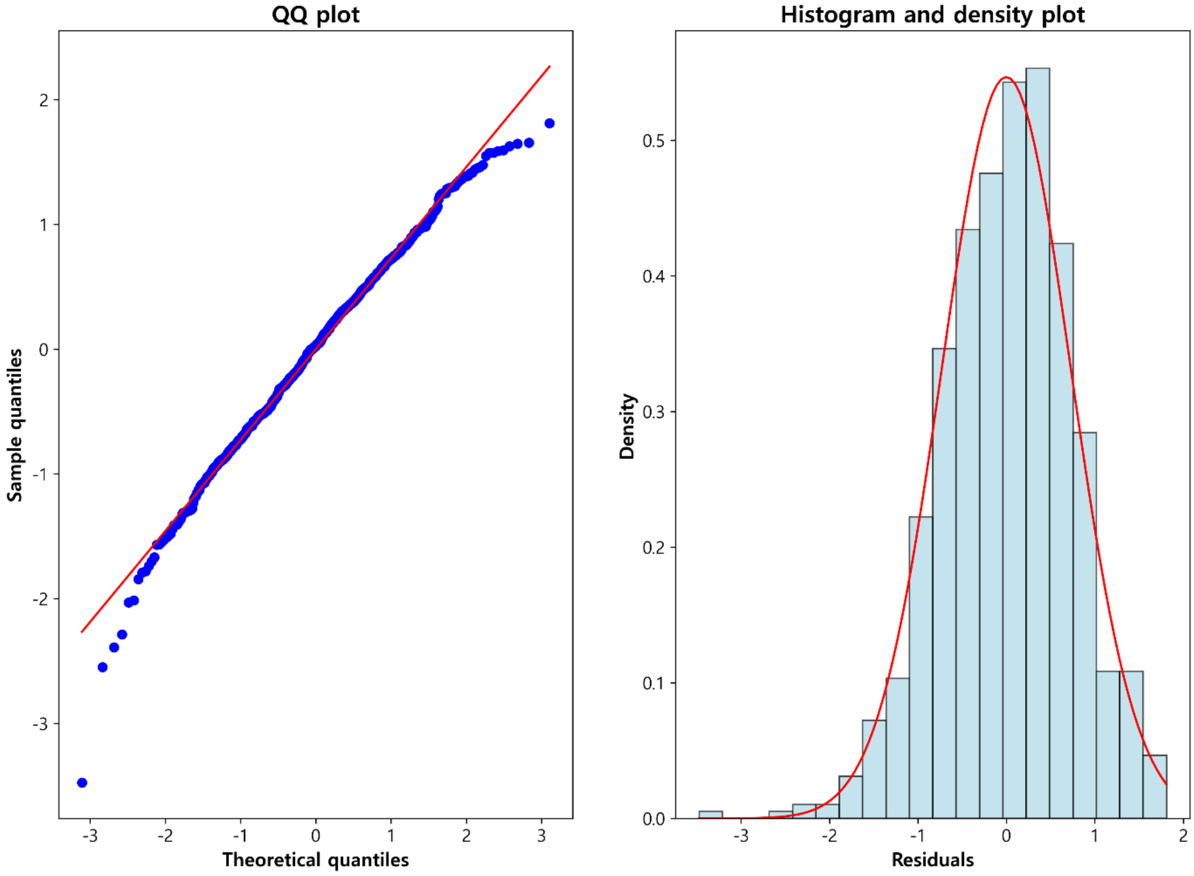

(w). Rear udder width

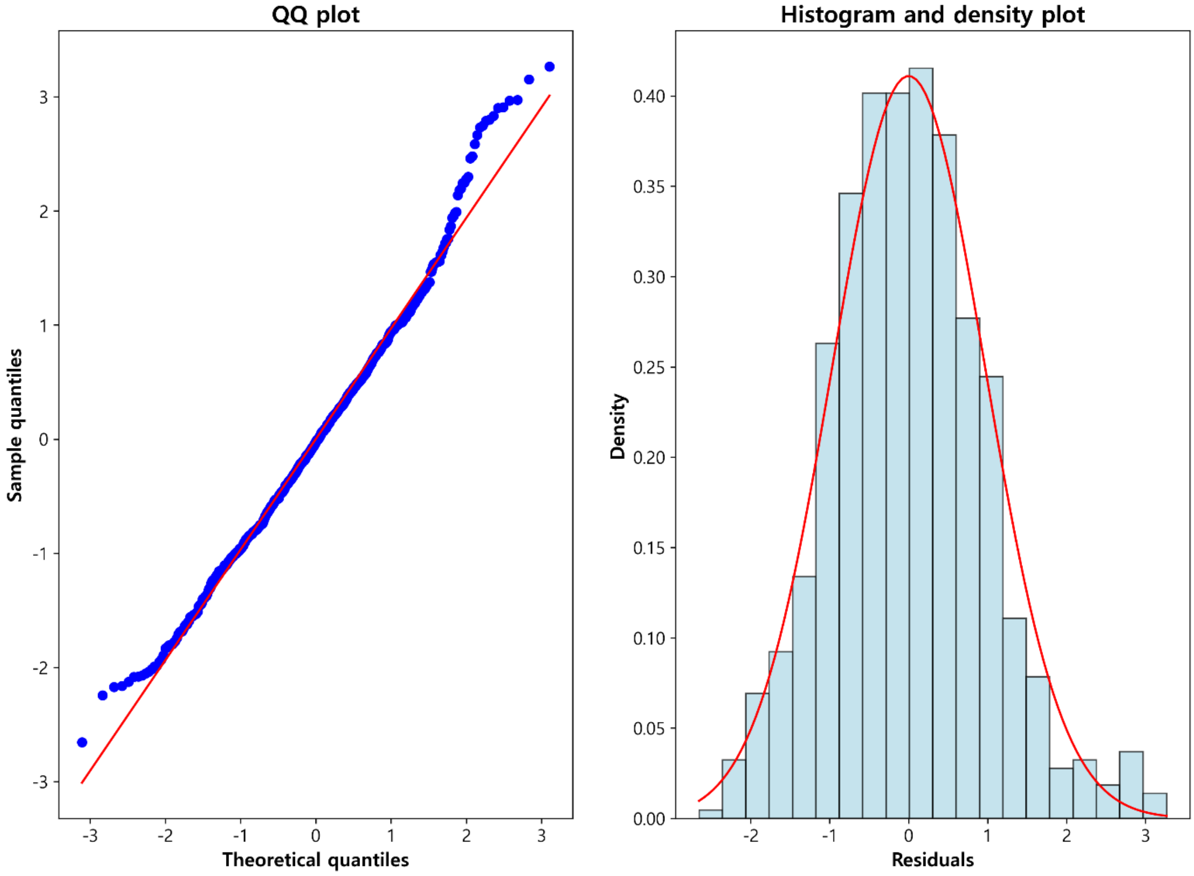

(x). Rear teat placement

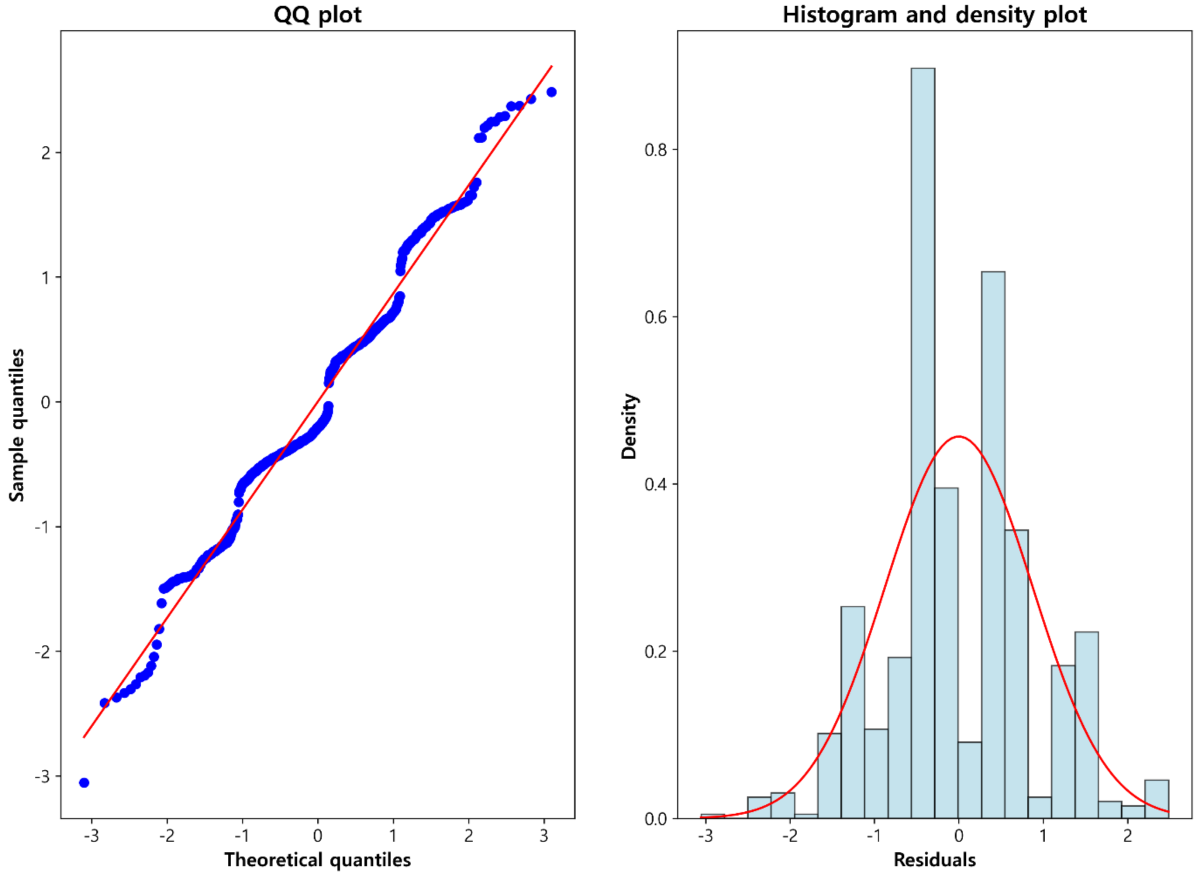

Figure S3. QQ, histogram and density plots (a-x) of residuals used in GWAS analysis for 24 body conformation traits in Korean Holstein.

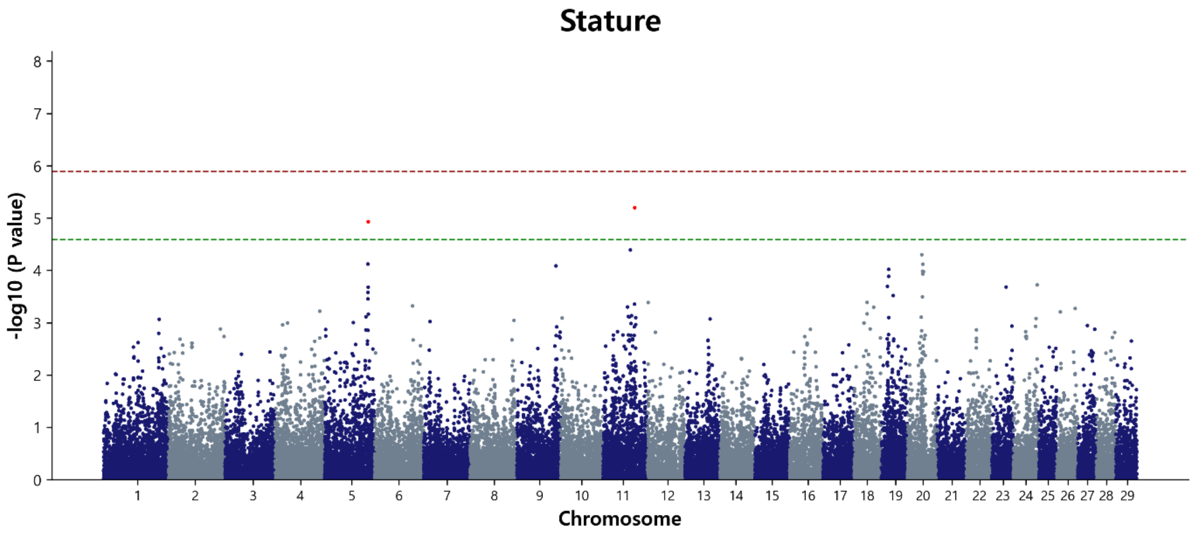

Stature
8
7
6
5
4
3
2
1
0
-log10 (P value)
1 2 3 4 5 6 7 8 9 10 11 12 13 14 15 16 17 18 19 20 21 22 23 24 25 26 27 28 29
Chromosome

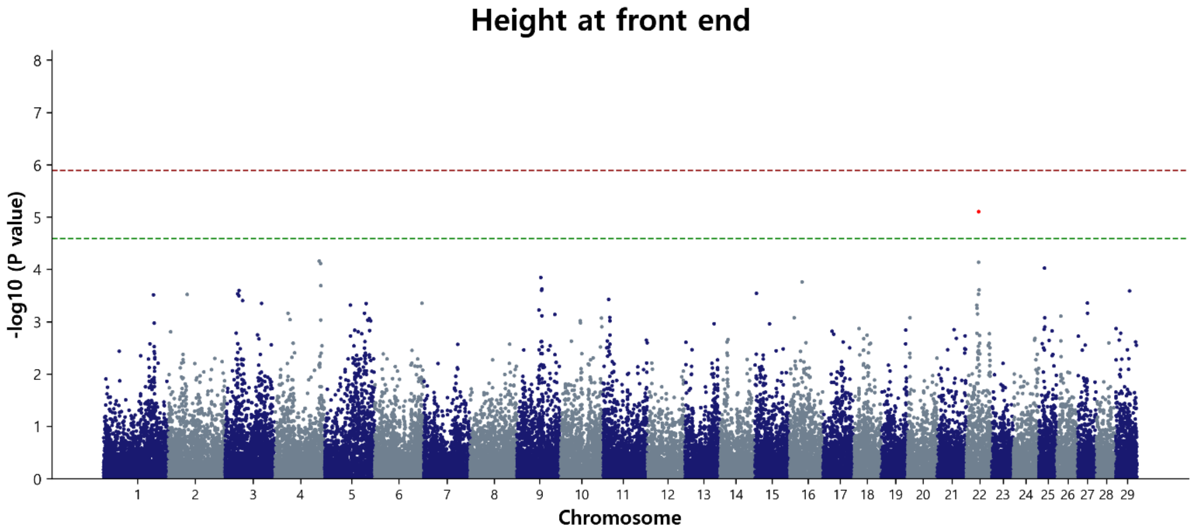

Height at front end
8
7
6
5
4
3
2
1
0
-log10 (P value)
1 2 3 4 5 6 7 8 9 10 11 12 13 14 15 16 17 18 19 20 21 22 23 24 25 26 27 28 29
Chromosome

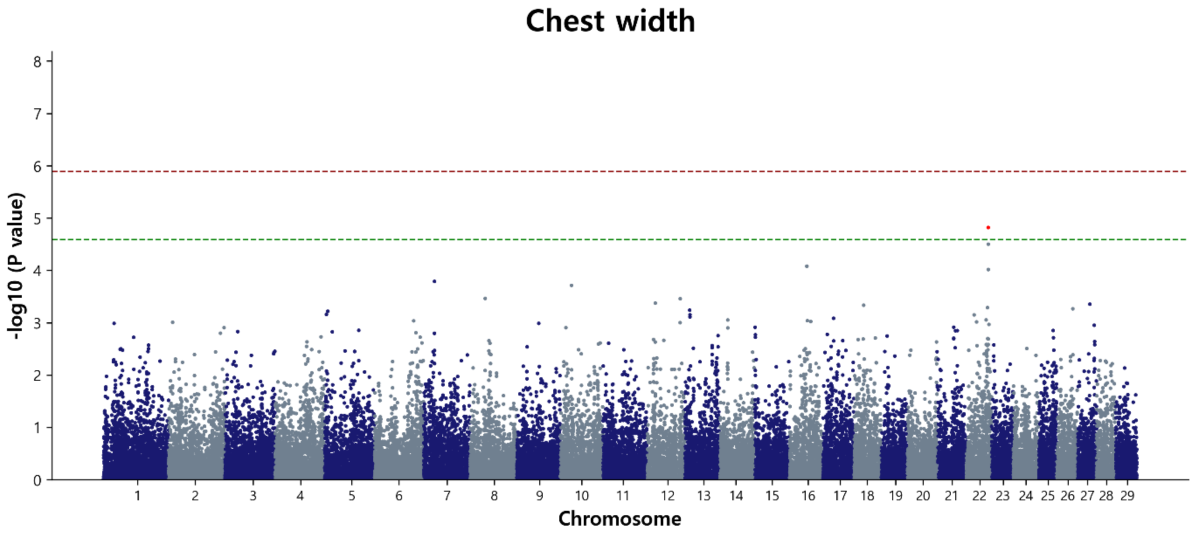
Chest width
-log10 (P value)
0
1
2
3
4
5
6
7
8
1 2 3 4 5 6 7 8 9 10 11 12 13 14 15 16 17 18 19 20 21 22 23 24 25 26 27 28 29
Chromosome

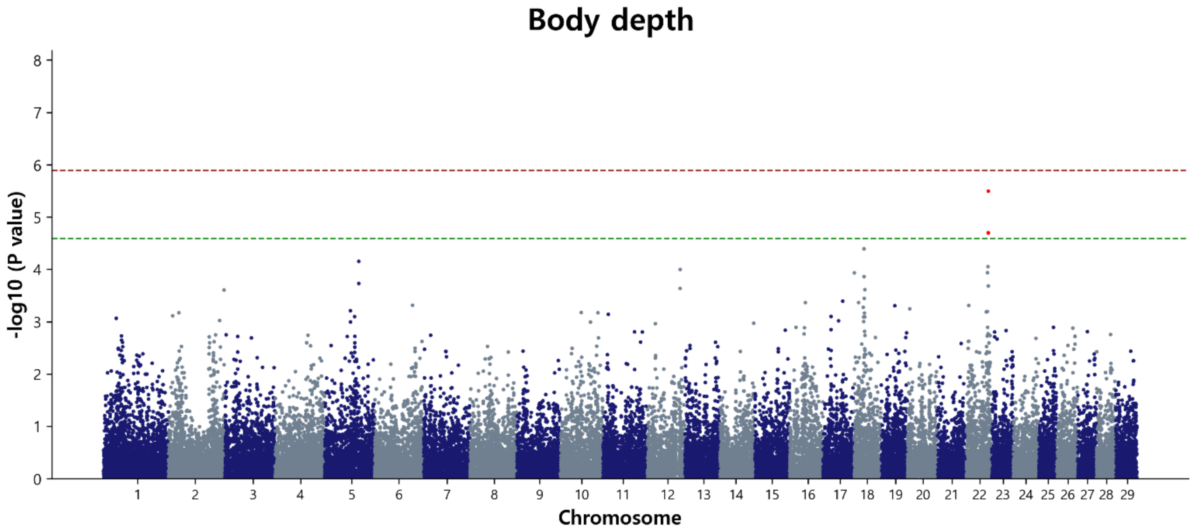
Body depth
-log10 (P value)
0
1
2
3
4
5
6
7
8
1 2 3 4 5 6 7 8 9 10 11 12 13 14 15 16 17 18 19 20 21 22 23 24 25 26 27 28 29
Chromosome

Angularity

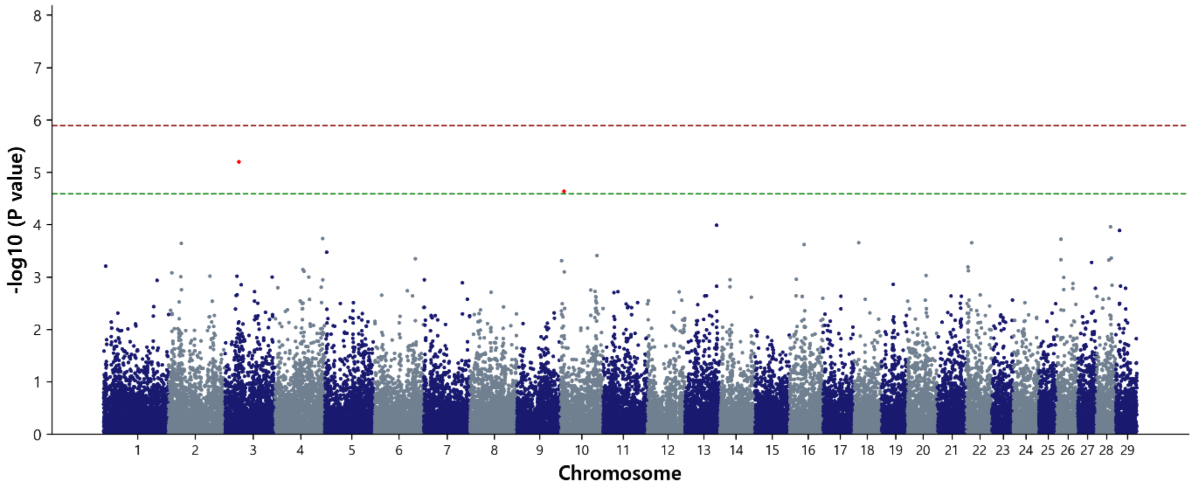

Body condition score

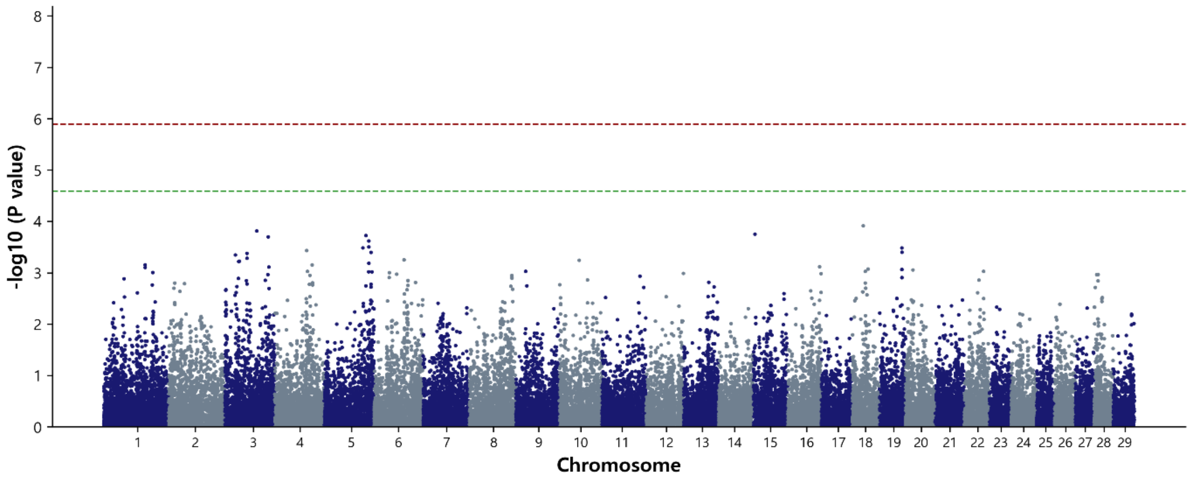

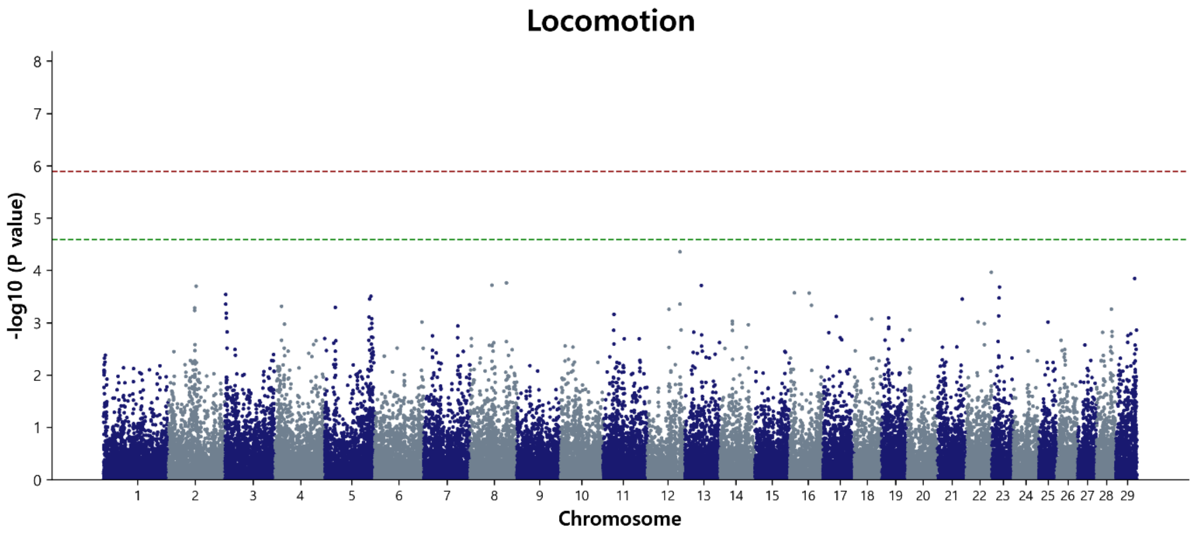

Figure S4. Manhattan plots of genome-wide -log10 (*p*-values) for body traits in Holstein cattle. The X-axis represents the 29 Bos taurus autosomes, and the Y-axis represents the -log10P values. The horizontal dashed line in green indicates the suggestive significance threshold at $p = 2.58 \times 10^{-5}$ (4.59). The genome-wide threshold (red dashed line) corresponds to the Bonferroni correction at $p = 1.29 \times 10^{-6}$ (5.89).

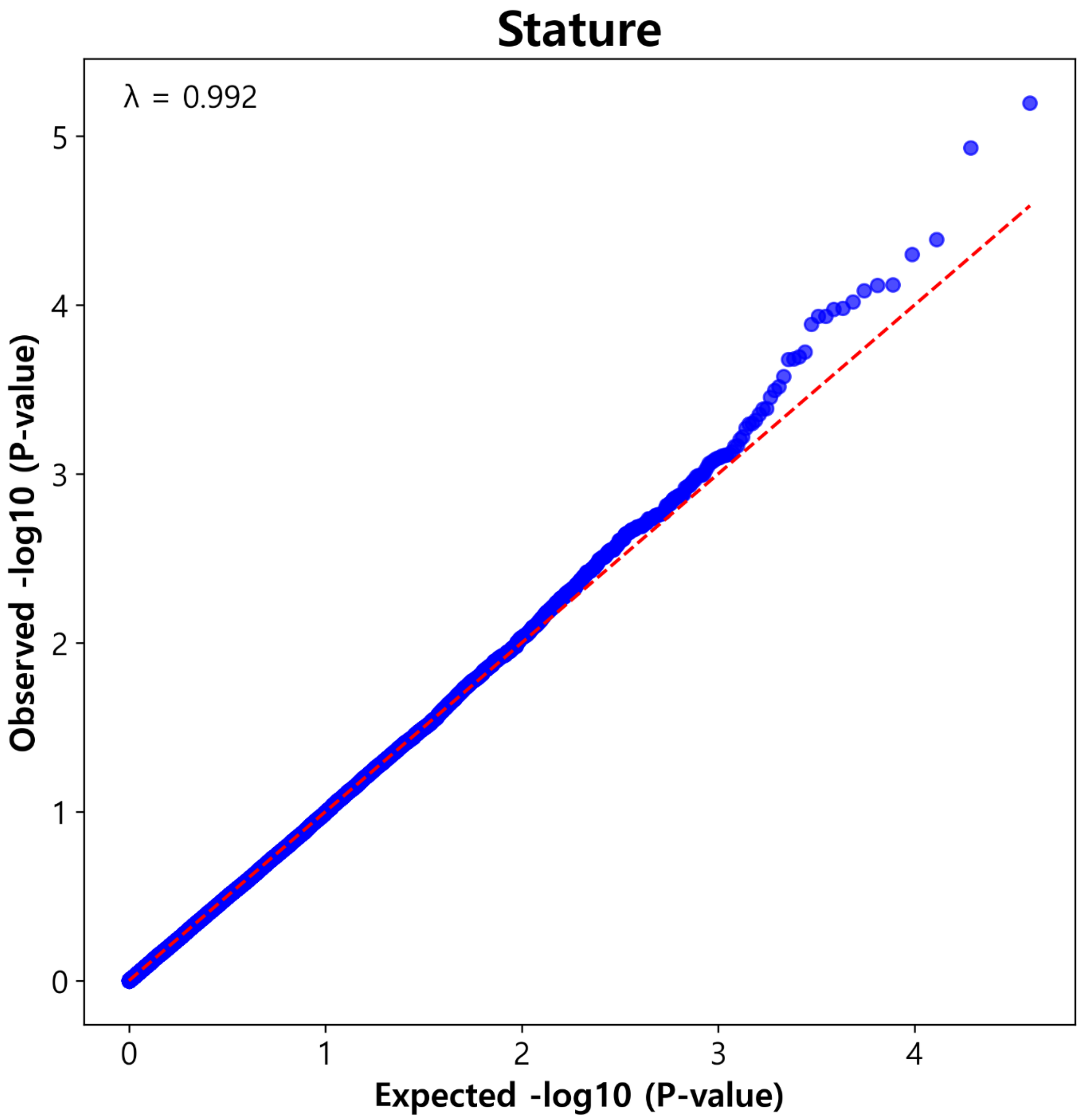
Stature
λ = 0.992
Observed -log10 (P-value)
Expected -log10 (P-value)
0
1
2
3
4
5
0
1
2
3
4

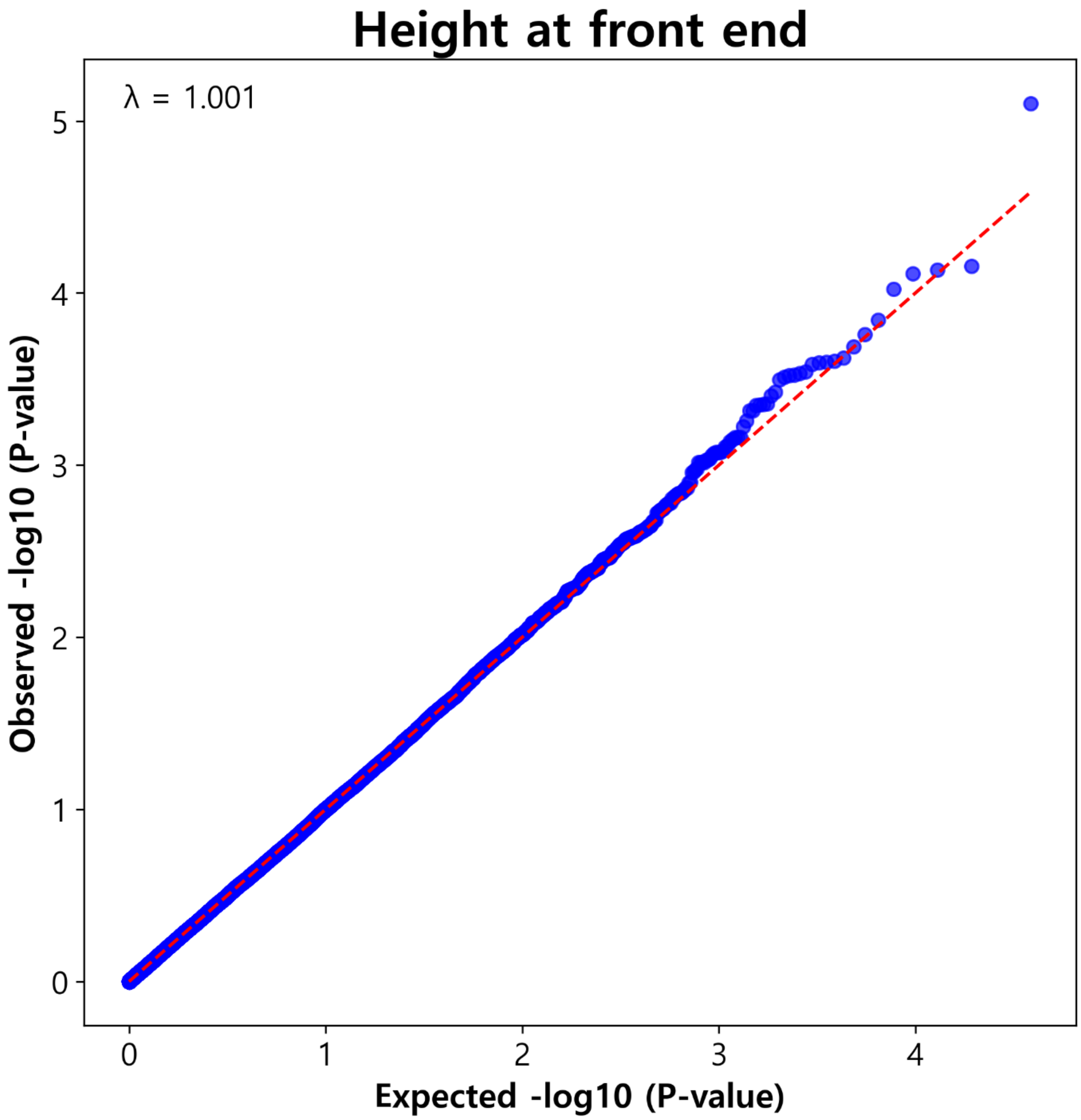
Height at front end
λ = 1.001
Observed -log10 (P-value)
Expected -log10 (P-value)
0
1
2
3
4
5

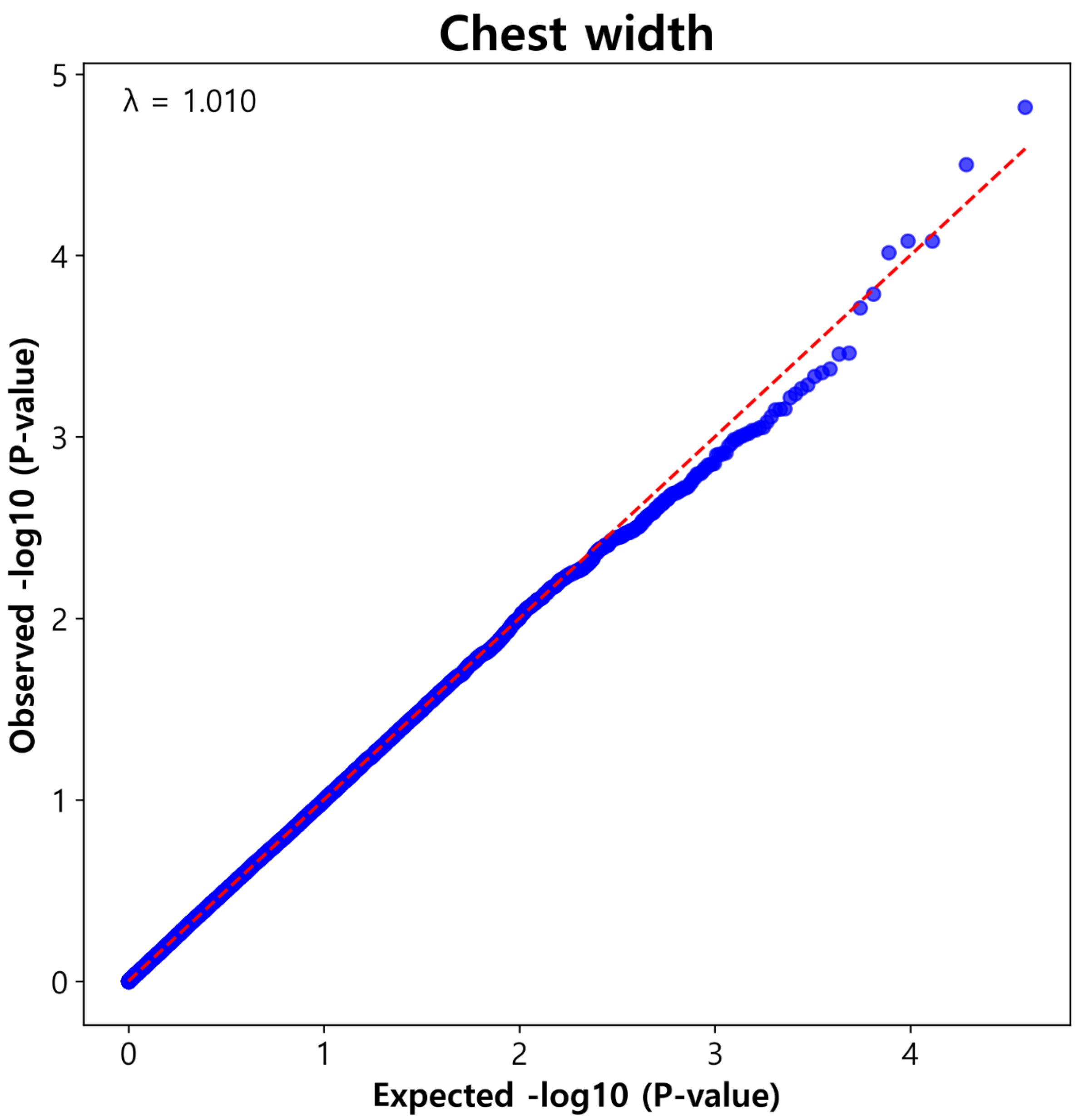

Chest width
λ = 1.010
Observed -log10 (P-value)
Expected -log10 (P-value)
0
1
2
3
4
5

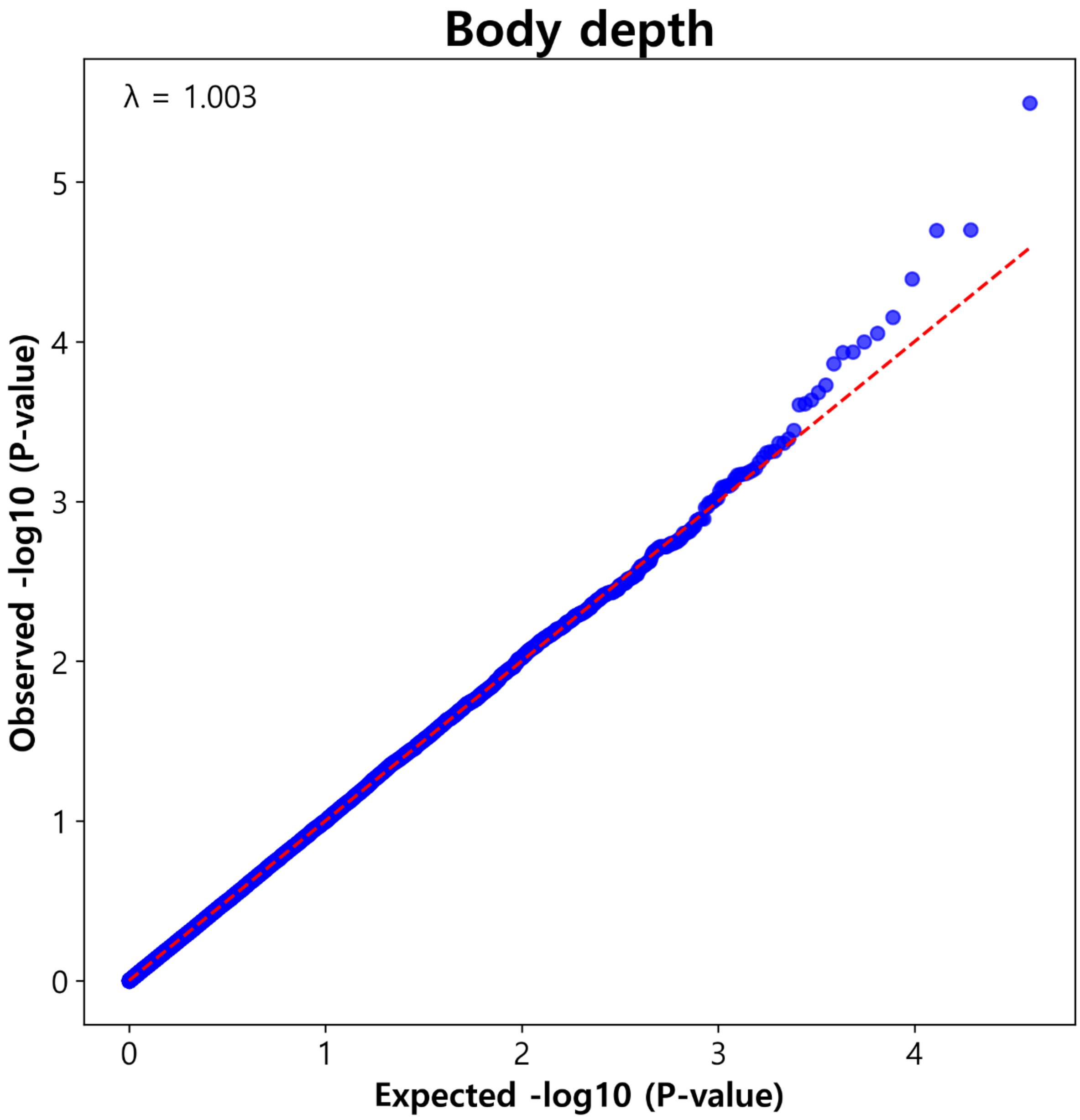

Body depth
λ = 1.003
Observed -log10 (P-value)
Expected -log10 (P-value)
0
1
2
3
4
5

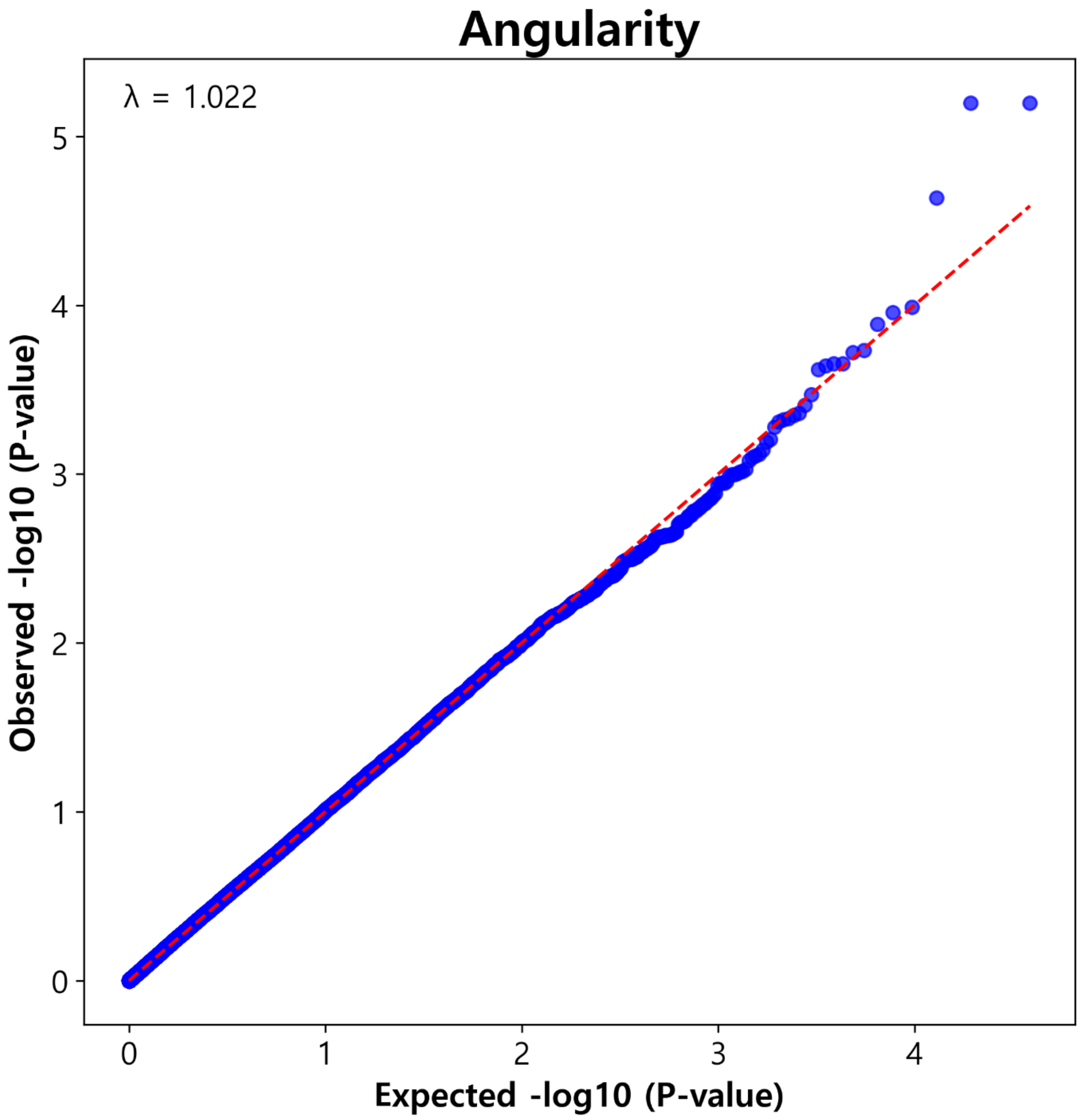
Angularity
λ = 1.022
Observed -log10 (P-value)
Expected -log10 (P-value)
0
1
2
3
4
5
0
1
2
3
4

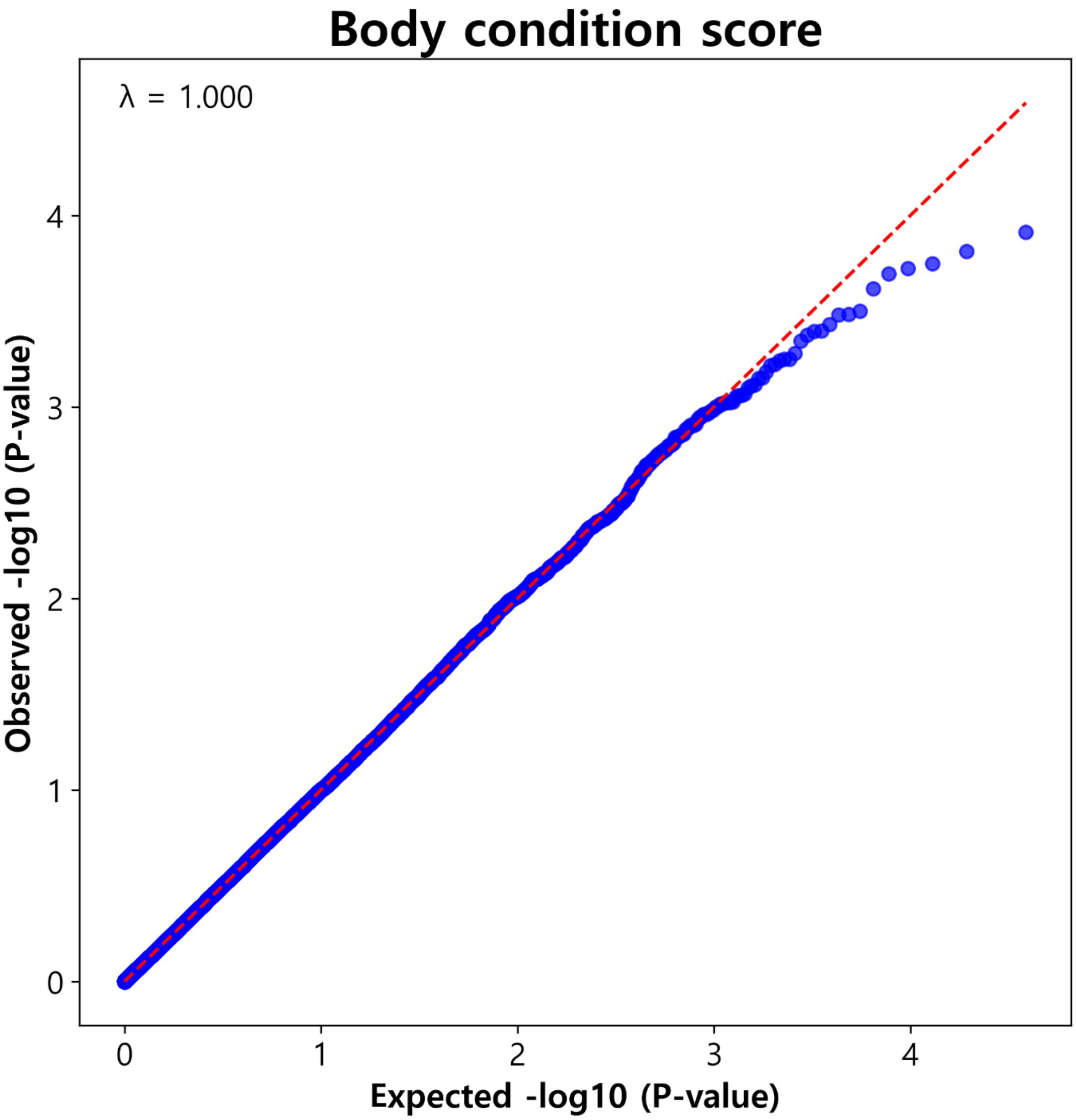

Body condition score
λ = 1.000
Observed -log10 (P-value)
Expected -log10 (P-value)
0
1
2
3
4
0
1
2
3
4

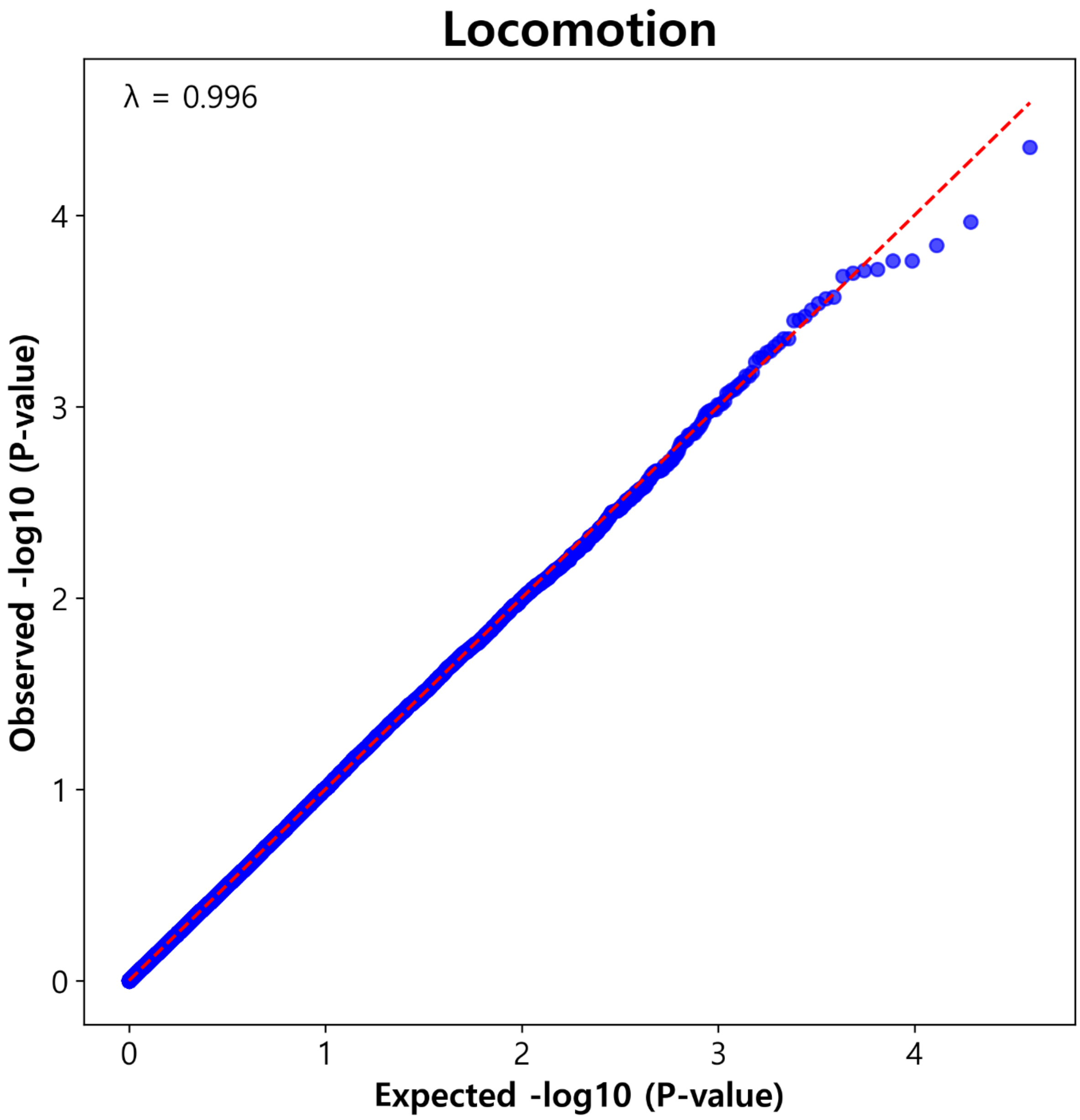

Figure S5. Quantile-quantile (Q-Q) plots and genomic inflation factor (λ) of the GWAS analysis for body traits in Korean Holstein. QQ plots showing the late separation between observed and expected *p*-values (-log10P). Genomic inflation factor (λ) is around 1 indicating that there is no population stratification.

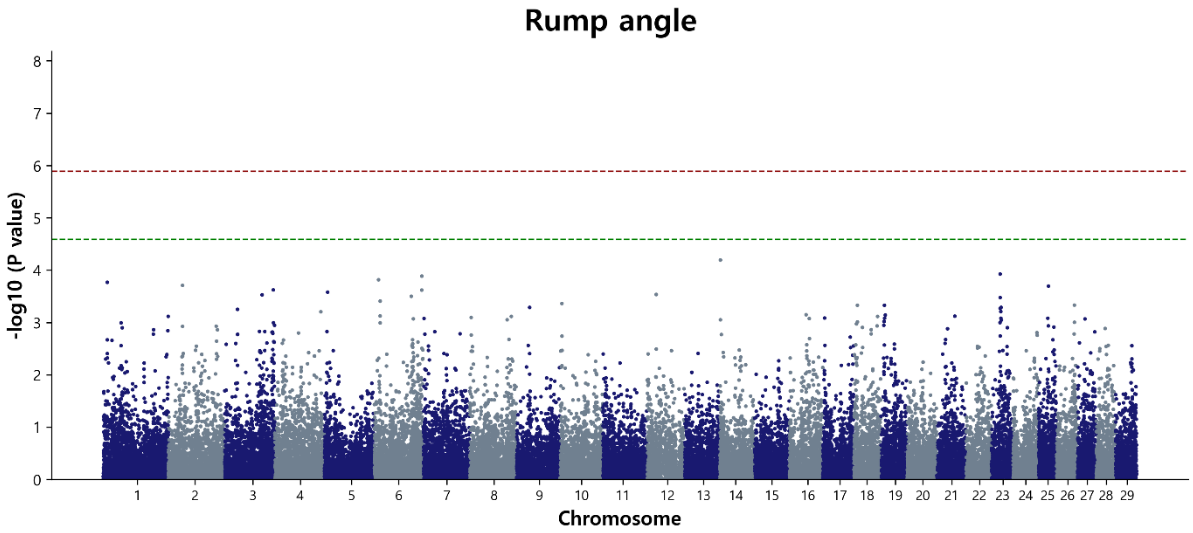
Rump angle
-log10 (P value)
0
1
2
3
4
5
6
7
8
1 2 3 4 5 6 7 8 9 10 11 12 13 14 15 16 17 18 19 20 21 22 23 24 25 26 27 28 29
Chromosome

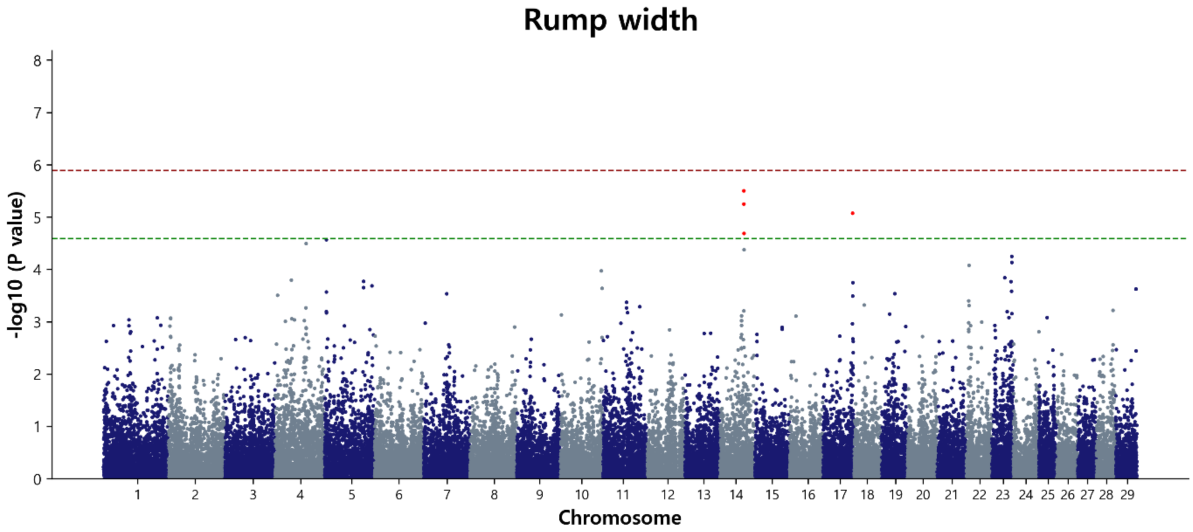
Rump width
-log10 (P value)
0
1
2
3
4
5
6
7
8
1 2 3 4 5 6 7 8 9 10 11 12 13 14 15 16 17 18 19 20 21 22 23 24 25 26 27 28 29
Chromosome

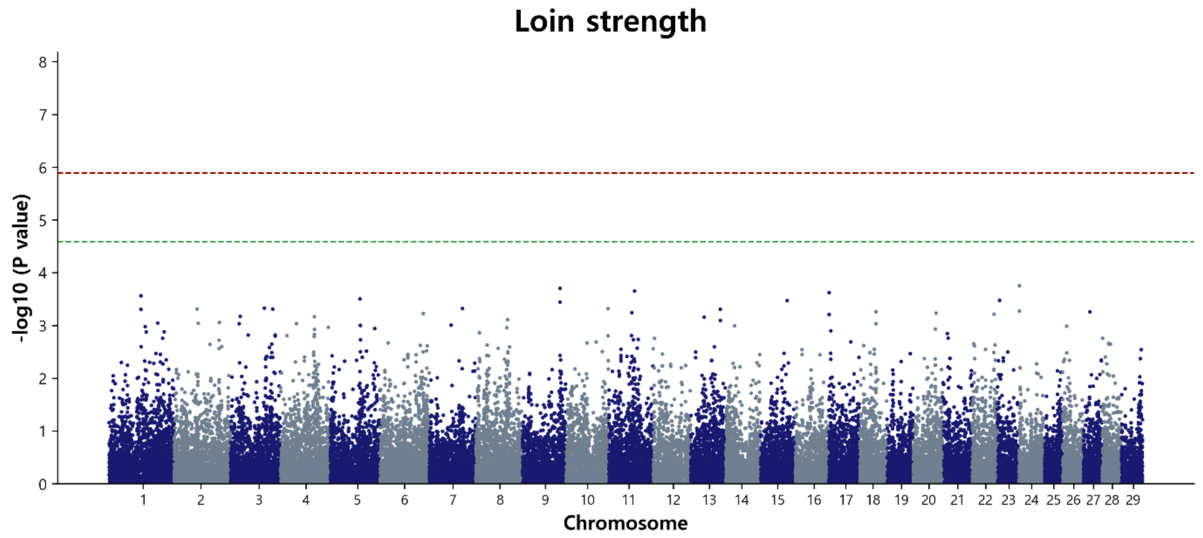

Figure S6. Manhattan plots of genome-wide -log10 (p-values) for rump traits in Holstein cattle. The X-axis represents the 29 Bos taurus autosomes, and the Y-axis represents the -log10P values. The horizontal dashed line in green indicates the suggestive significance threshold at $p = 2.58 \times 10^{-5}$ (4.59). The genome-wide threshold (red dashed line) corresponds to the Bonferroni correction at $p = 1.29 \times 10^{-6}$ (5.89).

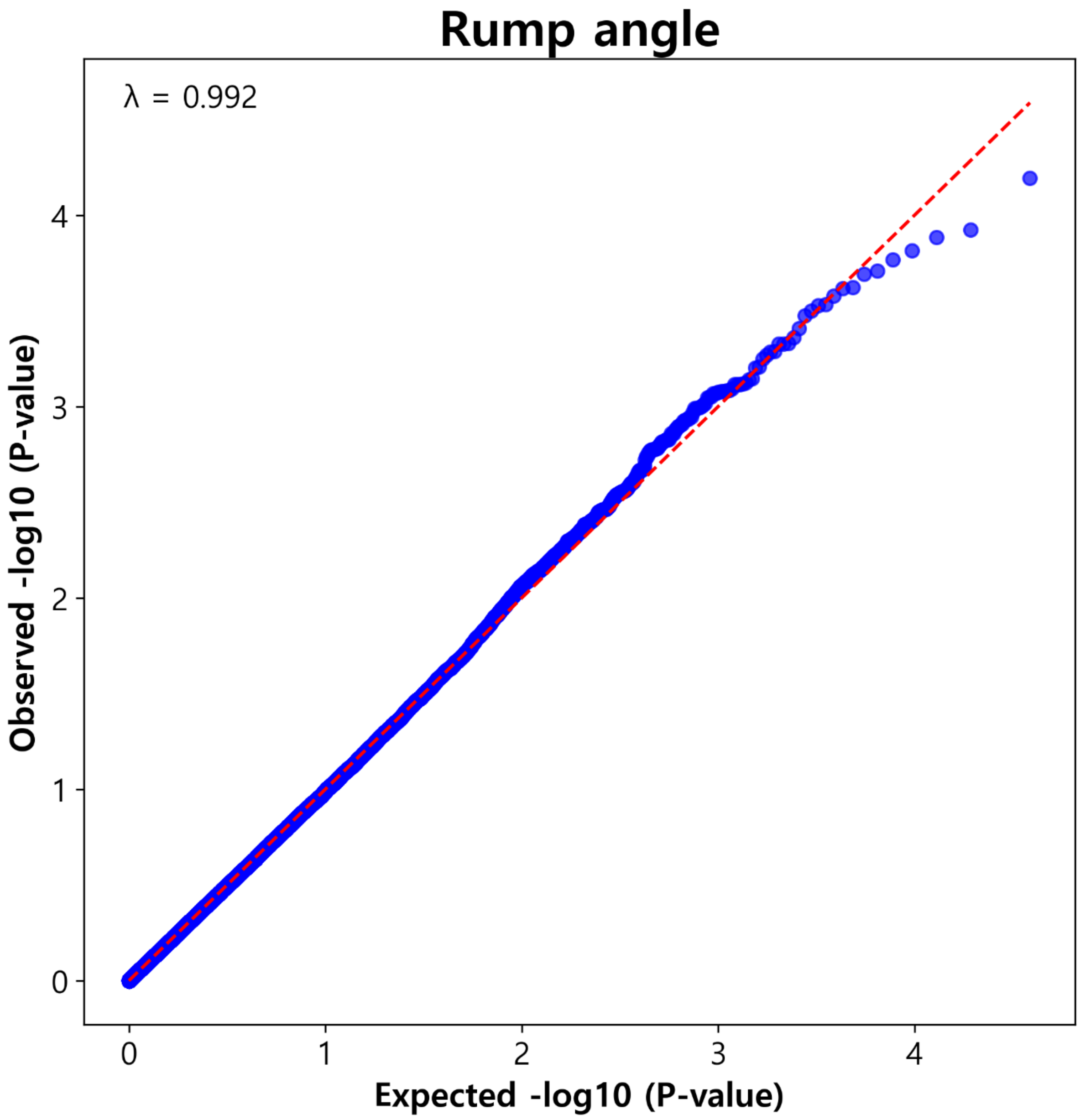

Rump angle
λ = 0.992
Observed -log10 (P-value)
Expected -log10 (P-value)
0
1
2
3
4

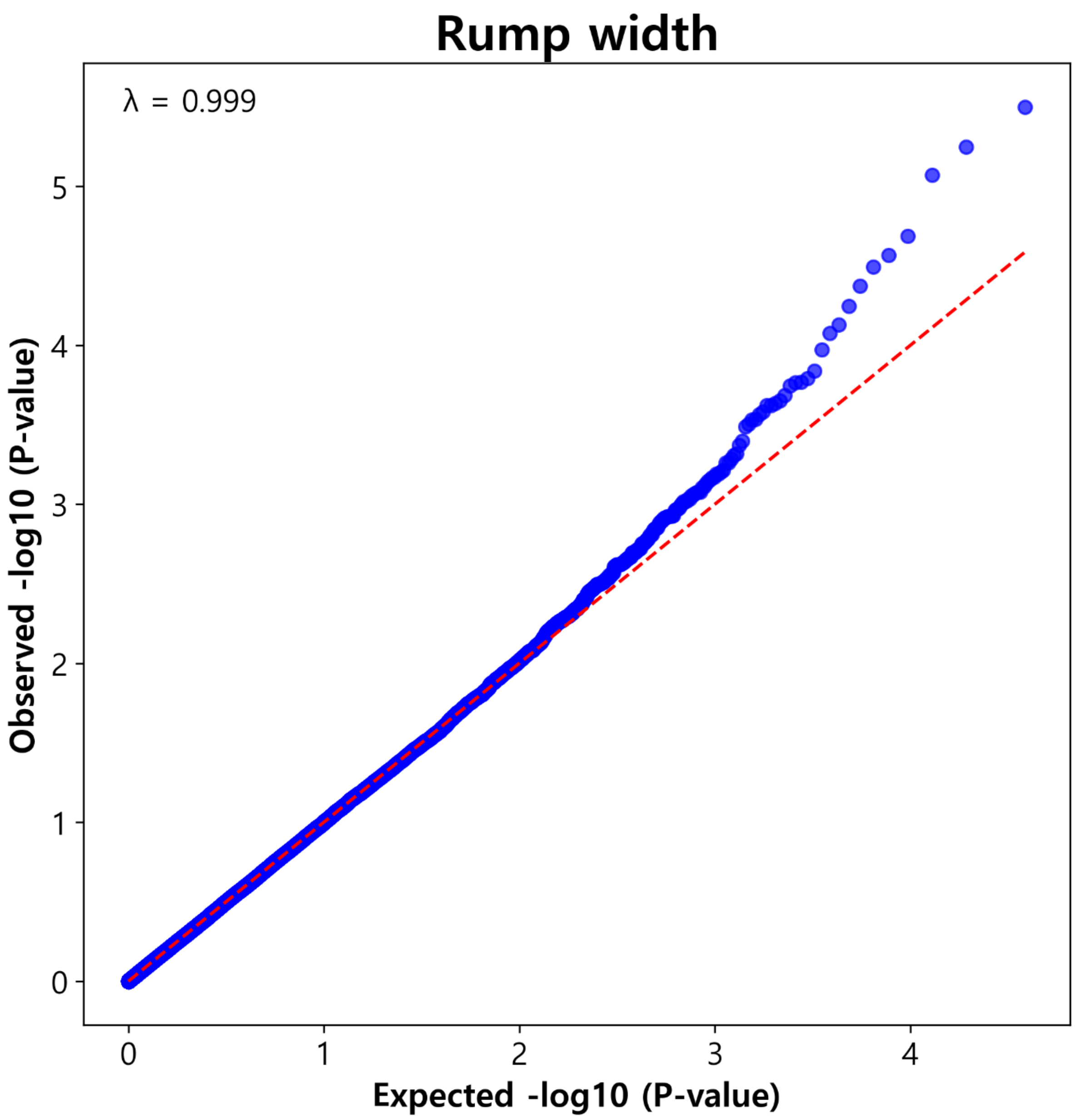

Rump width
λ = 0.999
Observed -log10 (P-value)
Expected -log10 (P-value)
0
1
2
3
4
5

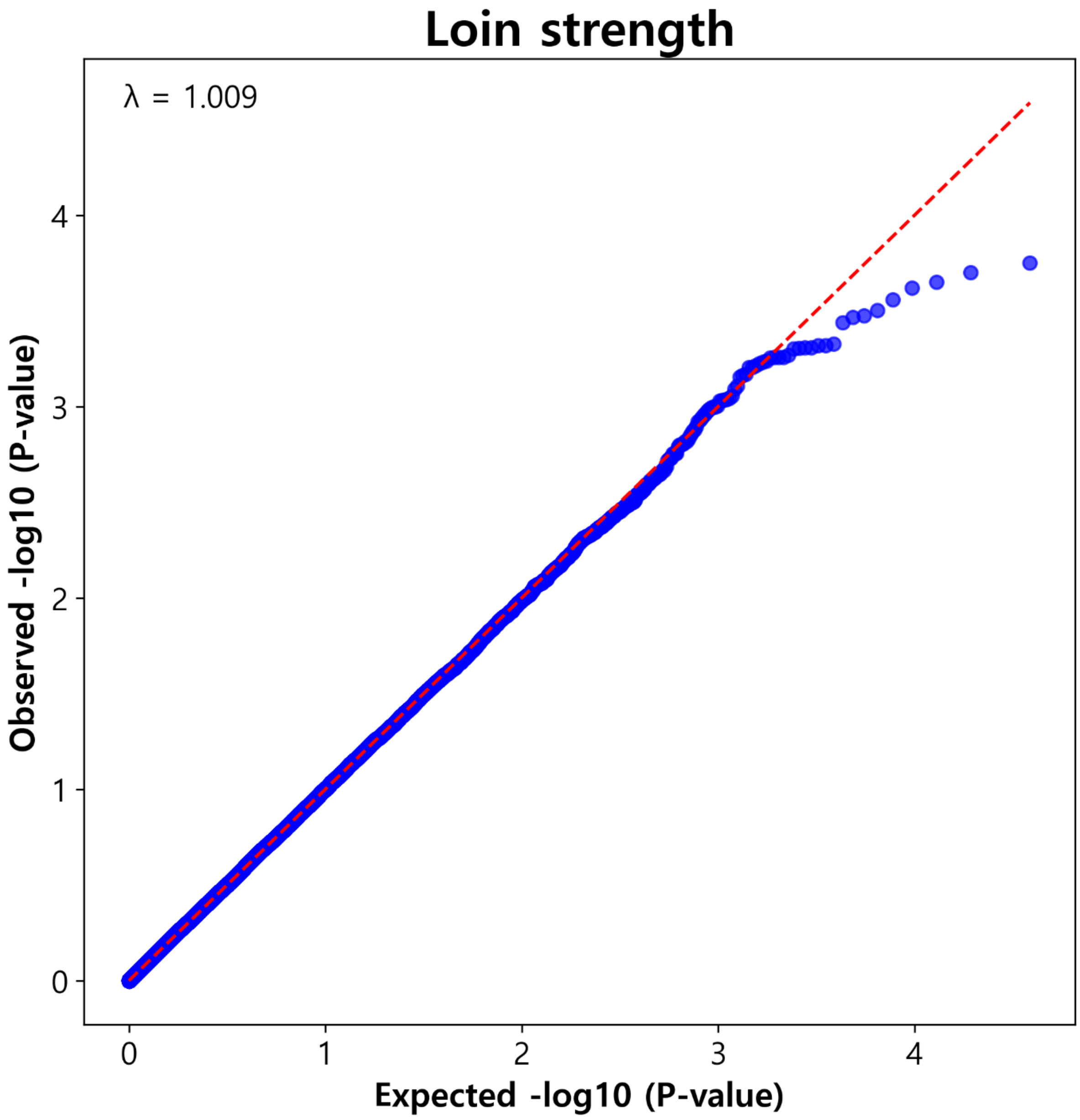

Figure S7. Quantile-quantile (Q-Q) plots and genomic inflation factor (λ) of the GWAS analysis for rump traits in Korean Holstein. QQ plots showing the late separation between observed and expected *p*-values (-log10P). Genomic inflation factor (λ) is around 1 indicating that there is no population stratification.

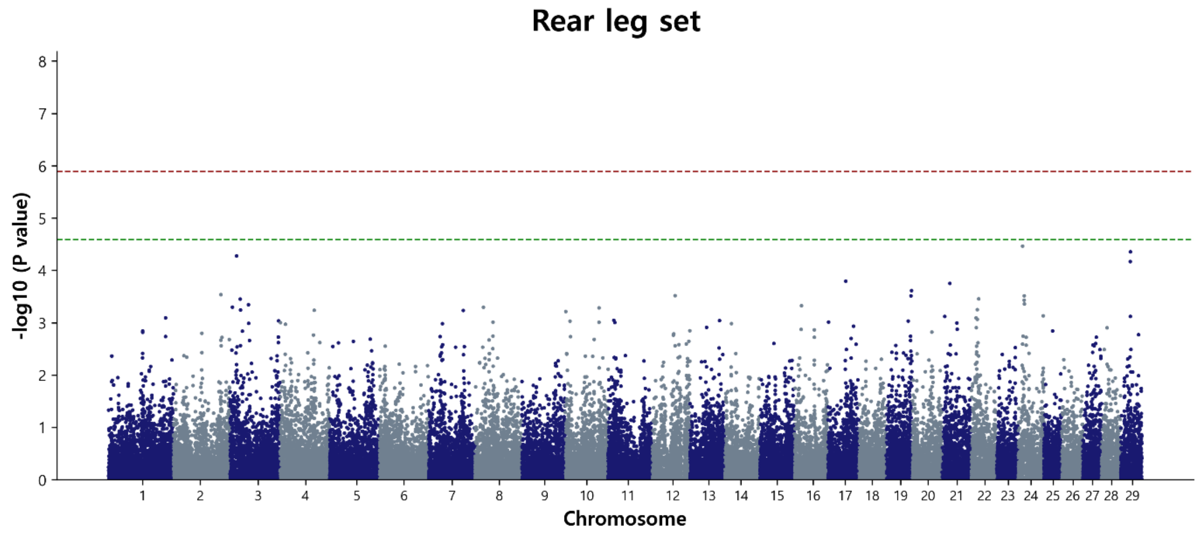
Rear leg set
-log10 (P value)
0
1
2
3
4
5
6
7
8
1 2 3 4 5 6 7 8 9 10 11 12 13 14 15 16 17 18 19 20 21 22 23 24 25 26 27 28 29
Chromosome

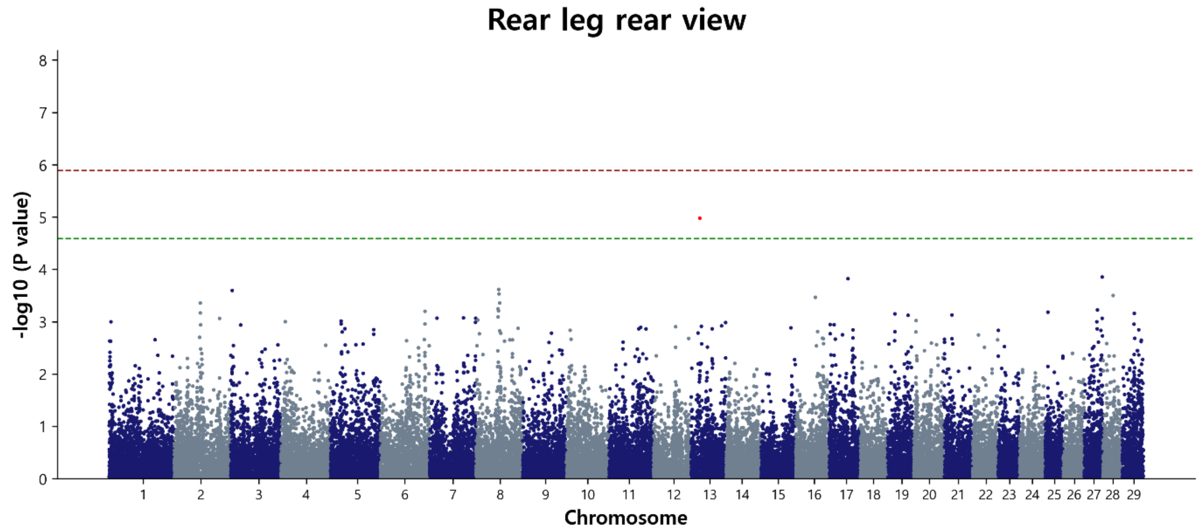
Rear leg rear view
-log10 (P value)
0
1
2
3
4
5
6
7
8
1 2 3 4 5 6 7 8 9 10 11 12 13 14 15 16 17 18 19 20 21 22 23 24 25 26 27 28 29
Chromosome

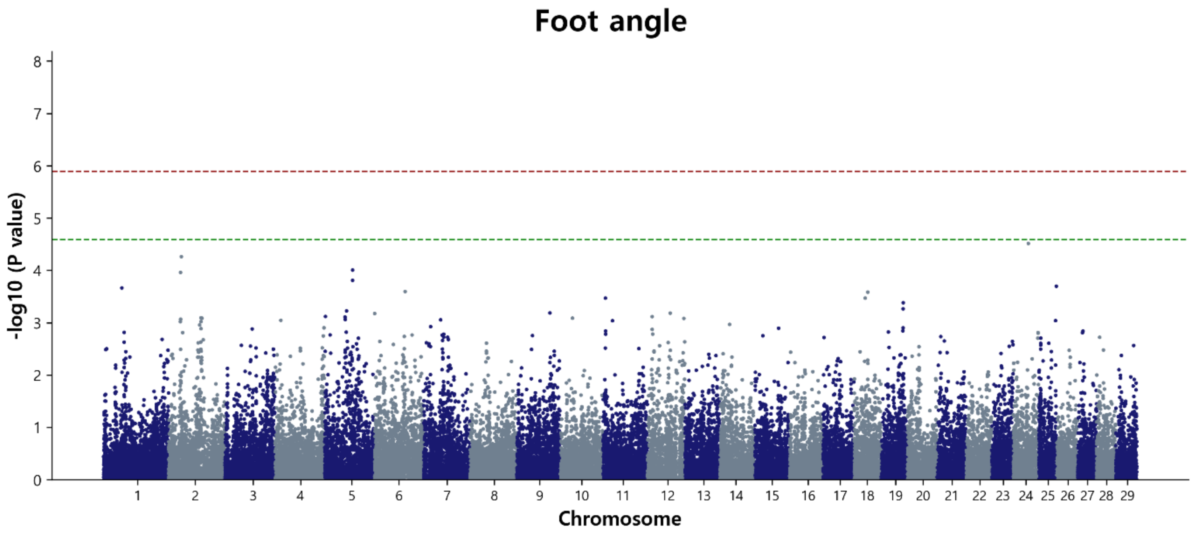
Foot angle
-log10 (P value)
0
1
2
3
4
5
6
7
8
Chromosome
1 2 3 4 5 6 7 8 9 10 11 12 13 14 15 16 17 18 19 20 21 22 23 24 25 26 27 28 29

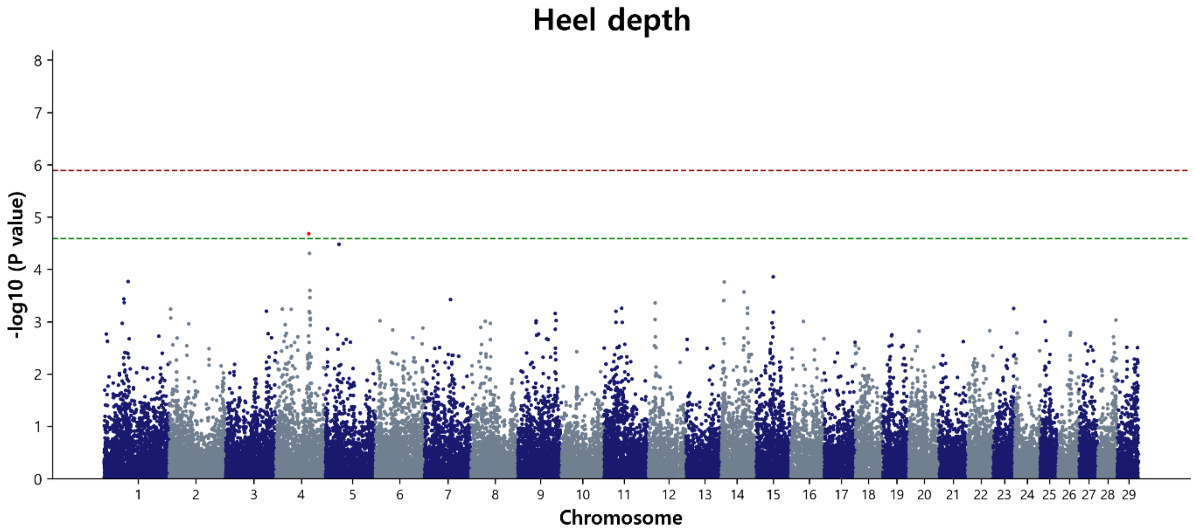
Heel depth
-log10 (P value)
0
1
2
3
4
5
6
7
8
Chromosome
1 2 3 4 5 6 7 8 9 10 11 12 13 14 15 16 17 18 19 20 21 22 23 24 25 26 27 28 29

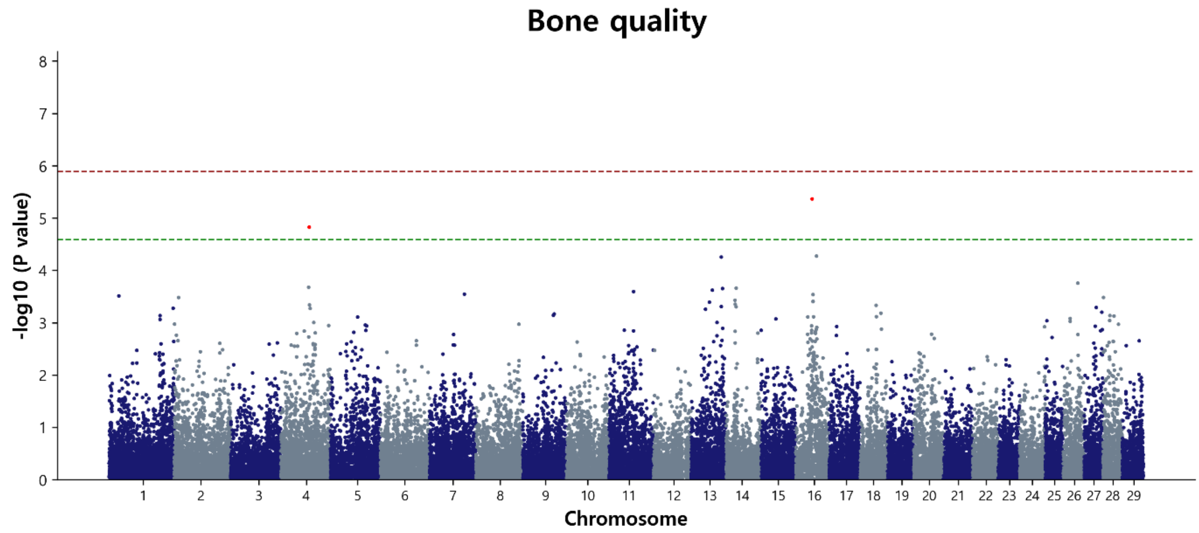

Figure S8. Manhattan plots of genome-wide -log10 (*p*-values) for feet and leg traits in Holstein cattle. The X-axis represents the 29 Bos taurus autosomes, and the Y-axis represents the -log10P values. The horizontal dashed line in green indicates the suggestive significance threshold at $p = 2.58 \times 10^{-5}$ (4.59). The genome-wide threshold (red dashed line) corresponds to the Bonferroni correction at $p = 1.29 \times 10^{-6}$ (5.89).

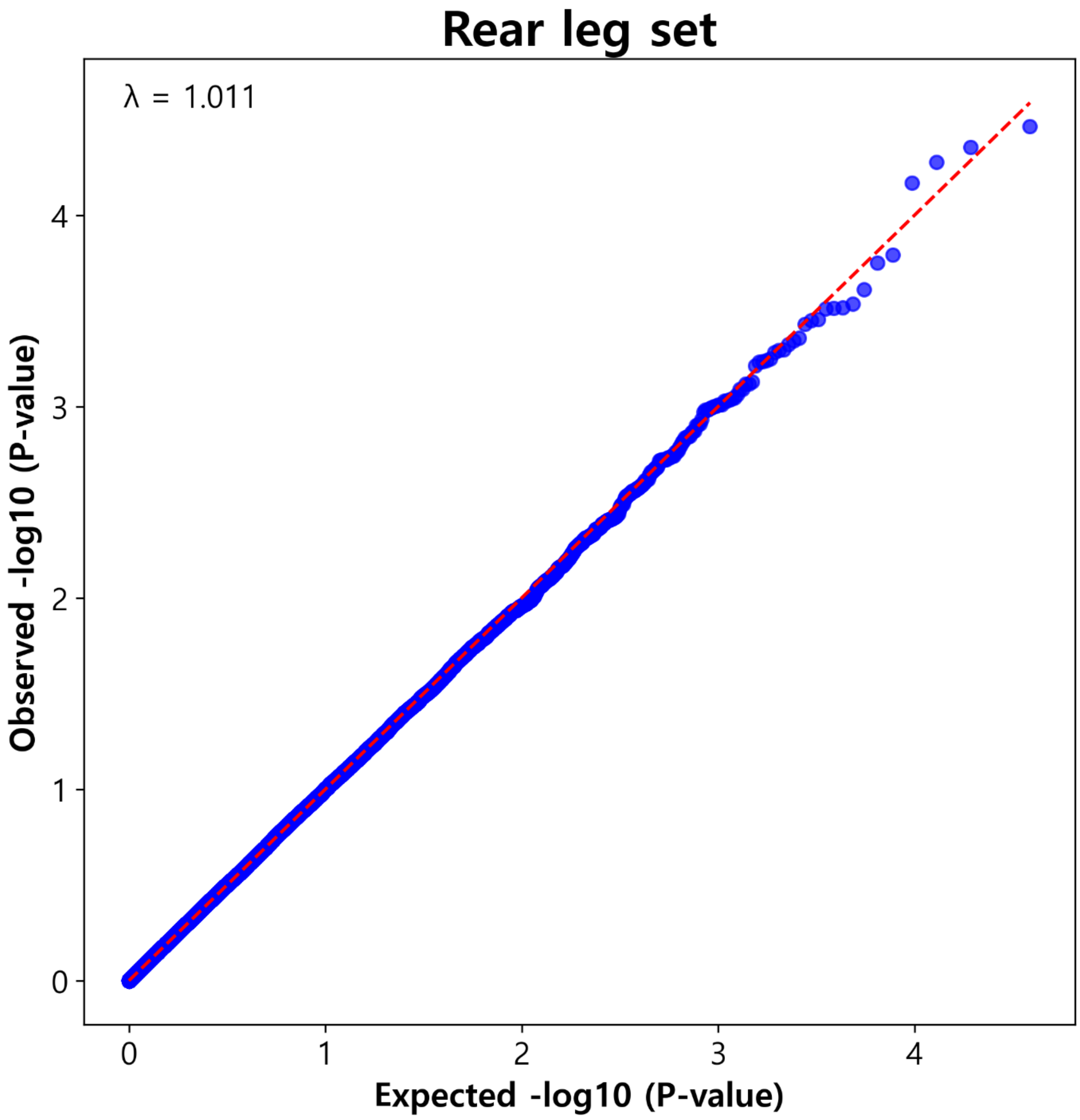

Rear leg set
λ = 1.011
Observed -log10 (P-value)
Expected -log10 (P-value)
0
1
2
3
4

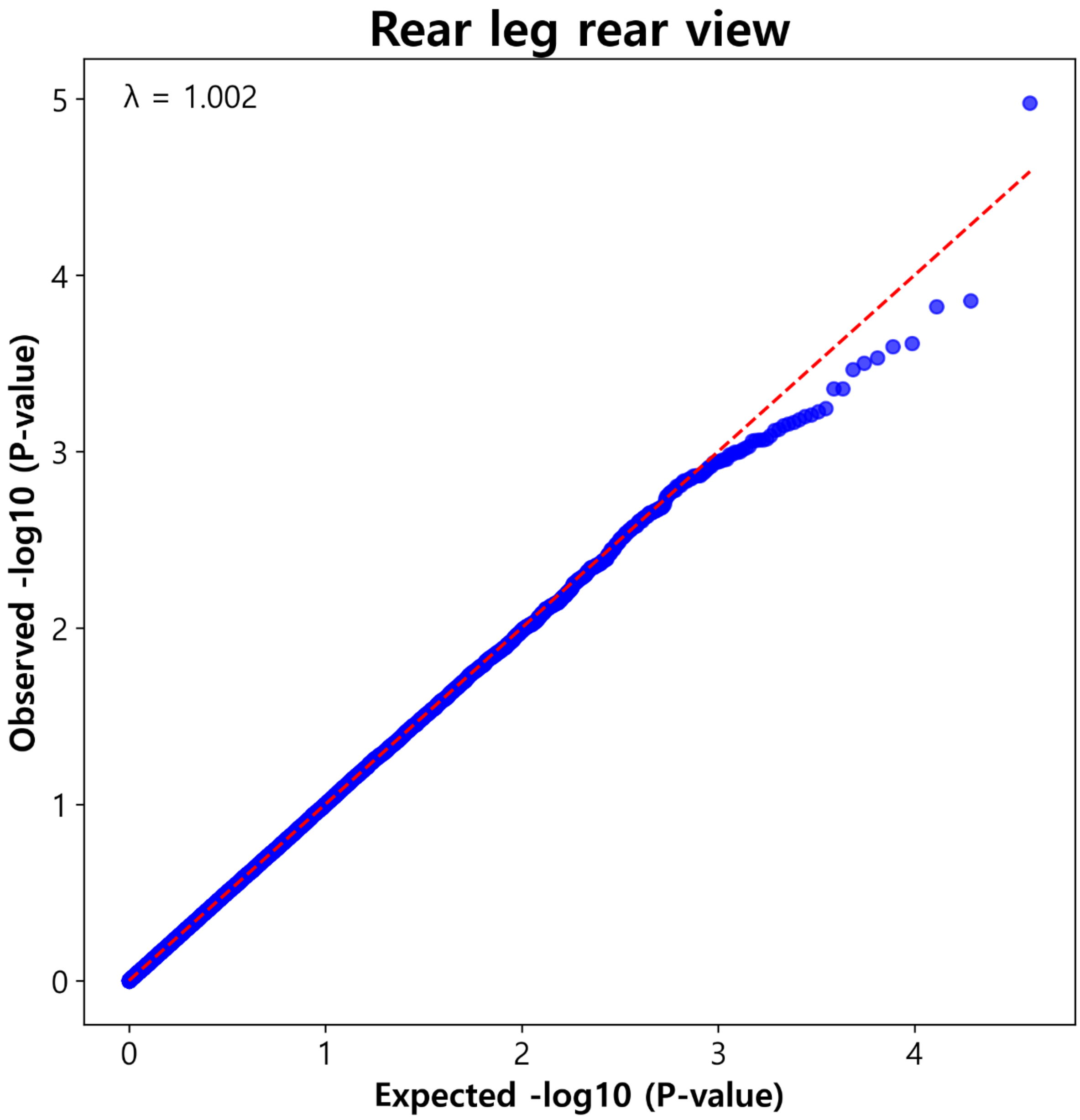

Rear leg rear view
λ = 1.002
Observed -log10 (P-value)
Expected -log10 (P-value)
0
1
2
3
4
5

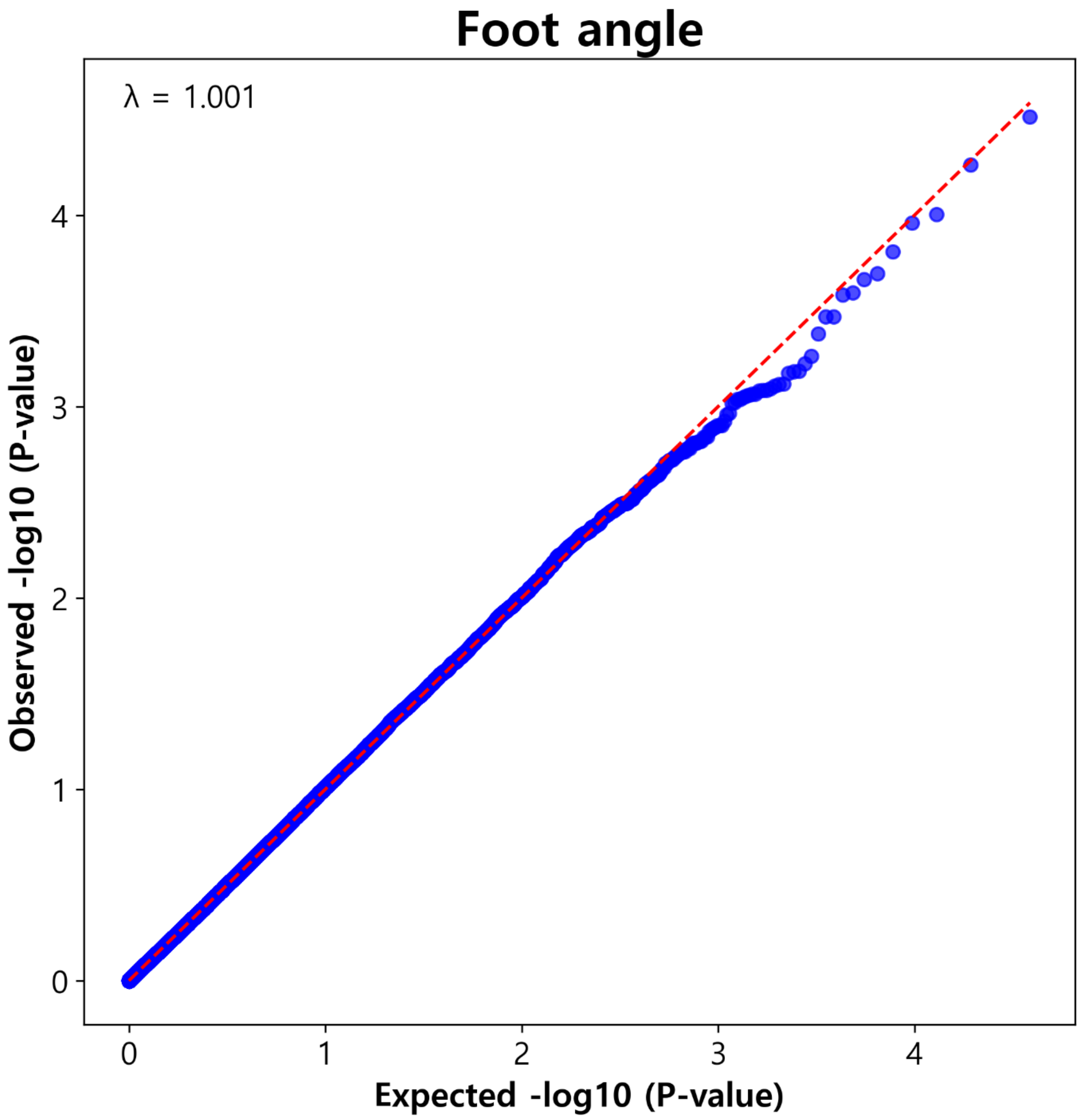
Foot angle
λ = 1.001
Observed -log10 (P-value)
Expected -log10 (P-value)
0
1
2
3
4

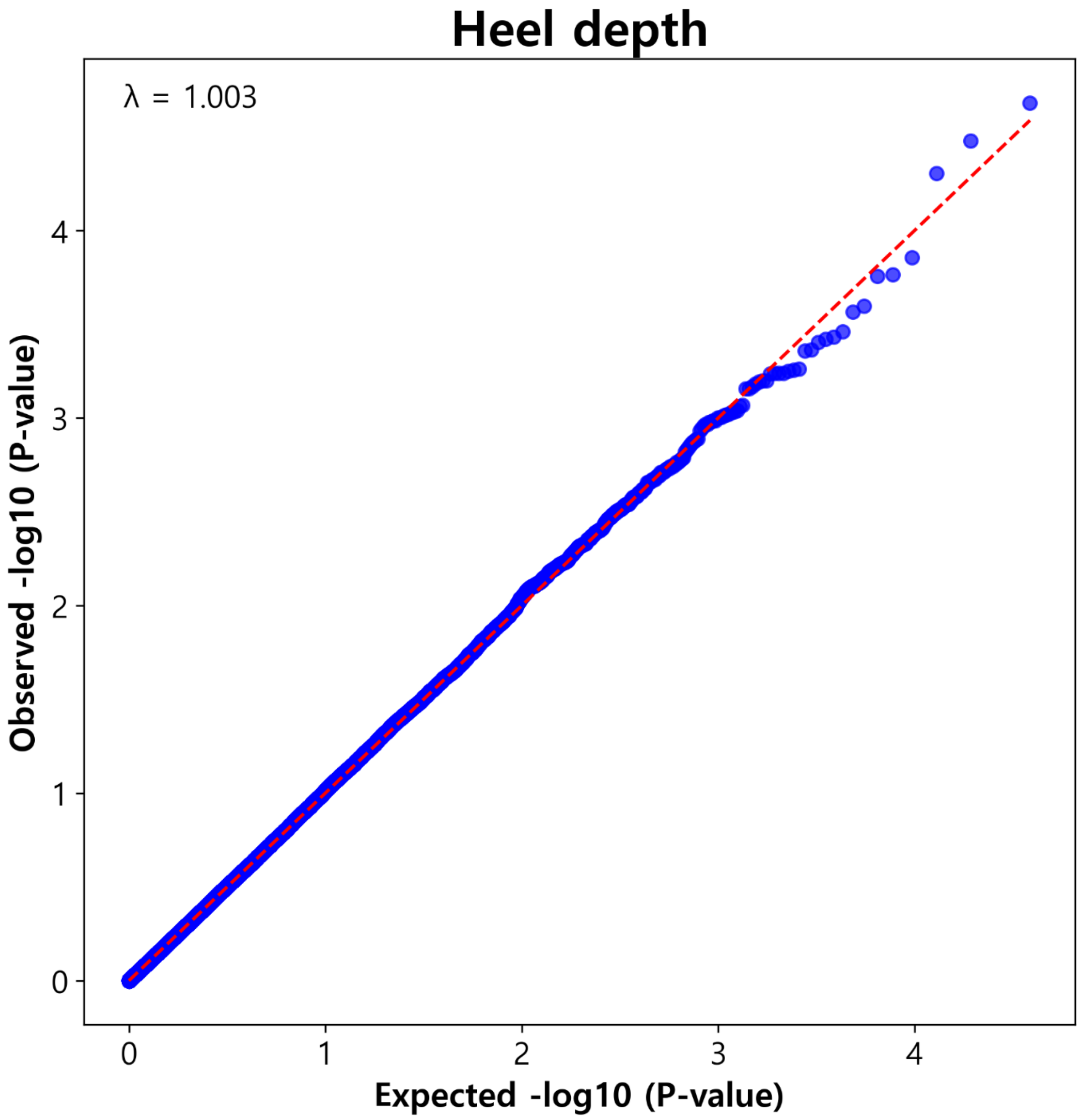
Heel depth
λ = 1.003
Observed -log10 (P-value)
Expected -log10 (P-value)
0
1
2
3
4

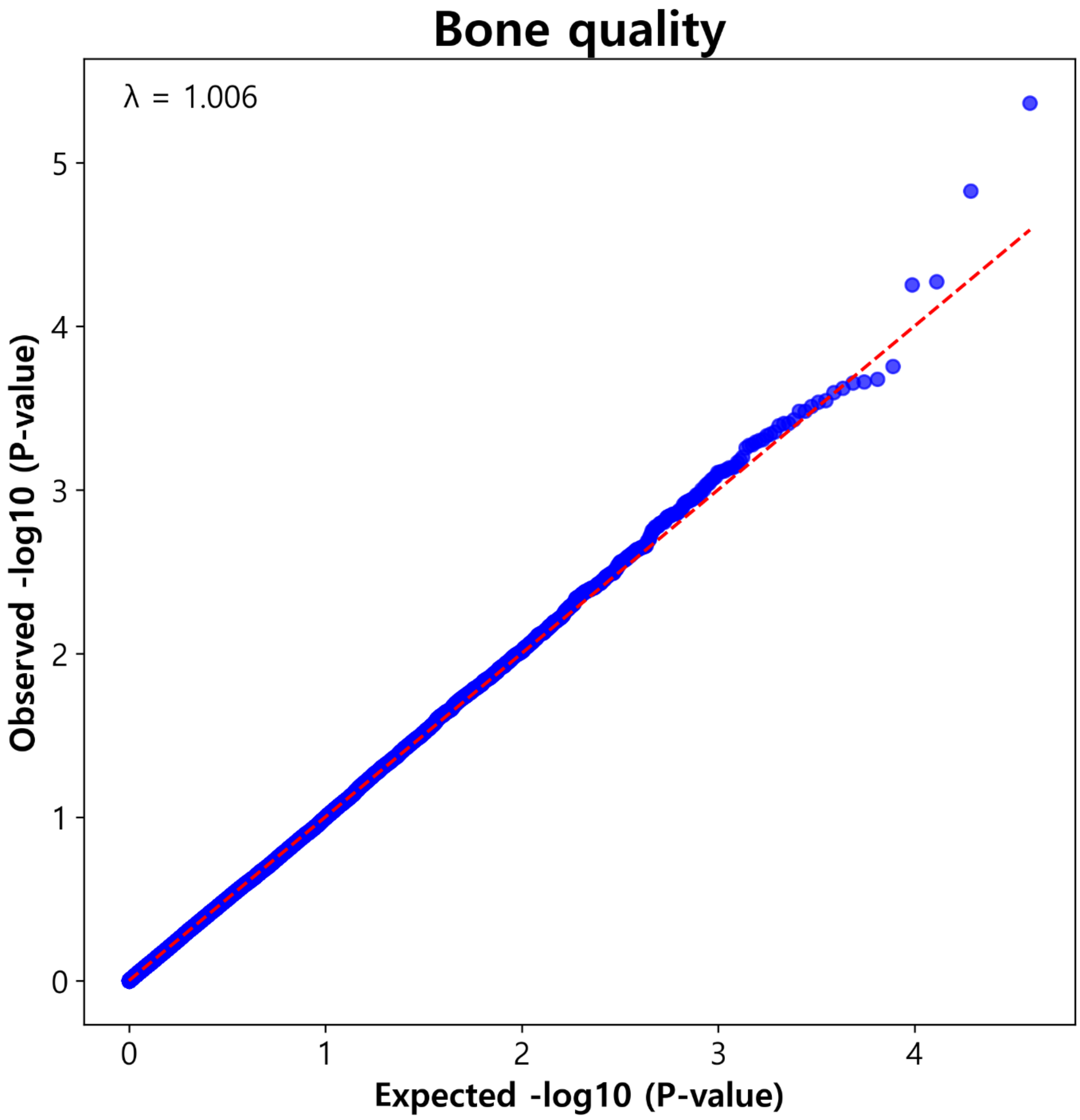

Figure S9. Quantile-quantile (Q-Q) plots and genomic inflation factor (λ) of the GWAS analysis for feet and leg traits in Korean Holstein. QQ plots showing the late separation between observed and expected *p*-values (-log10P). Genomic inflation factor (λ) is around 1 indicating that there is no population stratification.

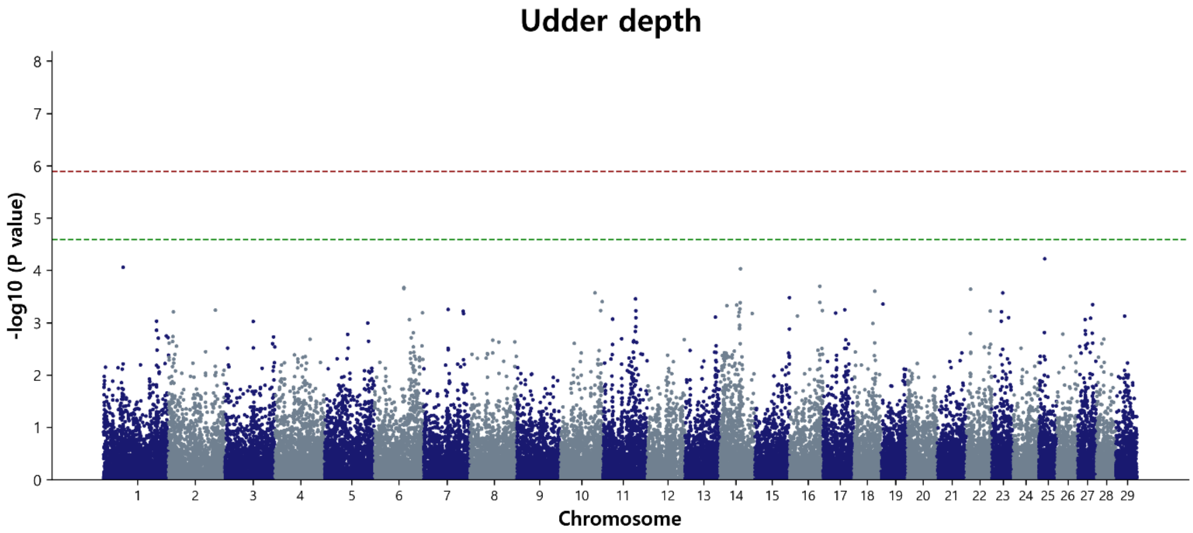
Udder depth
-log10 (P value)
0
1
2
3
4
5
6
7
8
1 2 3 4 5 6 7 8 9 10 11 12 13 14 15 16 17 18 19 20 21 22 23 24 25 26 27 28 29
Chromosome

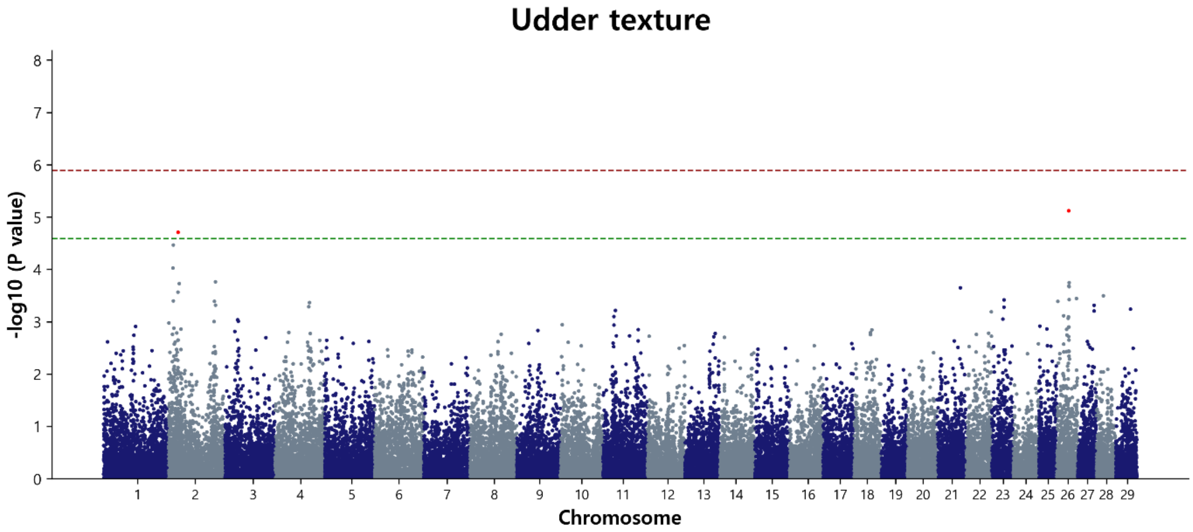
Udder texture
-log10 (P value)
0
1
2
3
4
5
6
7
8
1 2 3 4 5 6 7 8 9 10 11 12 13 14 15 16 17 18 19 20 21 22 23 24 25 26 27 28 29
Chromosome

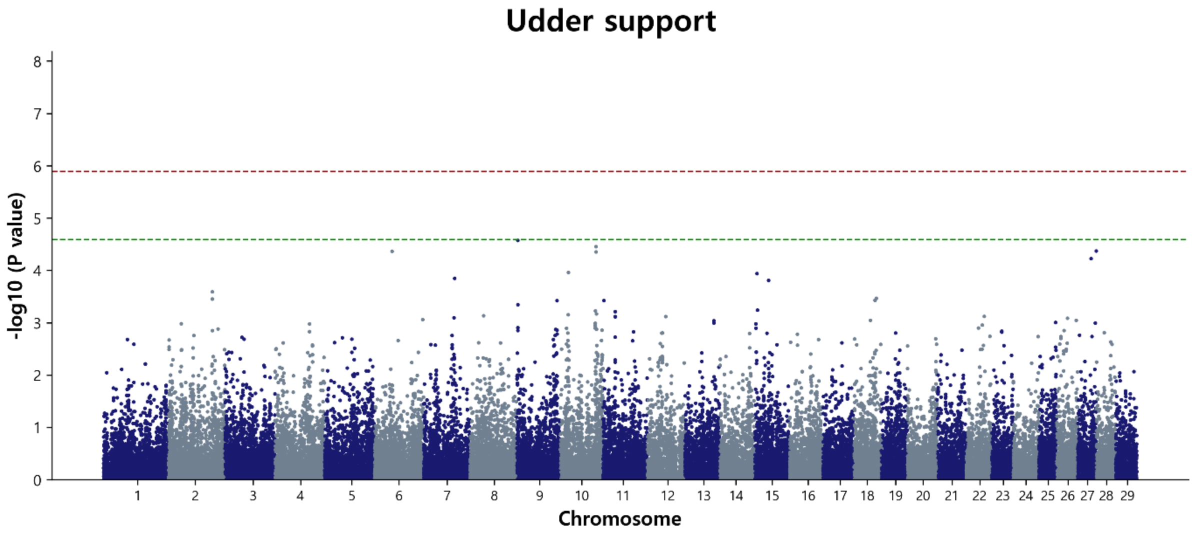
Udder support
-log10 (P value)
Chromosome
0
1
2
3
4
5
6
7
8
1
2
3
4
5
6
7
8
9
10
11
12
13
14
15
16
17
18
19
20
21
22
23
24
25
26
27
28
29

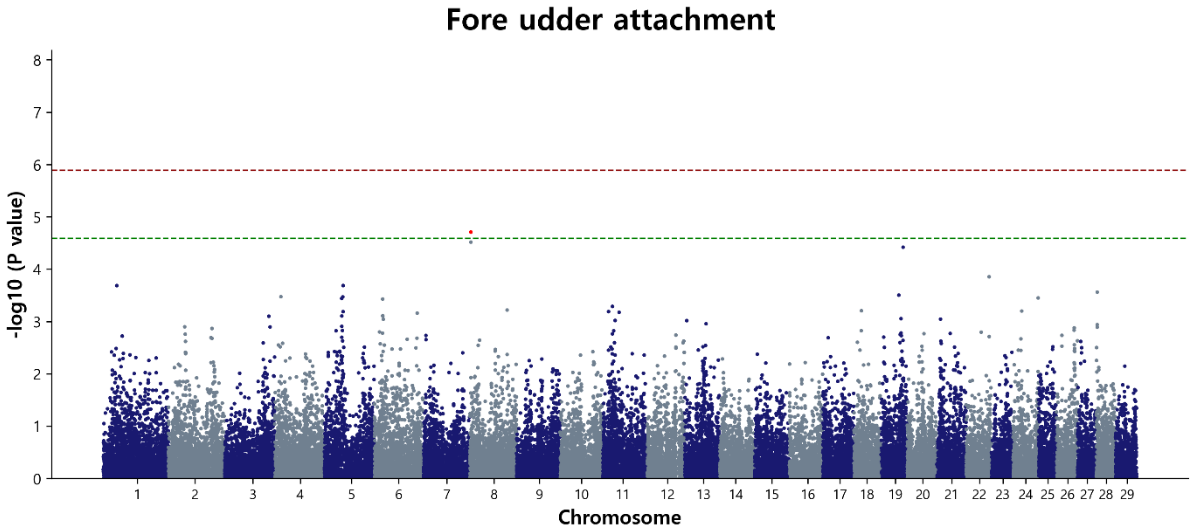
Fore udder attachment
-log10 (P value)
Chromosome
0
1
2
3
4
5
6
7
8
1
2
3
4
5
6
7
8
9
10
11
12
13
14
15
16
17
18
19
20
21
22
23
24
25
26
27
28
29

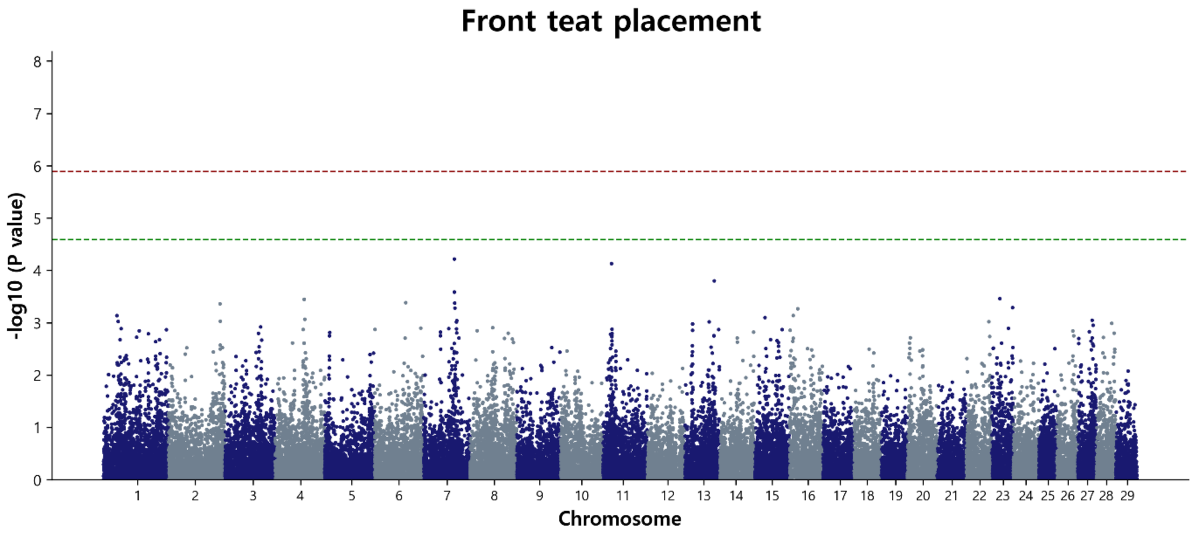

Front teat placement
-log10 (P value)
0
1
2
3
4
5
6
7
8
1 2 3 4 5 6 7 8 9 10 11 12 13 14 15 16 17 18 19 20 21 22 23 24 25 26 27 28 29
Chromosome

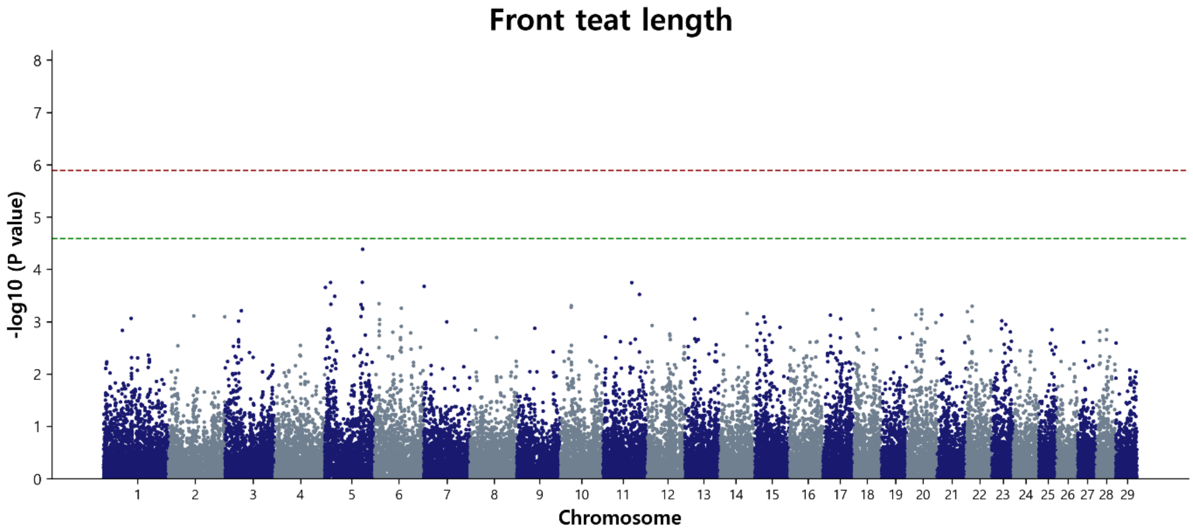

Front teat length
-log10 (P value)
0
1
2
3
4
5
6
7
8
1 2 3 4 5 6 7 8 9 10 11 12 13 14 15 16 17 18 19 20 21 22 23 24 25 26 27 28 29
Chromosome

Rear udder height

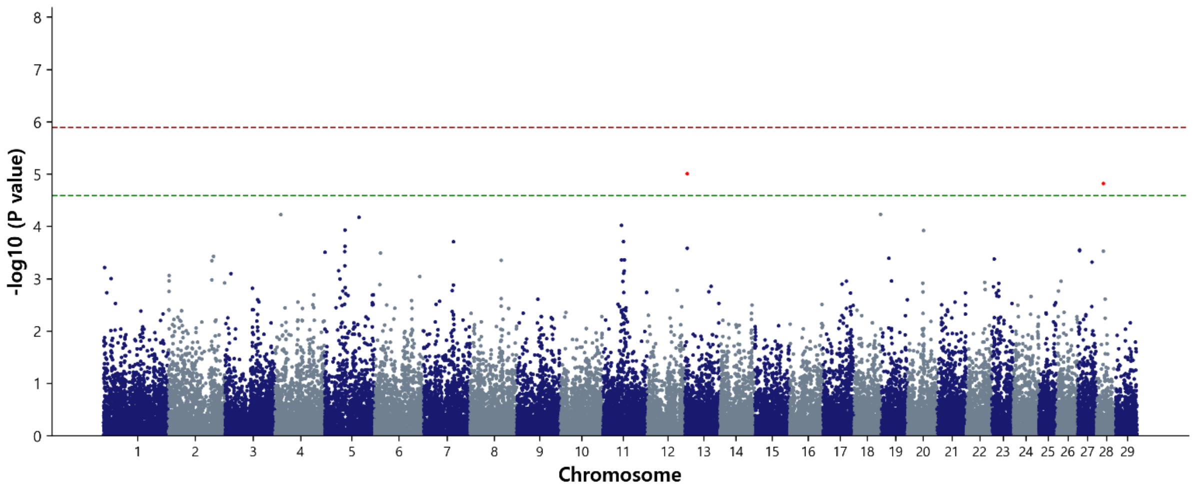

Rear udder width

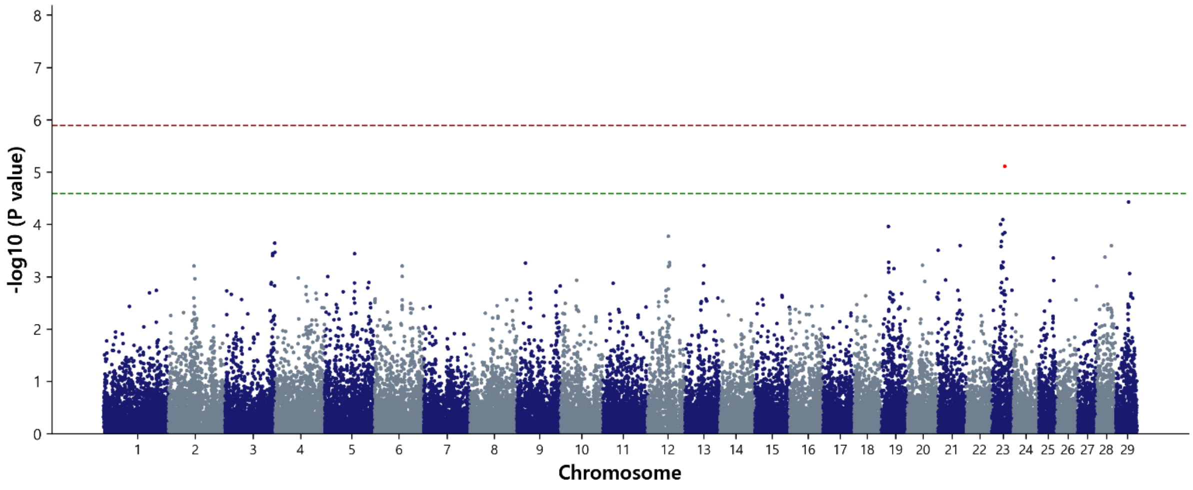

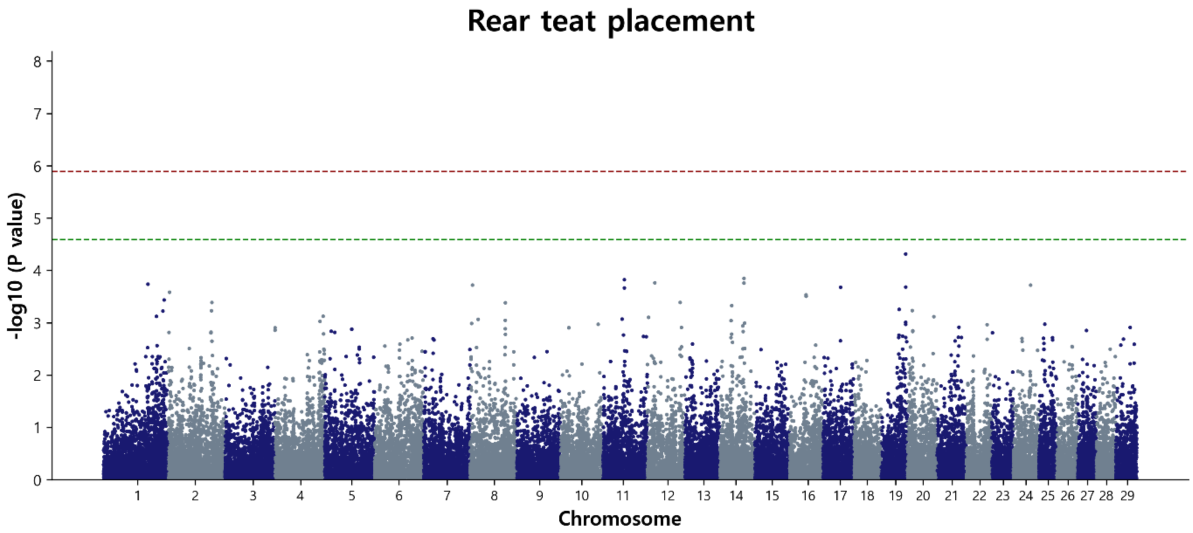

Figure S10. Manhattan plots of genome-wide -log10 (*p*-values) for udder traits in Holstein cattle. The X-axis represents the 29 Bos taurus autosomes, and the Y-axis represents the -log10P values. The horizontal dashed line in green indicates the suggestive significance threshold at $p = 2.58 \times 10^{-5}$ (4.59). The genome-wide threshold (red dashed line) corresponds to the Bonferroni correction at $p = 1.29 \times 10^{-6}$ (5.89).

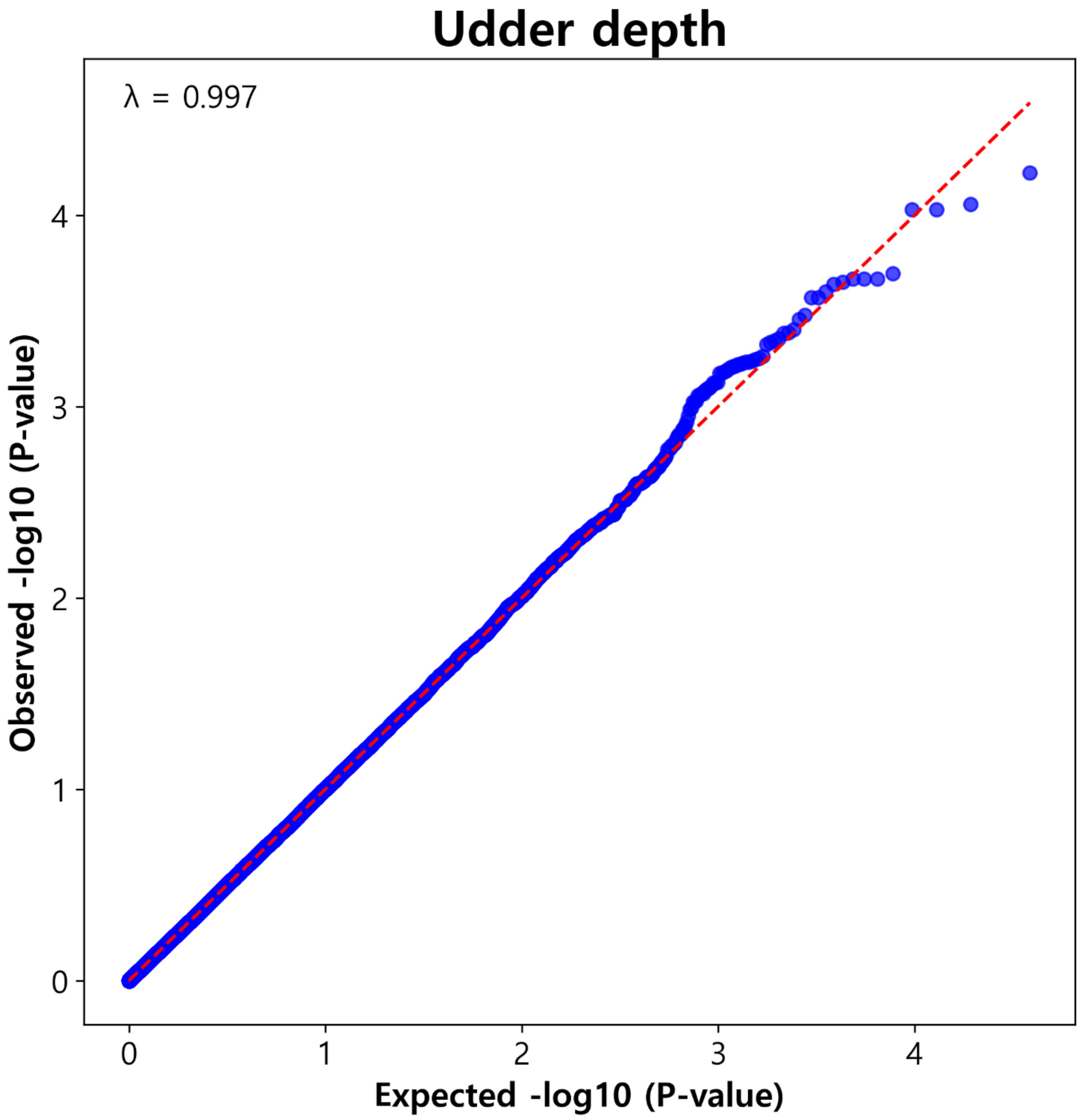

Udder depth
λ = 0.997
Observed -log10 (P-value)
Expected -log10 (P-value)
0
1
2
3
4

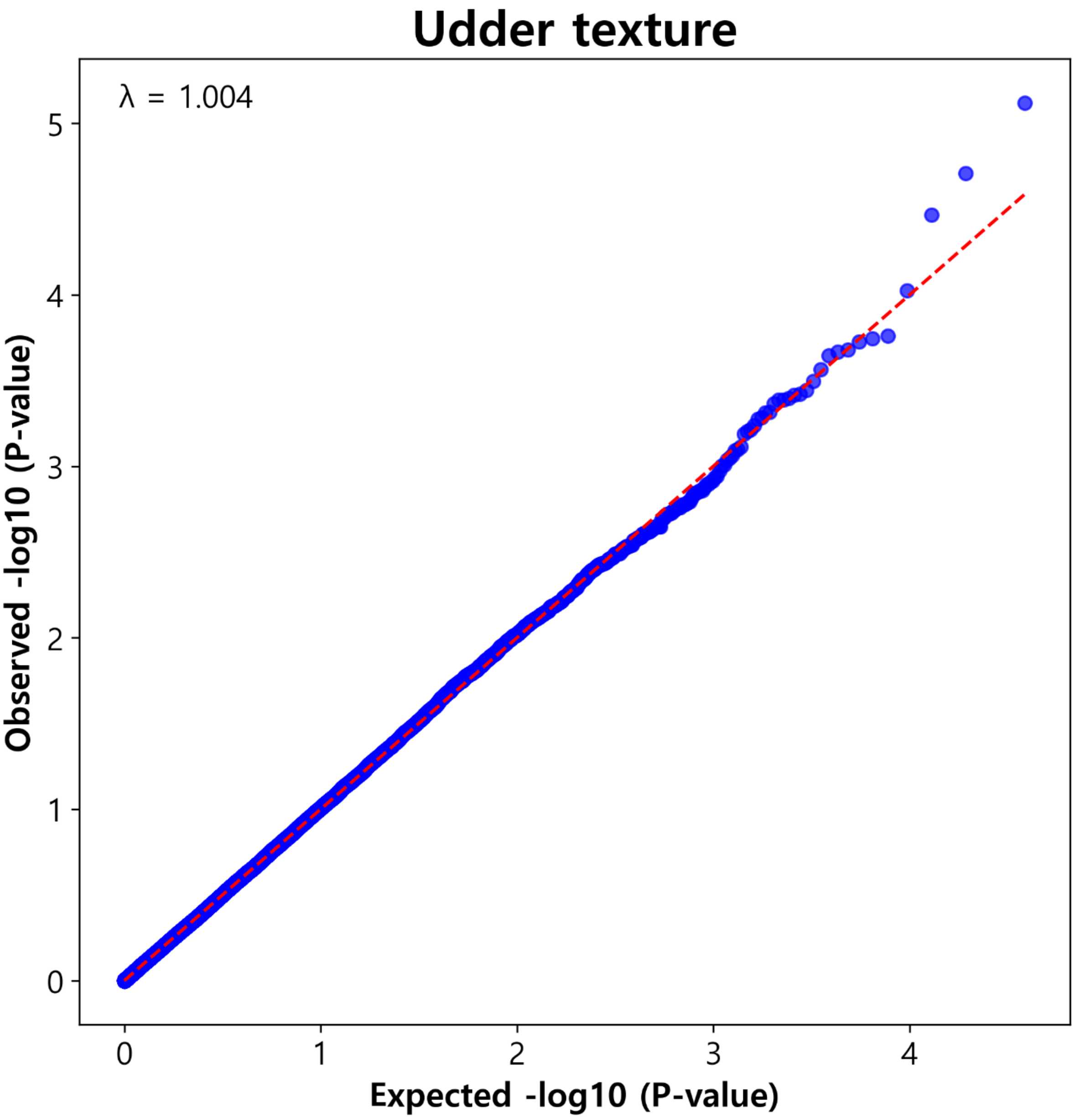
Udder texture
λ = 1.004
Observed -log10 (P-value)
Expected -log10 (P-value)
0
1
2
3
4
5
0
1
2
3
4

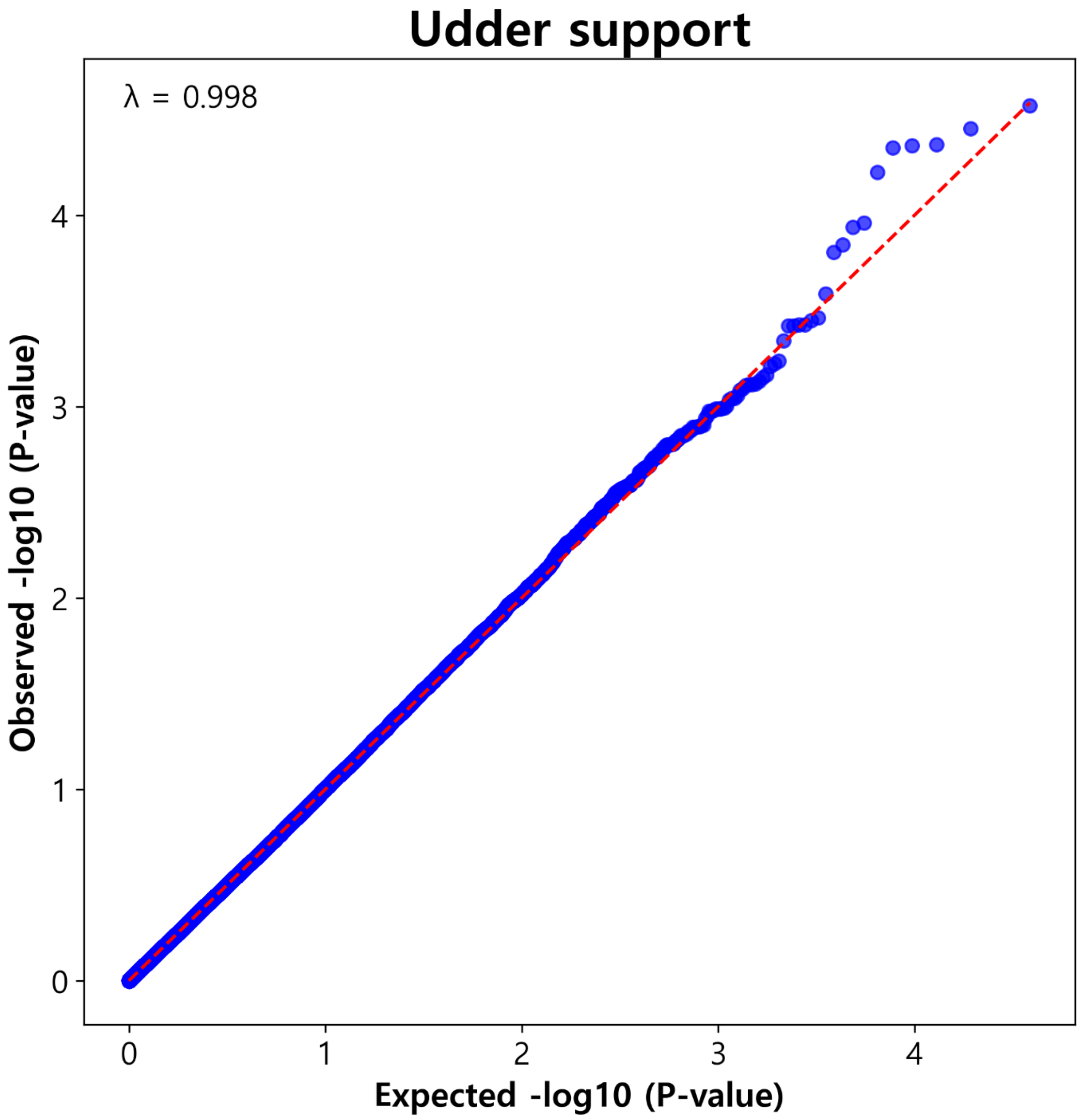

Udder support
λ = 0.998
Observed -log10 (P-value)
Expected -log10 (P-value)
0
1
2
3
4

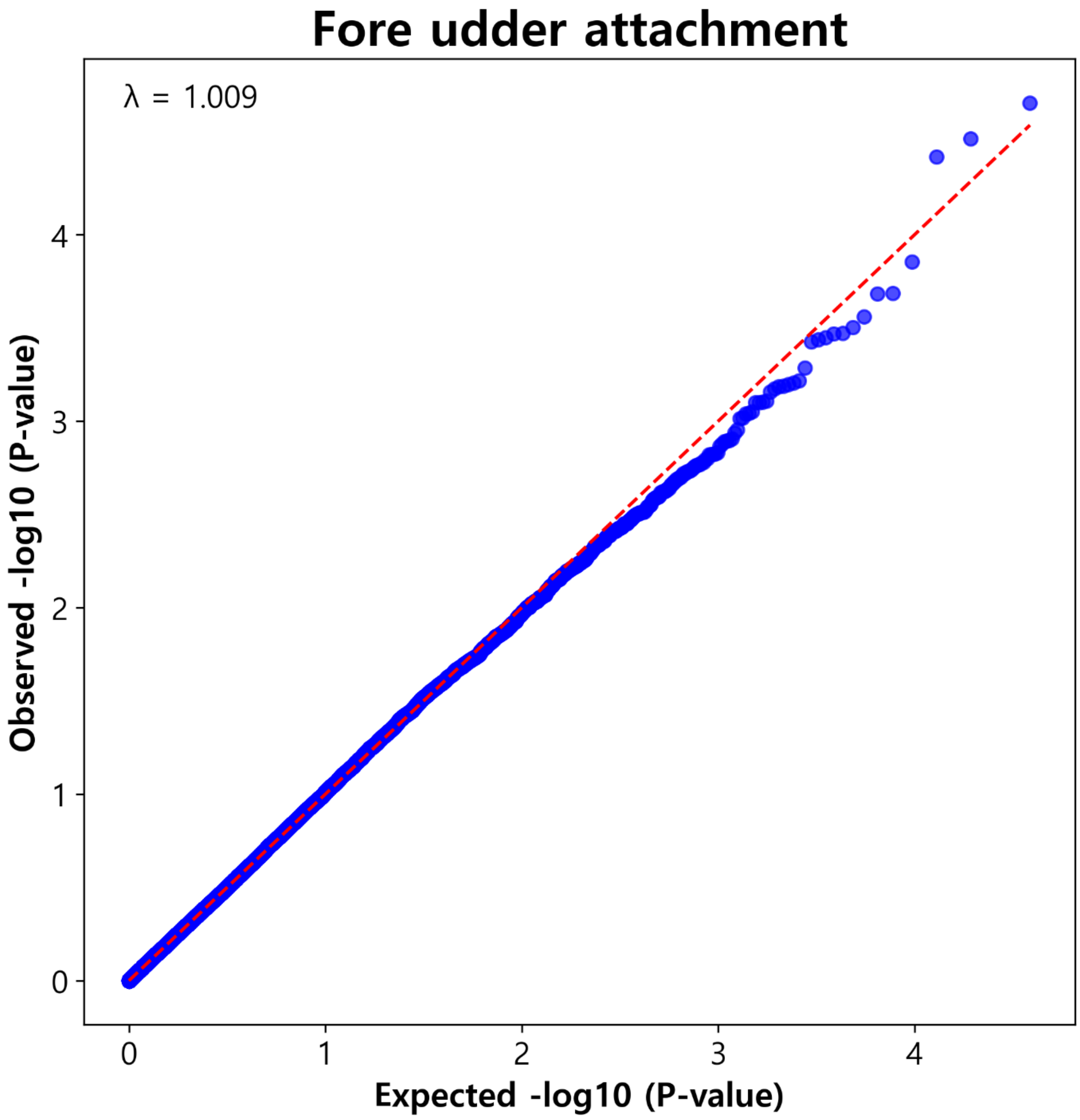
Fore udder attachment
λ = 1.009
Observed -log10 (P-value)
Expected -log10 (P-value)
0
1
2
3
4
0
1
2
3
4

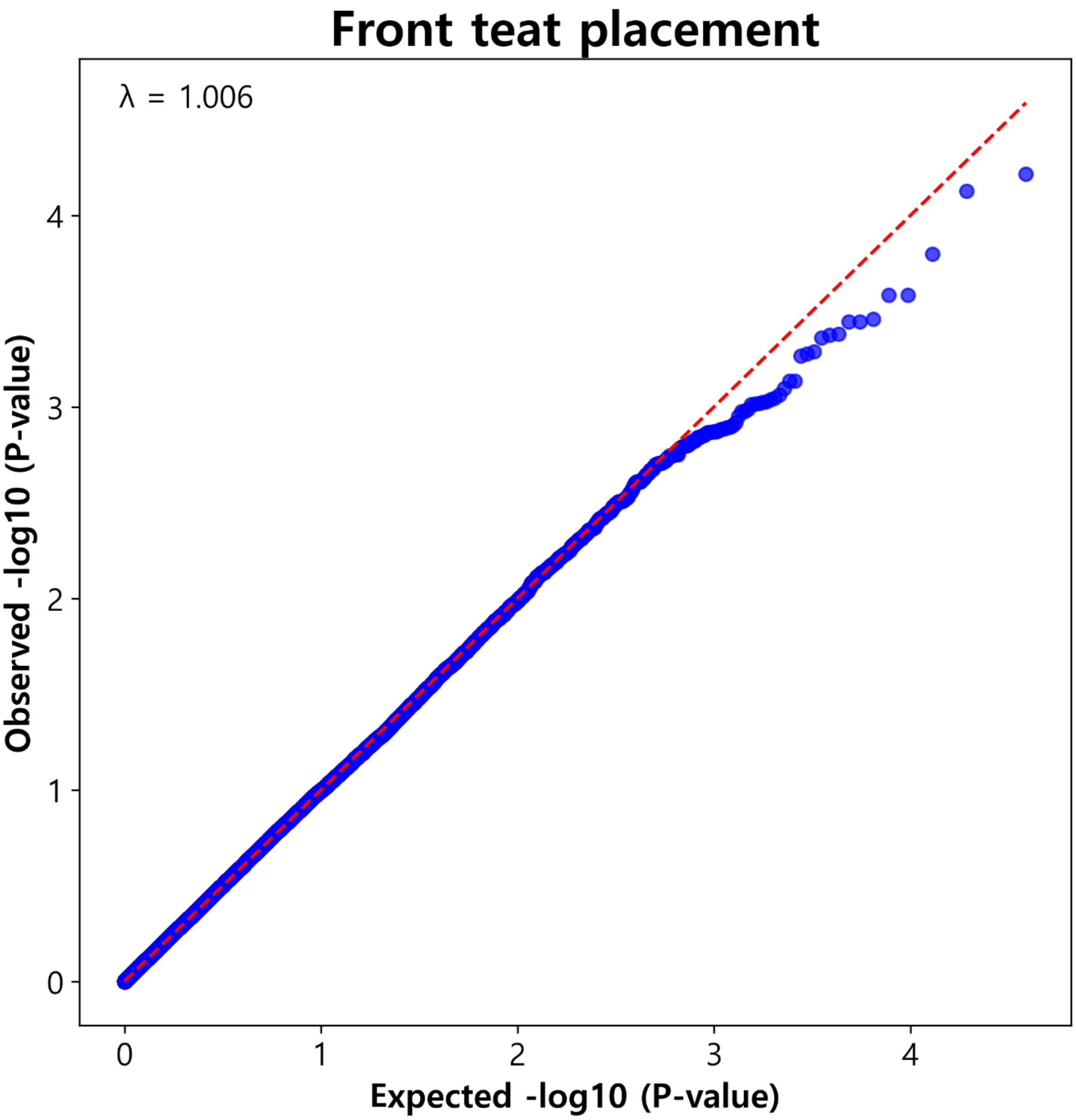

Front teat placement
λ = 1.006
Observed -log10 (P-value)
Expected -log10 (P-value)
0
1
2
3
4

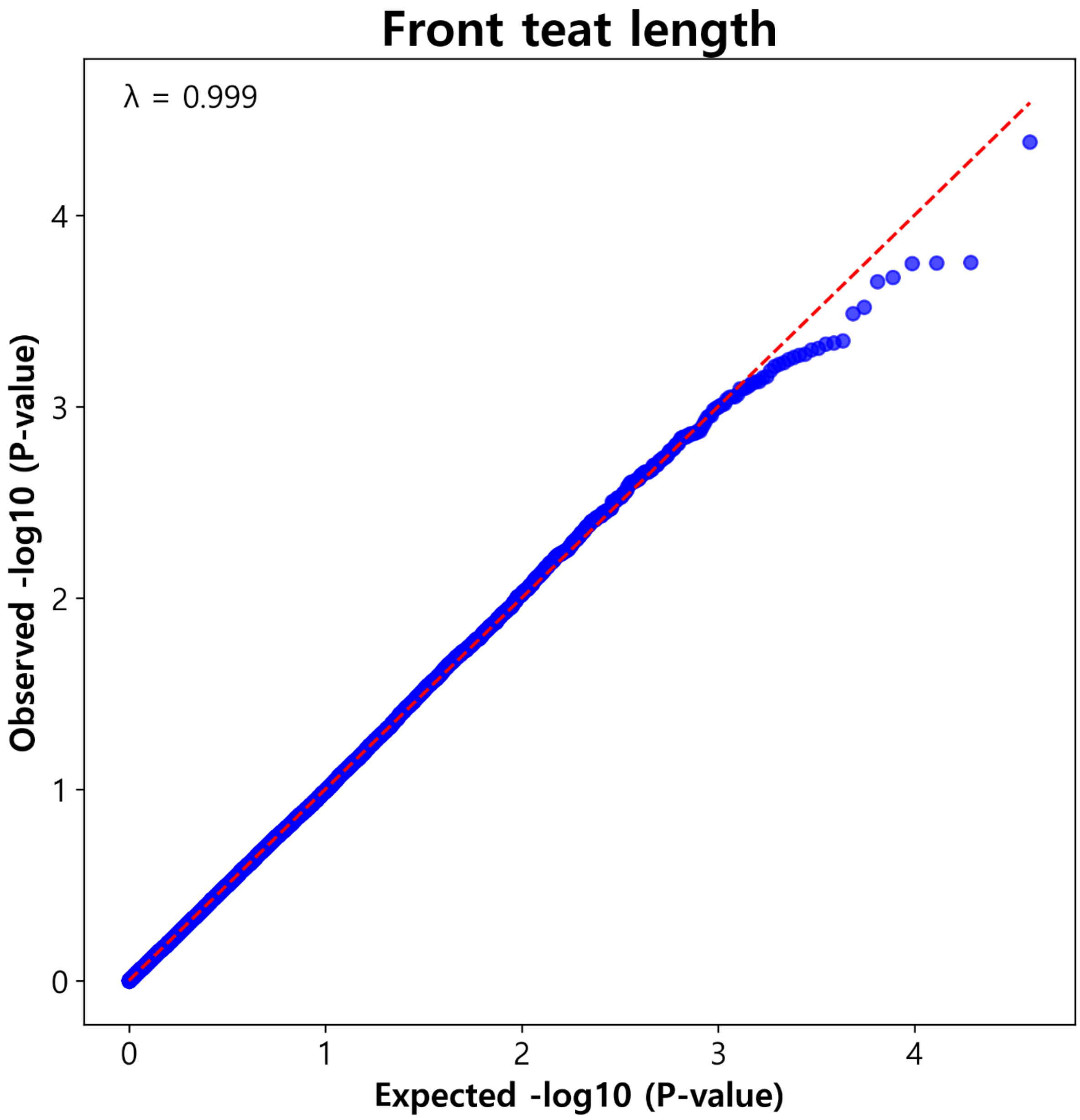

Front teat length
λ = 0.999
Observed -log10 (P-value)
Expected -log10 (P-value)
0
1
2
3
4

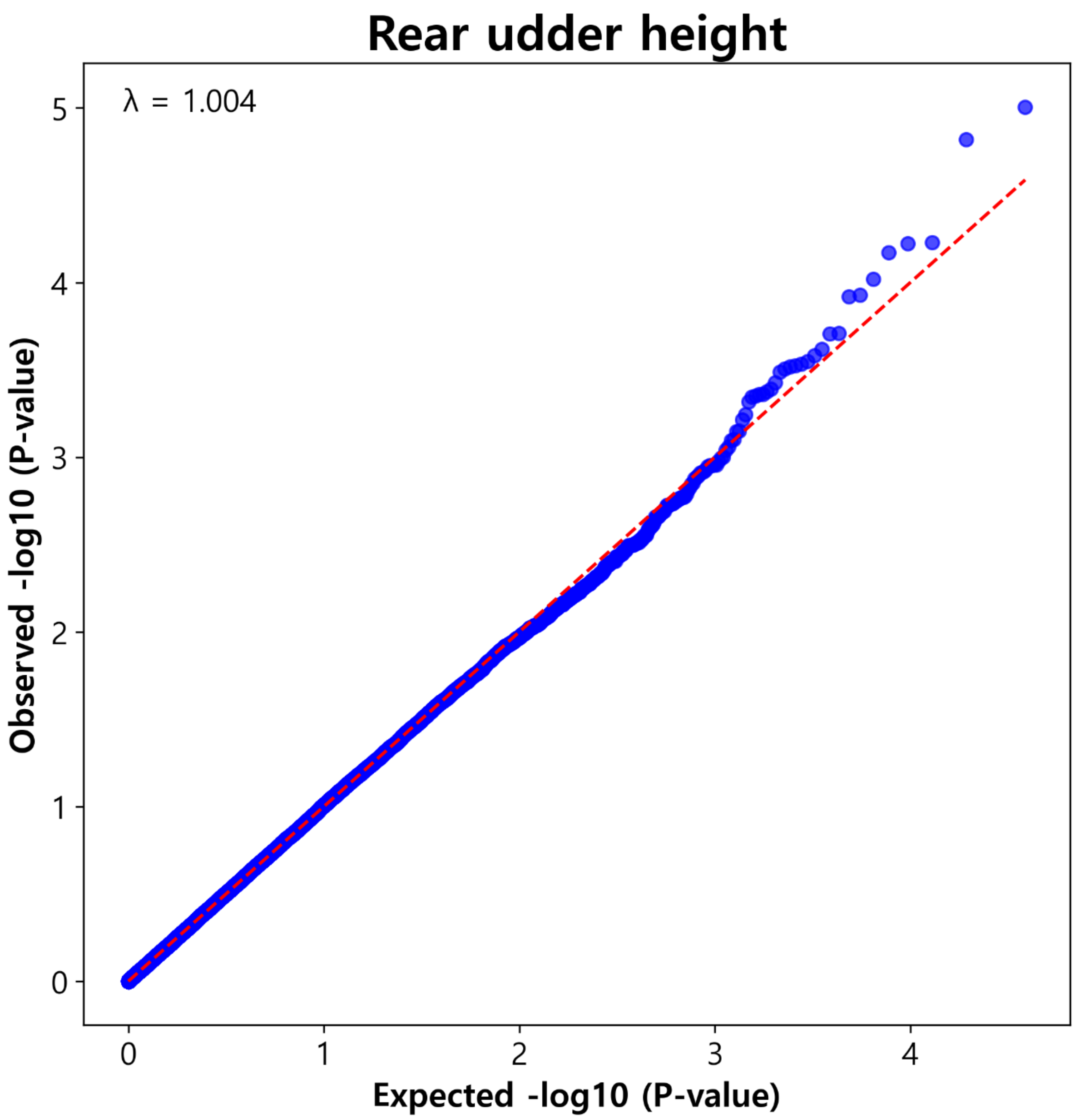
Rear udder height
λ = 1.004
Observed -log10 (P-value)
Expected -log10 (P-value)
0
1
2
3
4
5

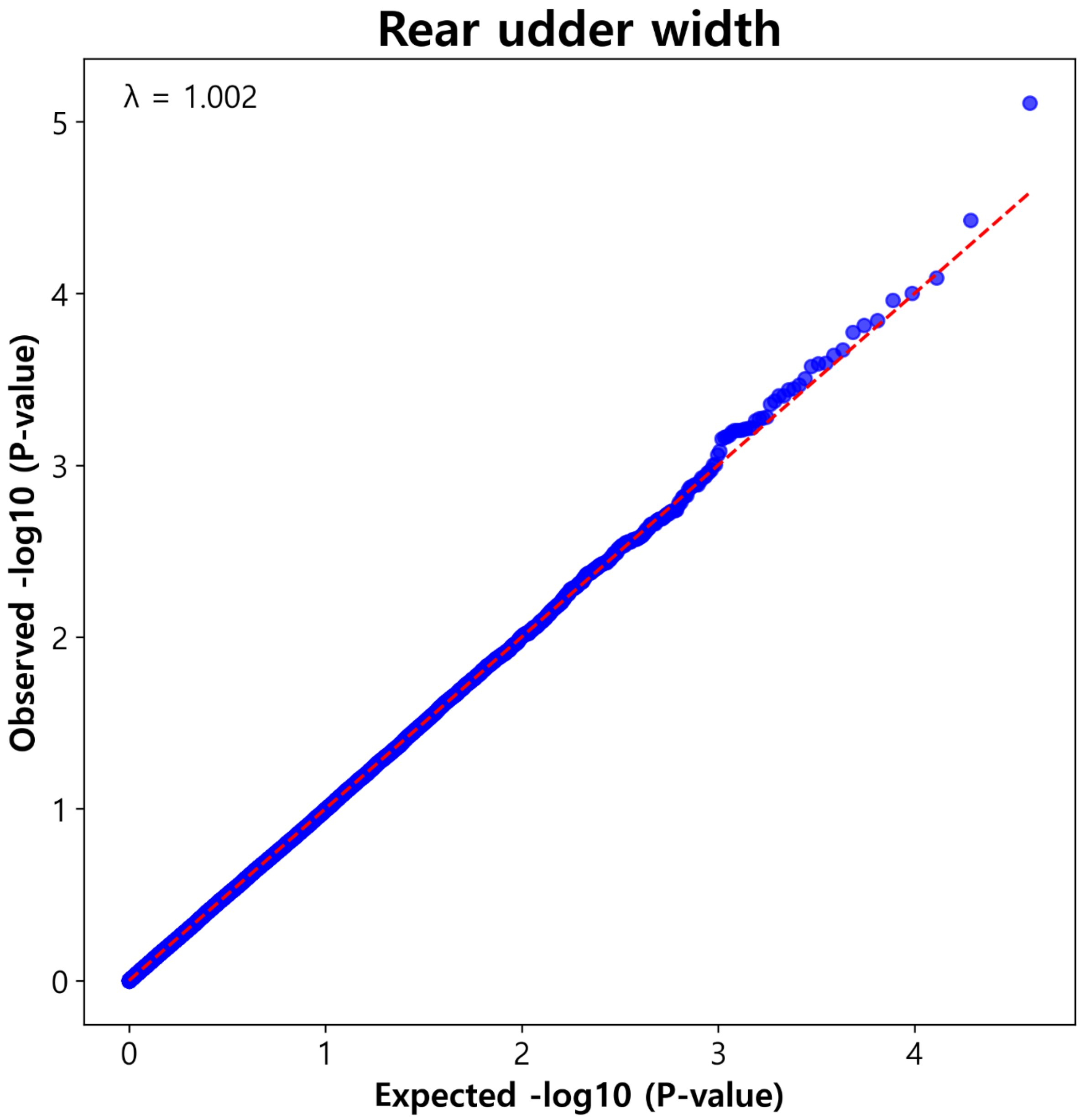
Rear udder width
λ = 1.002
Observed -log10 (P-value)
Expected -log10 (P-value)
0
1
2
3
4
5
0
1
2
3
4

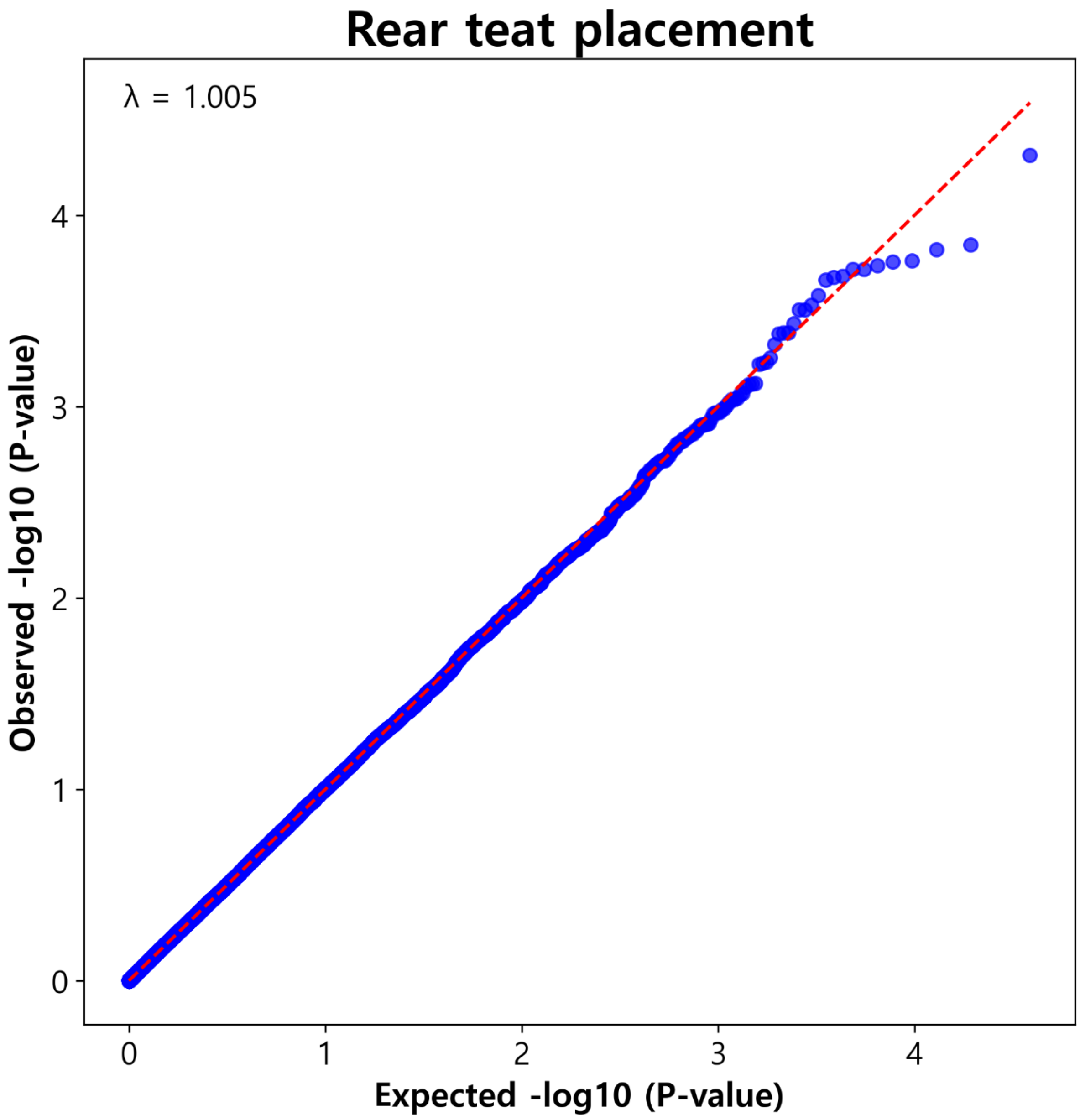

Figure S11. Quantile-quantile (Q-Q) plots and genomic inflation factor (λ) of the GWAS analysis for udder traits in Korean Holstein. QQ plots showing the late separation between observed and expected *p*-values (-log10P). Genomic inflation factor (λ) is around 1 indicating that there is no population stratification.

Table S1. GO terms from DAVID software significantly enriched using candidate genes associated with the body conformation traits.

Category	Term ID	Term Name	Count	%	PValue	Fold Enrichment	Bonferroni	Benjamini	FDR
Biological process	GO:0007411	axon guidance	4	0.61100	0.00414	5.65441	0.95398	1.00000	1.00000
	GO:0016477	cell migration	6	0.71283	0.00455	4.50838	0.96632	1.00000	1.00000
	GO:0048490	anterograde synaptic vesicle transport	3	0.30550	0.00665	24.03125	0.99297	1.00000	1.00000
	GO:0030324	lung development	4	0.40733	0.01076	8.68927	0.99968	1.00000	1.00000
	GO:0007162	negative regulation of cell adhesion	3	0.30550	0.01972	13.73214	1.00000	1.00000	1.00000
	GO:0048015	phosphatidylinositol-mediated signaling	3	0.30550	0.03158	10.68056	1.00000	1.00000	1.00000
	GO:0002088	lens development in camera-type eye	3	0.30550	0.03322	10.39189	1.00000	1.00000	1.00000
	GO:0071805	potassium ion transmembrane transport	4	0.40733	0.03461	5.57246	1.00000	1.00000	1.00000
	GO:0034765	regulation of ion transmembrane transport	4	0.40733	0.03653	5.45390	1.00000	1.00000	1.00000
	GO:0046854	phosphatidylinositol phosphorylation	3	0.30550	0.04556	8.73864	1.00000	1.00000	1.00000
	GO:0060155	platelet dense granule organization	2	0.20367	0.04558	42.72222	1.00000	1.00000	1.00000
Cellular component	GO:0008076	voltage-gated potassium channel complex	4	0.50916	0.00076	12.10843	0.14857	0.10097	0.09859
	GO:0009986	cell surface	4	1.01833	0.00095	3.96276	0.18294	0.10097	0.09859
	GO:0005856	cytoskeleton	5	0.81466	0.00477	3.85025	0.63695	0.24549	0.23970
	GO:0005887	integral component of plasma membrane	7	1.42566	0.00579	2.37854	0.70800	0.24549	0.23970
	GO:0005737	cytoplasm	9	3.86965	0.00993	1.47745	0.87955	0.29390	0.28697
	GO:0030659	cytoplasmic vesicle membrane	4	0.40733	0.01248	8.24682	0.93017	0.29390	0.28697
	GO:0005794	Golgi apparatus	3	1.12016	0.02481	2.23764	0.99514	0.47816	0.46688
Molecular function	GO:0005212	structural constituent of eye lens	3	0.30550	0.01207	17.77355	0.95946	1.00000	1.00000
	GO:0042802	identical protein binding	6	1.42566	0.01832	2.04495	0.99241	1.00000	1.00000
	GO:0042731	PH domain binding	2	0.20367	0.02652	74.05645	0.99917	1.00000	1.00000
	GO:0035091	phosphatidylinositol binding	3	0.40733	0.02888	5.98436	0.99956	1.00000	1.00000
	GO:0098882	structural constituent of presynaptic active zone	2	0.20367	0.03304	59.24516	0.99986	1.00000	1.00000
	GO:0097110	scaffold protein binding	3	0.30550	0.03954	9.45402	0.99998	1.00000	1.00000
